# Exploring New Structural Features of the 18β-Glycyrrhetinic Acid Scaffold for the Inhibition of Anaplastic Lymphoma Kinase

**DOI:** 10.3390/molecules24193631

**Published:** 2019-10-08

**Authors:** Dong Cai, ZhiHua Zhang, Yu Chen, YanYan Zhang, YuQi Sun, YiXia Gong

**Affiliations:** 1 College of Public Basic Sciences, Jinzhou Medical University, Jinzhou 121001, China; caidong0804@163.com; 2 School of Chemical and Environmental Engineering, Liaoning University of Technology, Jinzhou 121001, China; bridge1026@163.com; 3 School of Life Science and Biopharmaceutics, Shenyang Pharmaceutical University, Shenyang 110016, China; gzweishengwu@126.com; 4 College of Pharmacy, Jinzhou Medical University, Jinzhou 121001, China; zyy19981231@sina.com

**Keywords:** 18β-glycyrrhetinic acid, carbamate moiety, antitumor inhibitors

## Abstract

Novel 18β-glycyrrhetinic acid derivatives possessing a carbamate moiety and structurally similar ester derivatives were developed and evaluated for their efficacy as antitumor inhibitors. In the cellular assays, most of the *N*-substituted carbamate derivatives at the C3-position exhibited potent activities. The results of SAR investigation revealed that the introduction of the morpholine group at the C30-COOH led to a significant loss of the inhibitory potency. Among the ester derivatives, the ester group at C3-position also determined a noticeable reduction in the efficacy. Compound **3j** exhibited the most prominent antiproliferative activity against six human cancer cells (A549, HT29, HepG2, MCF-7, PC-3, and Karpas299). Furthermore, compound **3j** exerted a moderate inhibiting effect on the ALK. The results of molecular docking analyses suggested that it could bind well to the active site of the receptor ALK, which was consistent with the biological data. These results might inspire further structural optimization of 18β-glycyrrhetinic acid aiming at the development of potent antitumor agents. The structures **4d**, **4g**, **4h**, **4j**, and **4n** were studied by X-ray crystallographic analyses.

## 1. Introduction

Glycyrrhetinic acid (GA) is a major bioactive triterpenoid metabolite of glycyrrhizin (GL) extracted from the roots of *Glycyrrhiza uralensis* Fisch (a particular species of licorice). GA exists as 18α and 18β-GA stereo-isomeric forms. Chemical structural differences between 18α-GA and 18β-GA lie in the spatial arrangement of hydrogen atom of C18-position. 18β-GA has been most extensively investigated and used not only for its abundance in root extract, but also the structural resemblance between 18β-GA and corticosteroids [1]. 18β-GA derivatives exhibit diverse pharmacological properties, such as anti-inflammation [2], antiulcer [3], antivirus [4], antitumor [5], antihepatotoxic [6], antibacterial [7], and antidiabetic activities [8].

18β-GA derivatives exhibit prominent chemopreventive activities in various experimental cancer models [9,10,11,12]. The studies reported that 18β-GA derivatives have suggested protective effects against carcinogenic and tumorigenic factors by modulating the enzymatic antioxidant system and the attachment of carcinogenic factors to DNA or their receptors.

Besides, the proapoptotic mechanisms of 18β-GA have been extensively studied over the past few decades. 18β-GA derivatives display anti-proliferative and pro-apoptotic effects against human pituitary adenoma cells (GH3, MMQ) [13], breast cancer (MCF-7) [14], prostate cancer (DU-145) [15], ovarian cancer (SiHa, SK-OV-3, OVKAR-3) [16], lung cancer (A549, NCI-H460) [17], promyelotic leukemia (HL-60) [9], stomach cancer (KATO III) [18], hepatic cancer cells (HepG2, LX-2) [9,18], etc. The direct effects of 18β-GA derivatives can occur by suppressing tumor cells proliferation, with a noticeable accumulation of the tumor cells in the G1 phase, accompanied by a decrease in tumor cells in the S phase [18,19,20]. The antiproliferative activity transforms into cytotoxic effect when cell cycle arrest persists for long durations on several cancer lines [18]. There are also some 18β-GA derivatives that can exert anti-migratory and anti-invasive activities in human breast cancer cells (MDA-MB-231, MDA-MB-436) [21].

**Figure 1:**
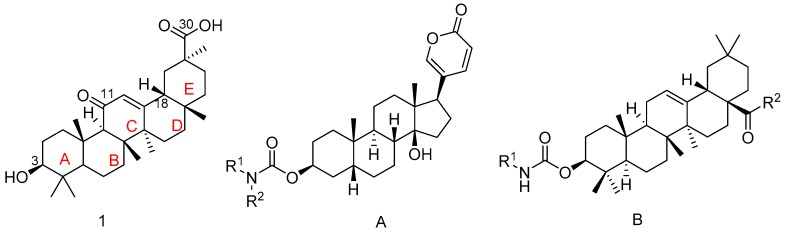
Structure of 18β-GA **1** and known derivatives **A** and **B**.

18β-GA has been adopted as an attractive molecular scaffold to search for potential antitumor inhibitors. Current structural optimization of 18β-GA leading to antitumor agents primarily focused on modification of the C3-OH in ring A, 11-one in ring C, C30-COOH in ring-E and/or multi-fragment modified simultaneously (Figure 1). The results of SAR analyses revealed that the C3-OH is a critical structural feature. The modifications at the C3-OH, reducing the polarity of the entire molecule, resulted in the significant enhancement in the in vitro antiproliferative activity. Esterification of the C3-OH group induced an enhanced inhibition of chymotrypsin-like, trypsin-like, and caspase-like activities of the 20S proteasome [22,23]. Furthermore, the introduction of side chains containing substituted amino groups in the C3-OH position significantly affected the cytotoxic activities [24,25,26,27,28].

Carbamate derivatives (e.g., the steroid skeleton) have aroused scientific interest over the years for their antitumor activities [29,30,31,32,33]. This is because carbamate moiety can form extensive hydrophobic and hydrogen bonding interactions with binding sites. Bufalin-3-yl nitrogen containing carbamate derivative **A** exhibits robust antiproliferative activities. Oleanolic acid derivatives **B** partially act as dual inhibitors for both topoisomerase I and IIa [34]. According to the results, the carbamate moiety at C3-position had vital effect on the activity [35].

To enhance antiproliferative activity of 18β-GA, a series of novel 18β-GA derivatives possessing a carbamate moiety was synthesized to delve into the effect of structural modifications at the positions of C3-OH and C30-COOH. Additional similar derivatives of esterification of the C3-OH were synthesized to explore the influence of introducing a substituted acetoxy moiety. The antiproliferative activities in vitro of the synthesized compounds were evaluated. Furthermore, docking simulation was also performed for exploring the binding mode of the active compound at the ALK active site.

## 2. Results and Discussion

### 2.1. Chemistry

**Scheme 1:**
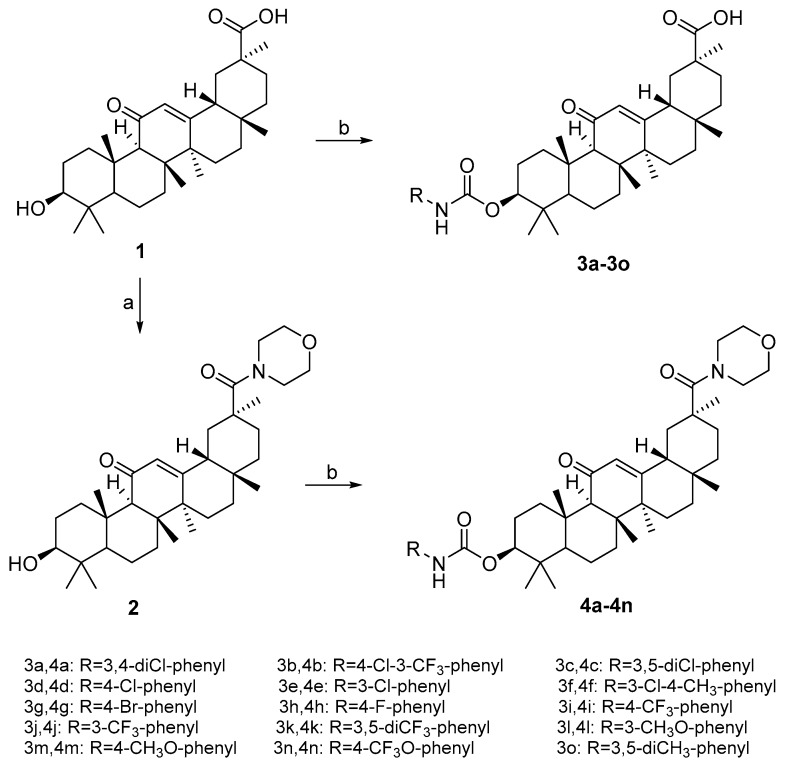
Reagents and conditions: (**a**) morpholine, EDCI, HOBt, NEt_3_, CH_3_CN, reflux, 24 h; (**b**) substituted isocyanates, ethyl acetate, reflux.

The synthetic routes to compounds **2**, **3a**–**3o**, and **4a**–**4n** are illustrated in Scheme 1. The 18β-GA **1** was activated by 1-ethyl-3-(3-dimethylaminopropyl)carbodiimide hydrochlorid (EDCI), 1-hydroxybenzotriazole (HOBt), and triethylamine under reflux for 20 min, and then it underwent amidation reaction with morpholine to form amide **2**. *O*-esterification of **1** or **2** with substituted isocyanates produced carbamate derivatives **3a**–**3o** or **4a**–**4n**, respectively.

**Figure 2:**
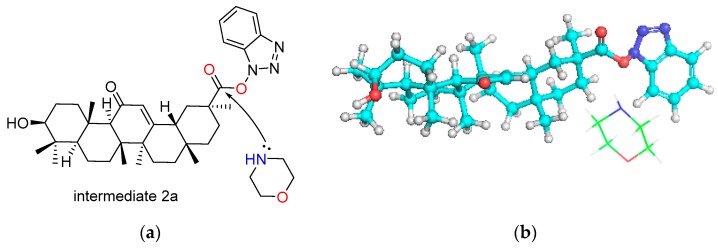
(**a**) The proposed mechanism of amidation reaction. Energy minimization by MM2 was performed using ChemBio3D Ultra 14.0 (CambridgeSoft Corporation, 2014) software force field. (**b**) The picture of 3D structure was produced using PyMOL (Delano Scientific, USA).

When this amidation reaction was performed at ambient temperature, coupling of the commercially available 18β-GA with morpholine in the presence of EDCI, HOBt, and triethylamine initially formed the intermediate **2a**, presumably because of the significant steric hindrance of the 1*H*-benzotriazol-1-yl group (Figure 2). Such intermediate **2a** could be isolated from the reaction mixture in good yield. When the temperature rose to 80 °C, the intermediate **2a** should not be isolated and then react with morpholine to give compound **2** by refluxing in CH_3_CN for 24 h. Results suggested that the reaction conditions (e.g., temperature, time, and solution) should be thoroughly controlled to make intermediate **2a** completely transformed into amide **2**.

Condensation of the carboxylic acids with substituted isocyanates can afford unstable carbamic carboxylic anhydrides, which were transformed into *N*-substituted amides after decarboxylation in the presence of bases (most often, 4-dimethylaminopyridine [36], *N,N*-diisopropylethylamine [37], trimethylamine [38]). This condensation reaction is a well-known method for a practical synthesis of *N*-substituted amides and peptide analogues. Nevertheless, compounds **3a**–**3o** can be prepared in high yields from 18β-GA with substituted isocyanates in the absence of base catalyst. In this case, the competitive reactions of the C30-COOH and substituted isocyanates were not observed [38]. These methyl and cyclohexyl fragments in the α-position to C30-COOH provided steric hindrance that prevented steric hindrance that prevents the possible amidation reaction in this condensation. Obviously, an excess of substituted isocyanates should not be adopted in condensation for the possible amidation. Compounds **3a**–**3o** were obtained at a molar ratio of 18β-GA/substituted isocyanates (1:1.2) in refluxing ethyl acetate.

**Scheme 2:**
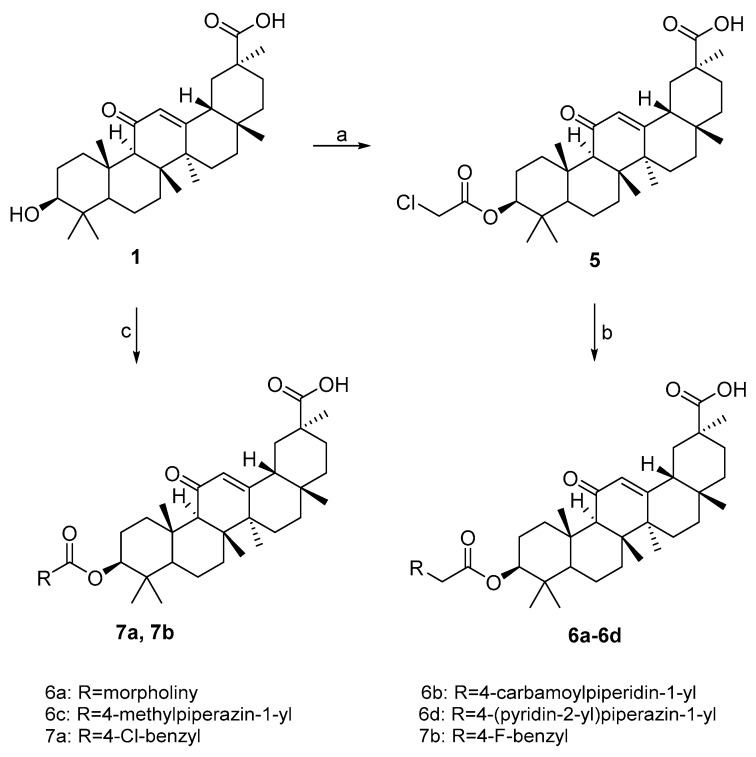
Reagents and conditions: (**a**) chloroacetic anhydride, 130 °C, 1 h; (**b**) substituted secondary amines, K_2_CO_3_/ I_2_, C_2_H_5_OH; (**c**) substituted acyl chlorides, ethyl acetate, NEt_3_, reflux, 24 h.

New derivatives of 18β-GA bearing a bulky ester moiety in position C3-OH were synthesized (Scheme 2). The hydroxyl group of 18β-GA underwent esterification with chloroacetic anhydride at 130 °C, and the resulting ester **5** was aminated with the secondary amines to obtain **6a**–**6d**. Compounds **7a**–**7b** can also be synthesized through the treatment of 18β-GA with a substituted acyl chlorides in the presence of bases (e.g., triethylamine). ^1^H-NMR and ^13^C-NMR spectra for all the prepared compounds can be seen in the Supplementary Materials.

Due to the noticeable steric hindrance at the β-position of C3-OH (Figure 2), the esterification reactions of 18β-GA with substituted isocyanates required refluxing in ethyl acetate. For the identical reason, the reactions of 18β-GA with substituted benzyl chlorides or chloroacetic anhydride are easily are also affected by steric hindrance as well.

**Figure 3:**
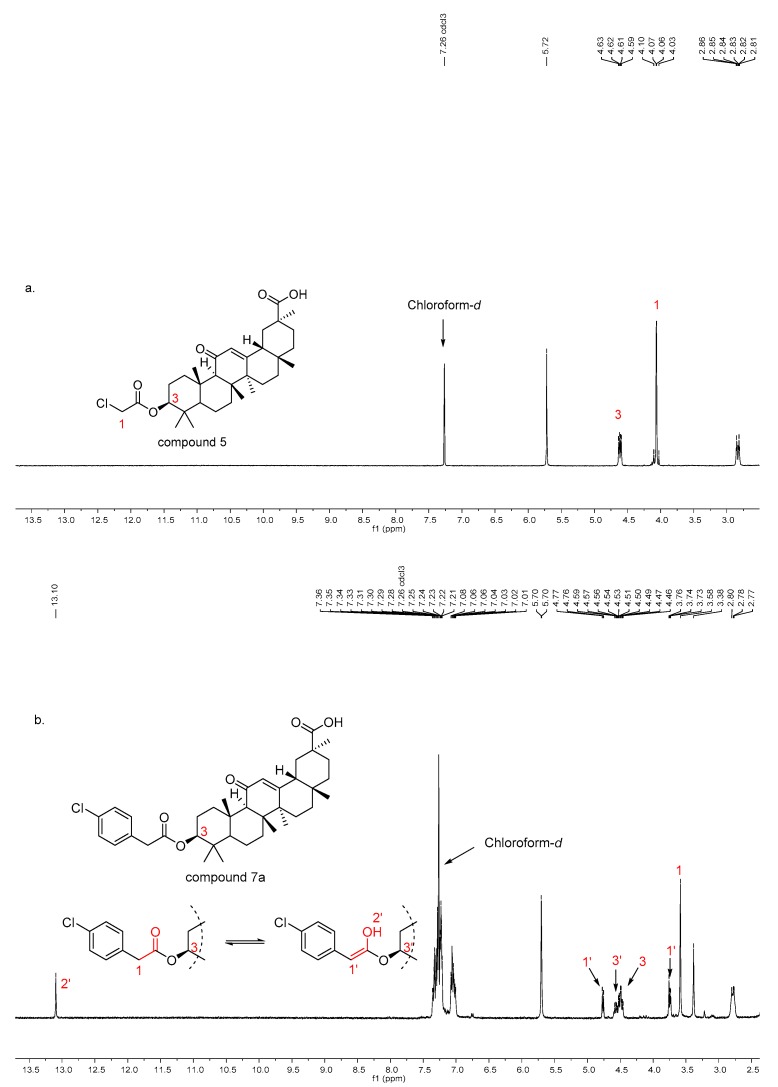
The ^1^H-NMR spectrum of (**a**): compound **5**; (**b**): compound **7a**.

On the whole, compound **5** bearing a weak electron withdrawing substituent (chloro group) at the α-position of ester group is stable, primarily existing in the form of keto tautomer. The ^1^H-NMR (400 MHz) spectrum in Chloroform-*d* of compound **5** (Figure 3a) displayed a quartet at δ 4.62 ppm attributed to the proton at C3-position and a doublet at 4.06 ppm attributed to CH_2_ protons at the α-position of ester group.

In contrast, a potent conjugation between the phenyl ring and the enol moiety of compound **7a** led to noticeable shift equilibrium toward the enol tautomer [39,40]. The ^1^H-NMR (400 MHz) spectrum in Chloroform-*d* of compound **7a** (Figure 3b) is presented here to study enolate formation. A singlet at 3.58 ppm referred to the presence of the CH_2_ protons at position 1 of keto tautomer. The structure of the enol tautomer might form both the *Z*-configuration and *E*-configuration [41]. The chemical shifts of the proton 1′ on olefin of the enol tautomer were identified at 4.76 ppm (*J* = 7.1 Hz) and at 3.74 ppm (*J* = 4.1 Hz), respectively. Moreover, a noticeable singlet observed at δ 13.10 ppm was attributed to hydroxyl group of the enol tautomer. A small multiplet observed at 4.54–4.60 ppm (near proton at C3-position in the keto tautomer, of which the signal was observed at 4.46–4.52 ppm) could represent the proton at C3′-position of enol tautomer.

### 2.2. Subsection Crystal Structure Analysis of **4d**, **4g**, **4h**, **4j**, and **4n**

**Figure 4:**
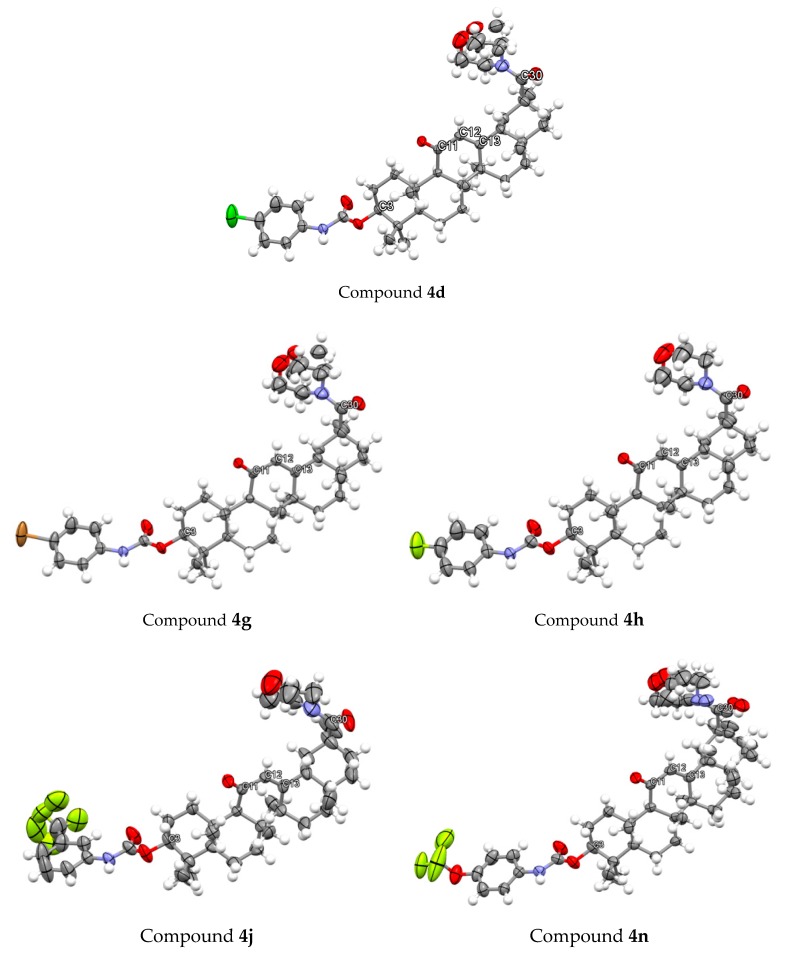
ORTEP diagram of compounds **4d**, **4g**, **4h**, **4j**, and **4n** at 50% probability.

The ORTEP of the compounds **4d**, **4g**, **4h**, **4j**, and **4n** with thermal ellipsoids at 50% probability is shown in Figure 4. Table 1 represents crystal and experimental data of the molecules compound **4d**, **4g**, **4h**, **4j**, and **4n**. Crystallographic data have been deposited in the Cambridge crystallographic data Center. “CCDC 1953934, 1953928, 1953941, 1913193, 1952936 contain the supplementary crystallographic data for this paper. These data can be obtained free of charge via http://www.ccdc.cam.ac.uk/conts/retrieving.html (or from the CCDC, 12 Union Road, Cambridge CB2 1EZ, UK; Fax: +44 1223 336033; E-mail: deposit@ccdc.cam.ac.uk)”.

**Figure 5:**
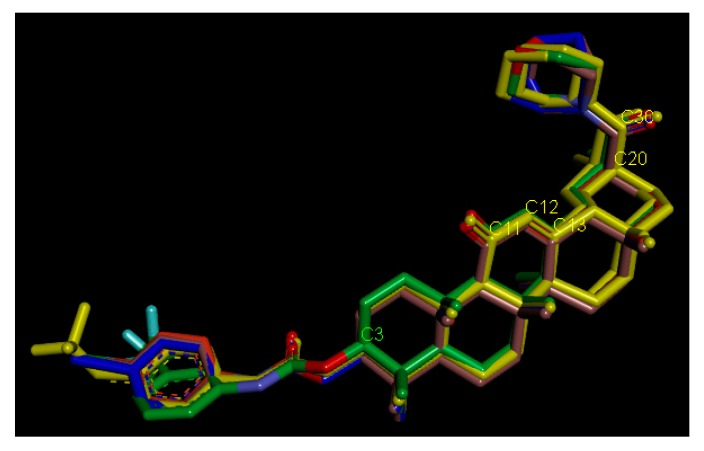
3D superimposition crystal structure of compounds **4d** (brown), **4g** (blue), **4h** (red), **4j** (green), and **4n** (yellow).

The 3D superimposition of compounds **4d**, **4g**, **4h**, **4j**, and **4n** (Figure 5) revealed that the geometries of the two structural scaffolds are virtually identical, and the main structural difference between these five structures is in orientation of the phenyl rings in the carbamate side chain.

**Figure 6:**
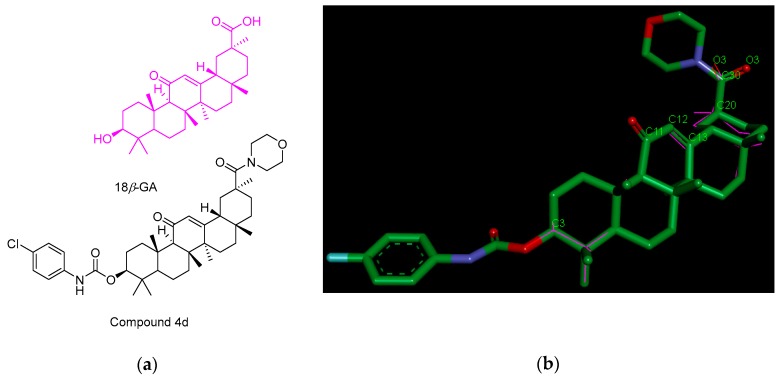
(**a**) Molecular structures and (**b**) overlay crystal structures of 18β-GA (Lines) compound **4d** (Sticks).

In contrast to the X-ray crystal structure of 18β-GA from acetone/H_2_O [42], the two structural scaffolds are perfectly superimposable, but the orientation of carbonyl groups at the 30-position are slightly different. The overlay diagrams of both conformers are depicted in Figure 6. These two pictures were produced using the ‘molecular overlay’ feature of Accelrys Discovery Studio, which gives an automated optimum overlay of a number of small molecules using equally weighted steric and electrostatic fields.

### 2.3. In Vitro Cell Growth Inhibitoty Activity

The antiproliferative activities of all the synthesized compounds against HT-29 and A549 cells were evaluated by MTT assay. The growth inhibition of cancer cell line (%) from these tests are listed in Table 2.

In contrast to the results for the compounds **3a**–**3o**, the C30-COOH was converted into a morpholine amide derivatives **4a**–**4n** produced a marked loss of the inhibitory potency. The results listed in Table 1 demonstrated that the C30-COOH group in carbamate derivatives led to the potent antiproliferative activity of target compounds.

Table 2 evidently shows that the compounds **3a**–**3o** exhibited excellent antiproliferative activity against HT-29 and A549 cells, especially at the concentration of 20 μg/mL. Compared with Crizotinib, a powerful anticancer drug as positive control, compounds (**3a**, **3c**, **3e**, **3f**, **3i**, **3j**, **3k**, **3n**, **3n**, and **3o)** exhibited cell growth inhibitory activity nearly the same as Crizotinib to HT-29 cells at the identical concentration of 20 μg/mL. It is noteworthy that these active compounds **3a**–**3l**, and **3o** either possess electron-withdrawing groups (e.g., F, Cl, Br, CF_3_O, and CF_3_) or weak electron-donating group (e.g., CH_3_) on their aromatic rings. In the meantime, compounds (**3l**, **3m**), containing CH_3_O group, displayed a dramatic decrease in the activity, suggesting that the strong electron-donating group is not recommended.

Besides, introducing halogen atom into the compounds can improve selectivity, intrinsic potency, and so on. In this paper, compounds containing halogen atom in their structure have shown better anticancer activity. Comparing the derivatives with various chloro-substitution positions on the phenyl ring, compounds (**3a**, **3b**, **3c**, **3e**, **3f**) containing 2,4-diCl, 4-Cl-3-CF_3_-, 3,5-diCl, 3-Cl, and 3-Cl-4-CH_3_ groups displayed better activity, whereas 4-Cl compounds **3d** resulted in a slight decrease in potency. A similar phenomenon was observed for 4-bromo substituted compound **3g** and 4-fluoro substituted compound **3h**. It is therefore speculated that the presence of the chloro, bromo, and fluoro substituent in para-position of phenyl rings stimulated a detrimental effect on inhibitory activity of cancer cells.

The carbamate moiety at the C3-position is considered to be critical to compounds **3a**–**3o**. With the introduction of six-membered ring secondary amine groups to give compounds **6a**–**6d**, the activity was enhanced dramatically as compared with that of compounds **3a**–**3o**. In the meantime, the introduction of a substituted phenylacetoxy moiety resulted in a significant loss of cytotoxicity for the ester derivatives (**7a**, **7b**) compared with the corresponding carbamate derivatives (**3d**, **3h**).

From the results obtained, the carboxyl group at the C30-position of 18β-GA is active essential group to improve the inhibitory potency. The introduction of the substituted phenyl carbamate moiety at the C3-position led to a consistent increase in the activity. The electronic effect of substituent group and the position of the substituent group on the phenyl ring significantly influenced anticancer activity. On the whole, the substituents on the phenyl ring, carbamate moiety, and carboxyl group are critical for inhibiting the growth of tumor cells.

To deepen our research, eight compounds **3a**–**3c**, **3e**, **3j**, **3k**, **3n**, and **3o** were taken in assay for their antitumor potency indicated by IC_50_ values. Table 3 presents that compound **3j** exhibited the greatest antiproliferative activity against six human cancer cells (A549, HT29, HepG2, MCF-7, PC-3, and Karpas299) with IC_50_ values of 2.81 μg/mL, 3.19 μg/mL, 5.55 μg/mL, 5.26 μg/mL, 5.96 μg/mL, and 5.59bμg/mL, respectively. The other seven compounds also exhibited a significant antiproliferative activity against six human cancer cells. Compared with 18β-GA, introduction of a carbamate moiety at the C3-position could significantly enhance inhibitory activity.

**Figure 7:**
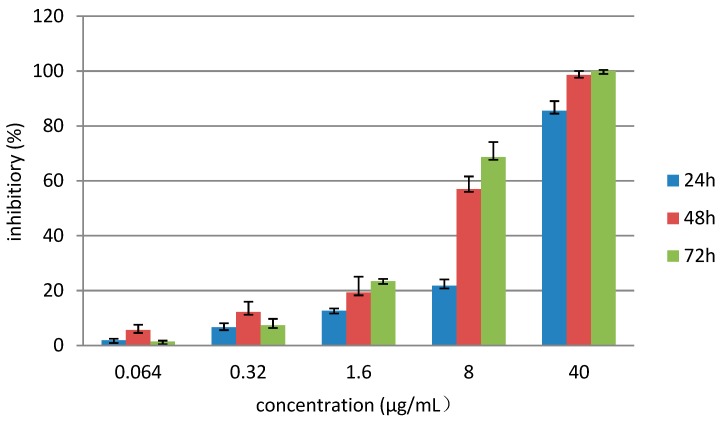
The relationship between different concentrations of compound **3j** and cytotoxicity. Data are means ± SD of the inhibition (%) from three independent experiments.

To effectively study whether compound **3j** is concentration-dependent manner, the cell was treated using MTT methods, and five concentration gradients were selected. After 24, 48, or 72 h of interaction, the inhibition rate was determined. Compound **3j** inhibited the proliferation of HepG2 cell in a significant concentration manner, whereas it did not occur in a time-dependent manner. The result was shown in Figure 7.

**Figure 8:**
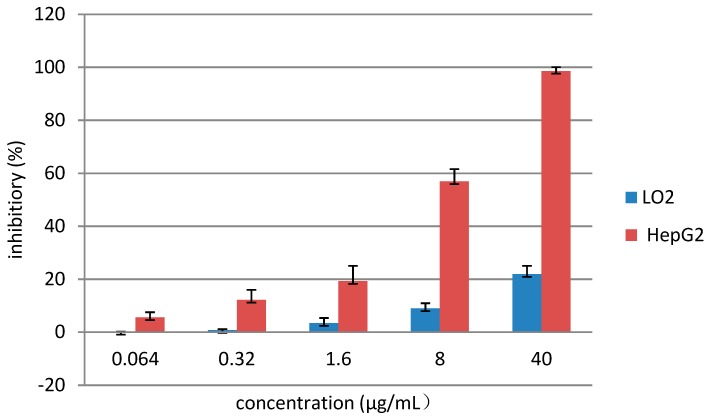
Inhibitory effects of compound **3j** on the proliferation of HepG2 and LO2 cells. Data are means ± SD of the inhibition (%) from three independent experiments.

Given the mentioned results, the most efficient compound **3j** was selected for in depth study, and it was evaluated in different concentrations (0.064–40.0 μg/mL) towards HepG2 cell line and non-tumorigenic liver LO2 cell. A 48 h continuous drug exposure protocol was employed by the MTT cytotoxicity assay. The results verified that treatment with the upregulated dose of compound **3j** had a significant inhibitory effect on HepG2 cell lines. In the meantime, compound **3j** did not exhibit significant toxic action towards LO2 cells at relatively higher concentrations, suggesting that compound **3j** might exhibit selective antiproliferative activity against human tumor cells. The result is presented in Figure 8.

### 2.4. Anaplastic Lymphoma Kinase (ALK) Activity

Based on the inhibitory activities in vitro, the compound **3j** and **3k** were taken for further evaluation of their anti-ALK potency. As shown in Table 4, the compound **3j** displayed a moderate potency against ALK enzymatic activity with an IC_50_ value of 120.68 nM, comparable to that of the positive control Crizotinib (IC_50_ = 1.64 nM). Besides, compound **3k** was less potent against the ALK with IC_50_ value of 278.21 nM. The mentioned results indicated that the inhibition of ALK is likely to be a mechanism for the antitumor effect of these carbamate derivatives, and this series of compounds further studies.

### 2.5. Molecular Docking Studies

**Figure 9:**
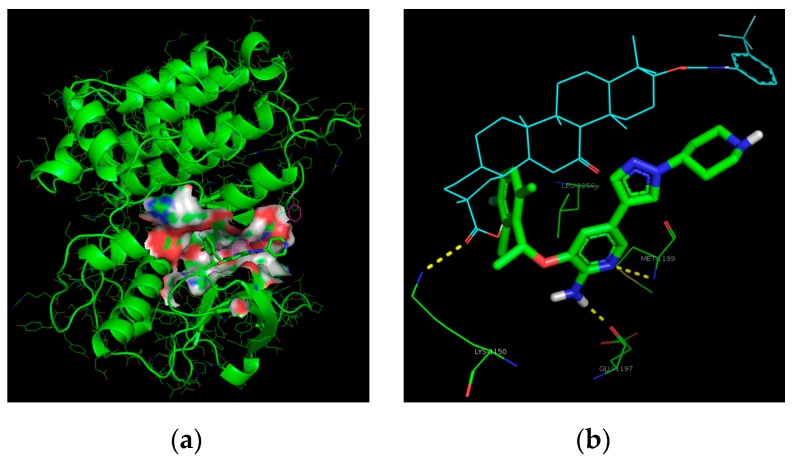
(**a**) Binding mode of Crizotinib (Stick) and the compound **3j** (Line); (**b**) prediction of contacting residues in compound-protein interfaces. Hydrogen bonds are highlighted as yellow dashes.

In order to gain the likely binding model of these carbamate derivatives with ALK, we performed docking analysis using Discovery Studio 3.5 software. The image files were generated by PyMOL. Using the crystal structure of the human ALK in complex with Crizotinib (PDB code: 2XP2), the overall binding interactions of the representative compound **3j** was shown in Figure 9. Some differences were found in the binding mode between these two ligand. The pyridine-2-amine moiety in Crizotinib bound well to Met1199 and Glu1197 residues via two hydrogen bonds, respectively. Besides, the two phenyl rings of Crizotinib might form a π–π interaction with Leu1256 residue. When compound **3j** was docked into the ALK active site, no interactions were observed with mentioned critical residues. As observed, the C30-COOH group was orientated toward the binding pocket, making a hydrogen bond with Lys1150 residue. These results comply with the moderate potency for compound **3j** inhibition of ALK. The results of the docking study also implied that the C30-COOH group is conducive to the stability of binding conformation. Nevertheless, introducing an amine group (**4a**–**4n**) might break the hydrogen bond between the ligand and the receptor, resulting in compounds (**4a**–**4n**) with poor activities in cellular assay.

## 3. Materials and Methods

### 3.1. Materials and Apparatus

Generally, all commercial reagents and solvents were used without additional purification. Analytical thin layer chromatography (TLC) was performed on Merck silica gel 60 F_254_, precoated on aluminum plates. Spot visualization was done by using use of UV light (254 nm, Shanghai Baoshan Gucun Electro-optical Instrument Factory, Shanghai, China). Melting points were recorded on a WRS-1B digital melting point apparatus (Shanghai Shenguang Instrument Co., Ltd., Shanghai, China) and were uncorrected. ^1^H- and ^13^C-NMR spectra were recorded on Agilent 400/54Premium Shielded NMR Magnet System (Agilent, Santa Clara, CA, USA) and chemical shifts were quoted in ppm, referenced to tetramethylsilane (TMS). Peak multiplicities are reported as follow: s (singlet), d (doublet), t (triplet), q (quartet), m (multiplet). The high-resolution mass spectrum (HRMS) were recorded on Agilent 6200 Series TOF and 6500 Series Q-TOF LC/MS System B.05.01. (B5125) in positive ion modes (Agilent).

### 3.2. Chemistry

#### 3.2.1. 3β-hydroxy-30-morpholino-olean-12-ene-11,30-dione **2**

The 18β-GA (0.47 g, 1.0 mmol) was dissolved in acetonitrile (20 mL), then EDCI (0.23 g, 1.2 mmol), triethylamine (0.13 g, 1.2 mmol) and HOBt (0.16 g, 1.2 mmol) were added. The mixture was stirred under reflux for 20 min. Then morpholine (0.11 g, 1.2 mmol) was added, and the mixture was stirred under reflux for 24 h. The solvent was removed under reduced pressure to give a residue which was partitioned between C_2_H_5_OH and H_2_O. The solution was stirred at room temperature for 30 min, and a solid was obtained by filtration while washing with H_2_O.

A white solid; Yield, 93.9%; m.p. 239.6–240.8 °C; ^1^H-NMR (400 MHz, Chloroform-*d*) δ 5.67 (s, 1H, CH-12), 3.70–3.53 (m, 8H, morpholine-H), 3.20 (dd, *J* = 10.8, 5.4 Hz, 1H, OH-3), 2.77 (dt, *J* = 13.4, 3.6 Hz, 1H, CH-1), 2.31 (s, 1H, CH-9), 2.27 (dd, *J* = 13.5, 3.7 Hz, 1H, CH-16), 1.34 (s, 3H, CH_3_-27), 1.20 (s, 3H, CH_3_-25), 1.11 (s, 3H, CH_3_-26), 1.10 (s, 3H, CH_3_-29), 0.98 (s, 3H, CH_3_-23), 0.87 (s, 3H, CH_3_-24), 0.78 (s, 3H, CH_3_-28), 0.68 (d, *J* = 11.4 Hz, 1H, CH-5); ^13^C-NMR (101 MHz, Chloroform-*d*) δ 200.12 (C11), 174.01 (C30), 169.45 (C13), 128.56 (C12), 78.75 (C3), 66.93 (morpholine C), 61.78 (C9), 54.92 (C5), 48.15 (C18), 45.26 (C14), 43.79 (C20), 43.70 (morpholine C), 43.26 (C8/19), 39.13 (C1), 39.10 (C4), 37.68 (C22), 37.06 (C10), 33.22 (C7), 32.79 (C17), 31.76 (C21), 28.39 (C29), 28.07 (C28), 27.27 (C23), 26.96 (C2), 26.70 (C15), 26.41 (C16), 23.14 (C27), 18.66 (C26), 17.46 (C6), 16.37 (C25), 15.56 (C24); HRMS (*m*/*z*): [M + H]^+^ calcd. for C_34_H_54_NO_4_: 540.40528, found: 540.40440.

*1H-benzo[d][1,2,3]triazol-1-yl-3β-hydroxy-11-oxo-olean-12-en-30-oate* (**2a**), white solid; Yield, 95.8%; m.p. 251.1–252.2 °C, (literature [43]: 192–195 °C, decomp.); ^1^H-NMR (400 MHz, Chloroform-*d*) δ 8.08 (d, *J* = 8.4 Hz, 1H, phenyl), 7.56 (t, *J* = 7.6 Hz, 1H, phenyl), 7.44 (t, *J* = 7.7 Hz, 1H, phenyl), 7.34 (d, *J* = 8.3 Hz, 1H, phenyl), 5.71 (s, 1H, CH-12), 3.22 (dd, *J* = 10.7, 5.5 Hz, 1H, OH-3), 2.77 (dt, *J* = 13.5, 3.6 Hz, 1H, CH-1), 2.39–2.23 (m, 2H, CH-9/16), 1.41 (s, 3H, CH_3_-27), 1.15 (s, 3H, CH_3_-25), 1.13 (s, 3H, CH_3_-26), 1.00 (s, 3H, CH_3_-29), 0.93 (s, 3H, CH_3_-23), 0.80 (s, 3H, CH_3_-24), 0.72 (d, *J* = 11.6 Hz, 1H, CH-5); ^13^C-NMR (101 MHz, Chloroform-*d*) δ 199.91 (C11), 172.51 (C30), 167.60 (C13), 143.54 (phenyl), 129.02 (phenyl), 128.83 (phenyl), 128.54 (C12), 124.82 (phenyl), 120.66, (phenyl) 107.81 (phenyl), 78.70 (C3), 61.83 (C9), 54.89 (C5), 48.20 (C18), 45.36 (C20), 44.36(C8), 43.15(C19), 40.85 (C1), 39.11 (C4), 37.75 (C22), 37.06 (C10), 32.72 (C7), 31.97 (C17), 31.16 (C21), 28.54 (C29), 28.08 (C28), 28.02 (C23), 27.25 (C2), 26.34 (C15/16), 23.48 (C27), 18.67 (C26), 17.45 (C6), 16.34 (C25), 15.57 (C24); HRMS (*m*/*z*): [M + H]^+^ calcd. for C_36_H_50_N_3_O_4_: 588.38013, found: 588.38018.

#### 3.2.2. General Procedure for Preparation of Carbamate Derivatives (**3a**–**3o**)

A mixture of 18β-GA **1** (0.19 g, 0.40 mmol) and substituted isocyanates (0.48 mmol) in ethyl acetate was stirred under reflux for 24 h. The organic layer was washed with 10% aqueous hydrochloric acid, 5% of aqueous NaHCO_3_, brine and was dried over anhydrous Na_2_SO_4_. The organic layer was then concentrated under reduced pressure. The residue obtained was purified by silica gel column chromatography using CH_2_Cl_2_–CH_3_OH as the eluent.

*3β-(((3,4-dichlorophenyl)carbamoyl)oxy)-11-oxo-olean-12-en-30-oic acid* (**3a**), white solid; Yield, 88.5%; m.p. 260.0–261.4 °C; ^1^H-NMR (400 MHz, Chloroform-*d*) δ 7.63 (s, 1H, phenyl-H), 7.33 (d, *J* = 8.7 Hz, 1H, phenyl-H), 7.18 (d, *J* = 8.8 Hz, 1H, phenyl-H), 6.71 (s, 1H, N-H), 5.70 (s, 1H, CH-12), 4.50 (dd, *J* = 10.5, 5.9 Hz, 1H, CH-3), 2.87–2.77 (m, 1H, CH-1), 2.37 (s, 1H, CH-9), 2.18 (dd, *J* = 13.8, 4.1 Hz, 1H, CH-16), 1.37 (s, 3H, CH_3_-27), 1.22 (s, 3H, CH_3_-25), 1.16 (s, 3H, CH_3_-26), 1.12 (s, 3H, CH_3_-29), 0.93 (s, 3H, CH_3_-23), 0.87 (s, 3H, CH_3_-24), 0.82 (s, 3H, CH_3_-28),0.80 (d, 1H, CH-5); ^13^C-NMR (101 MHz, Chloroform-*d*) δ 200.33 (C11), 181.32 (C30), 169.61 (C13), 137.62 (phenyl), 132.79 (phenyl), 130.45 (phenyl), 128.38 (C12), 126.31 (phenyl), 120.80 (phenyl), 117.72 (phenyl), 88.24 (C3), 61.62 (C9), 55.04 (C5), 48.23 (C18), 45.44 (C14), 43.77 (C20), 43.20 (C8), 40.81 (C19), 38.70 (C1), 38.23 (C4), 37.67 (C22), 36.88 (C10), 32.64 (C7), 31.85 (C17), 30.88 (C21), 28.53 (C29), 28.44 (C28), 28.09 (C23), 26.44 (C2), 26.36 (C15), 23.85 (C16), 23.36 (C27), 18.65 (C26), 17.33 (C6), 16.81 (C25), 16.39 (C24); HRMS (*m*/*z*): [M + H]^+^ calcd. for C_37_H_50_Cl_2_NO_5_: 658.30660, found: 658.31669.

*3β-(((4-chloro-3-(trifluoromethyl)phenyl)carbamoyl)oxy)-11-oxo-olean-12-en-30-oic acid* (**3b**), white solid; Yield, 87.0%; m.p. 247.8–248.5 °C; ^1^H-NMR (400 MHz, Chloroform-*d*) δ 7.76 (s, 1H, phenyl-H), 7.54 (s, 1H, phenyl-H), 7.40 (d, *J* = 8.7 Hz, 1H, phenyl-H), 6.83 (s, 1H, N-H), 5.70 (s, 1H, CH-12), 4.56–4.47 (m, 1H, CH-3), 2.82 (d, *J* = 13.6 Hz, 1H, CH-1), 2.37 (s, 1H, CH-9), 2.17 (d, *J* = 11.9 Hz, 1H, CH-16), 1.37 (s, 3H, CH_3_-27), 1.22 (s, 3H, CH_3_-25), 1.16 (s, 3H, CH_3_-26), 1.12 (s, 3H, CH_3_-29), 0.94 (s, 3H, CH_3_-23), 0.88 (s, 3H, CH_3_-24), 0.82 (s, 3H, CH_3_-28),0.80 (d, 1H, CH-5); ^13^C-NMR (101 MHz, Chloroform-*d*) δ 200.30 (C11), 181.19 (C30), 169.61 (C13), 137.02 (phenyl), 131.94 (phenyl), 128.90 (phenyl), 128.58 (phenyl), 128.38 (C12), 125.71(CF_3_), 123.90 (CF_3_), 82.55 (C9), 61.63 (C9), 55.05 (C5), 48.23 (C18), 45.44 (C14), 43.76 (C20), 43.19 (C8), 40.81 (C19), 38.70 (C1), 38.22 (C4), 37.67 (C22), 36.87 (C10), 32.63 (C7), 31.85 (C17), 30.89 (C21), 28.52 (C29), 28.42 (C28), 28.08 (C23), 26.44 (C2), 26.36 (C15), 23.84 (C16), 23.35 (C27), 18.65 (C26), 17.33 (C6), 16.78 (C25), 16.39 (C24); HRMS (*m*/*z*): [M + H]^+^ calcd. for C_38_H_50_ClF_3_NO_5_: 692.33296, found: 692.33792.

*3β-(((3,5-dichlorophenyl)carbamoyl)oxy)-11-oxo-olean-12-en-30-oic acid* (**3c**), white solid; Yield, 90.5%; m.p. 236.1–237.7 °C; ^1^H-NMR (400 MHz, Chloroform-*d*) δ 7.35 (s, 2H, phenyl-H), 7.02 (t, *J* = 1.8 Hz, 1H, phenyl-H), 6.62 (s, 1H, N-H), 5.69 (s, 1H, CH-12), 4.50 (dd, *J* = 10.7, 5.7 Hz, 1H, CH-3), 2.82 (d, *J* = 13.2 Hz, 1H, CH-1), 2.37 (s, 1H, CH-9), 2.17 (d, *J* = 11.9 Hz, 1H, CH-16), 1.37 (s, 3H, CH_3_-27), 1.22 (s, 3H, CH_3_-25), 1.16 (s, 3H, CH_3_-26), 1.12 (s, 3H, CH_3_-29), 0.94 (s, 3H, CH_3_-23), 0.88 (s, 3H, CH_3_-24), 0.82 (s, 3H, CH_3_-28), 0.79–0.73 (m, 1H, CH-5); ^13^C-NMR (101 MHz, DMSO-*d*_6_) δ199.36 (C11), 178.12 (C30), 170.33 (C13), 153.76 (carbamoyl), 142.30 (phenyl), 134.55 (phenyl), 127.62 (C12), 121.83 (phenyl), 116.62 (phenyl), 81.34 (C3), 61.24 (C9), 56.24 (C5), 48.48 (C18), 45.29 (C14), 43.50 (C20), 43.39 (C8), 41.03 (C19), 38.32 (C1/4), 37.94 (C22), 36.91 (C10), 32.56 (C7), 31.96 (C17), 30.02 (C21), 28.82 (C29), 28.24 (C28), 28.09 (C23), 26.51 (C2), 26.20 (C15), 23.91 (C16), 23.44 (C27), 18.73 (C26), 17.34 (C6), 17.15 (C25), 16.65 (C24); HRMS (*m*/*z*): [M + H]^+^ calcd. for C_37_H_50_Cl_2_NO_5_: 658.30660, found: 658.31238.

*3β-(((4-chlorophenyl)carbamoyl)oxy)-11-oxo-olean-12-en-30-oic acid* (**3d**), white solid; Yield, 87.3%; m.p. 235.1–236.7 °C; ^1^H-NMR (400 MHz, Chloroform-*d*) δ 7.33 (d, *J* = 8.4 Hz, 2H, phenyl-H), 7.25 (d, *J* = 2.6 Hz, 2H, phenyl-H), 6.66 (s, 1H, N-H), 5.70 (s, 1H, CH-12), 4.58–4.43 (m, 1H, CH-3), 2.81 (dt, *J* = 13.5, 3.6 Hz, 1H, CH-1), 2.37 (s, 1H, CH-9), 2.23–2.13 (m, 1H, CH-16), 1.37 (s, 3H, CH_3_-27), 1.22 (s, 3H, CH_3_-25), 1.16 (s, 3H, CH_3_-26), 1.12 (s, 3H, CH_3_-29), 0.94 (s, 3H, CH_3_-23), 0.87 (s, 3H, CH_3_-24), 0.82 (s, 3H, CH_3_-28), 0.80 (s, 1H, CH-5); ^13^C-NMR (101 MHz, Chloroform-*d*) δ 200.35 (C11), 181.38 (C30), 169.57 (C13), 136.66 (phenyl), 128.97 (phenyl), 128.38 (C12), 128.15 (phenyl), 119.68 (phenyl), 81.95 (C3), 61.64 (C9), 55.04 (C5), 48.22 (C18), 45.44 (C14), 43.77 (C20), 43.19 (C8), 40.81 (C19), 38.72 (C1), 38.24 (C4), 37.68 (C22), 36.88 (C10), 32.65 (C7), 31.85 (C17), 30.88 (C21), 28.53 (C29), 28.44 (C28), 28.08 (C23), 26.44 (C2), 26.36 (C15), 23.88 (C16), 23.35 (C27), 18.65 (C26), 17.34 (C6), 16.82 (C25), 16.40 (C24); HRMS (*m*/*z*): [M + H]^+^ calcd. for C_37_H_51_ClNO_5_: 624.34558, found: 624.35065.

*3β-(((3-chlorophenyl)carbamoyl)oxy)-11-oxo-olean-12-en-30-oic acid* (**3e**), white solid; Yield, 89.9%; m.p. 268.0.0–269.5 °C; ^1^H-NMR (400 MHz, Chloroform-*d*) δ 7.51 (s, 1H, phenyl-H), 7.20 (d, *J* = 4.9 Hz, 2H, phenyl-H), 7.00 (td, *J* = 4.5, 2.0 Hz, 1H, phenyl-H), 6.69 (s, 1H, N-H), 5.70 (s, 1H, CH-12), 4.50 (dd, *J* = 10.4, 6.1 Hz, 1H, CH-3), 2.82 (dt, *J* = 13.6, 3.6 Hz, 1H, CH-1), 2.37 (s, 1H, CH-9), 2.18 (dd, *J* = 13.0, 3.7 Hz, 1H, CH-16), 1.37 (s, 3H, CH_3_-27), 1.22 (s, 3H, CH_3_-25), 1.16 (s, 3H, CH_3_-26), 1.12 (s, 3H, CH_3_-29), 0.94 (s, 3H, CH_3_-23), 0.88 (s, 3H, CH_3_-24), 0.82 (s, 3H, CH_3_-28), 0.82-0.81 (m, 1H, CH-5); ^13^C-NMR (101 MHz, Chloroform-*d*) δ 200.34 (C11), 181.33 (C30), 169.56 (C13), 153.35 (carbamoyl), 139.25 (phenyl), 134.70 (phenyl), 129.94 (phenyl), 128.39 (C12), 123.22 (phenyl), 118.52 (phenyl),116.46 (phenyl), 82.11 (C3), 61.64 (C9), 55.04 (C5), 48.22 (C18), 45.44 (C14), 43.77 (C20), 43.19 (C8), 40.81 (C19), 38.72 (C1), 38.24 (C4), 37.68 (C22), 36.88 (C10), 32.65 (C7), 31.85 (C17), 30.88 (C21), 28.53 (C29), 28.44 (C28), 28.08 (C23), 26.44 (C2), 26.36 (C15), 23.86 (C16), 23.36 (C27), 18.65 (C26), 17.34 (C6), 16.82 (C25), 16.39 (C24); HRMS (*m*/*z*): [M + H]^+^ calcd. for C_37_H_51_ClNO_5_: 624.34558, found: 624.34960.

*3β-(((3-chloro-4-methylphenyl)carbamoyl)oxy)-11-oxo-olean-12-en-30-oic acid* (**3f**), white solid; Yield, 90.4%; m.p. 270.7–271.6 °C; ^1^H-NMR (400 MHz, Chloroform-*d*) δ 7.48 (s, 1H, phenyl-H), 7.11 (s, 2H, phenyl-H), 6.60 (s, 1H, N-H), 5.70 (s, 1H, CH-12), 4.49 (t, *J* = 8.3 Hz, 1H, CH-3), 2.81 (dt, *J* = 13.4, 3.6 Hz, 1H, CH-1), 2.37 (s, 1H, CH-9), 2.29 (s, 3H, phenyl-CH_3_), 2.18 (dd, *J* = 13.3, 4.2 Hz, 1H, CH-16), 1.37 (s, 3H, CH_3_-27), 1.22 (s, 3H, CH_3_-25), 1.16 (s, 3H, CH_3_-26), 1.12 (s, 3H, CH_3_-29), 0.94 (s, 3H, CH_3_-23), 0.88 (s, 3H, CH_3_-24), 0.82 (s, 3H, CH_3_-28), 0.80 (m, 1H, CH-5); ^13^C-NMR (101 MHz, Chloroform-*d*) δ 200.36 (C11), 181.39 (C30), 169.56 (C13), 153.48 (carbamoyl), 136.83 (phenyl), 134.49 (phenyl), 130.95 (phenyl), 130.62 (phenyl), 128.39 (C12), 119.22 (phenyl), 116.83 (phenyl), 81.85 (C3), 61.64 (C9), 55.05 (C5), 48.22 (C18), 45.45 (C14), 43.77 (C20), 43.19 (C8), 40.80 (C19), 38.73 (C1), 38.24 (C4), 37.68 (C22), 36.89 (C10), 32.65 (C7), 31.85 (C17), 30.88 (C21), 28.53 (C29), 28.44 (C28), 28.08 (C23), 26.44 (C2), 26.37 (C15), 23.87 (C16), 23.36 (C27), 19.33 (phenyl-CH_3_), 18.65 (C26), 17.34 (C6), 16.82 (C25), 16.39 (C24); HRMS (*m*/*z*): [M + Na]^+^ calcd. for C_38_H_52_ClNNaO_5_: 660.34317, found: 660.34747.

*3β-(((4-bromophenyl)carbamoyl)oxy)-11-oxo-olean-12-en-30-oic acid* (**3g**), white solid; Yield, 87.2%; m.p. 263.0–265.0 °C; ^1^H-NMR (400 MHz, Chloroform-*d*) δ 7.42–7.35 (m, 2H, phenyl-H), 7.27 (d, *J* = 8.4 Hz, 2H, phenyl-H), 6.65 (s, 1H, N-H), 5.70 (s, 1H, CH-12), 4.50 (t, *J* = 8.3 Hz, 1H, CH-3), 2.85–2.77 (m, 1H, CH-1), 2.37 (s, 1H, CH-9), 2.18 (dd, *J* = 13.7, 4.1 Hz, 1H, CH-16), 1.37 (s, 3H, CH_3_-27), 1.21 (s, 3H, CH_3_-25), 1.16 (s, 3H, CH_3_-26), 1.12 (s, 3H, CH_3_-29), 0.94 (s, 3H, CH_3_-23), 0.87 (s, 3H, CH_3_-24), 0.82 (s, 3H, CH_3_-28), 0.80 (s, 1H, CH-5); ^13^C-NMR (101 MHz, Chloroform-*d*) δ 200.34 (C11), 181.30 (C30), 169.56 (C13), 153.36 (carbamoyl), 137.17 (phenyl), 131.91 (phenyl), 128.39 (C12), 120.05 (phenyl), 81.92 (C3), 61.64 (C9), 55.04 (C5), 48.22 (C18), 45.44 (C14), 43.77 (C20), 43.19 (C8), 40.81 (C19), 38.72 (C1), 38.24 (C4), 37.68 (C22), 36.88 (C10), 32.65 (C7), 31.85 (C17), 30.89 (C21), 28.52 (C29), 28.43 (C28), 28.08 (C23), 26.44 (C2), 26.36 (C15), 23.87 (C16), 23.35 (C27), 18.65 (C26), 17.34 (C6), 16.82 (C25), 16.40 (C24); HRMS (*m*/*z*): [M + H]^+^ calcd. for C_37_H_51_BrNO_5_: 668.29506, found: 668.30334, 670.30211.

*3β-(((4-fluorophenyl)carbamoyl)oxy)-11-oxo-olean-12-en-30-oic acid* (**3h**), white solid; Yield, 88.6%; m.p. 267.9–268.9 °C; ^1^H-NMR (400 MHz, DMSO-*d*_6_) δ 12.25 (s, 1H, COOH), 9.57 (s, 1H, N-H), 7.51 (s, 2H, phenyl-H), 7.14 (t, *J* = 8.9 Hz, 2H, phenyl-H), 5.44 (s, 1H, CH-12), 4.42 (dd, *J* = 11.8, 4.6 Hz, 1H, CH-3), 2.73–2.65 (m, 1H, CH-1), 2.46 (s, 1H, CH-9), 2.18–2.04 (m, 2H, CH-16, CH-2), 1.41 (s, 3H, CH_3_-27), 1.13 (s, 3H, CH_3_-25), 1.11 (s, 3H, CH_3_-26), 1.08 (s, 3H, CH_3_-29), 0.92 (s, 3H, CH_3_-23), 0.91 (s, 3H, CH_3_-24), 0.79 (s, 3H, CH_3_-28); ^13^C-NMR (101 MHz, Chloroform-*d*) δ 200.36 (C11), 181.39 (C30), 169.55 (C13), 153.81 (carbamoyl), 134.01 (phenyl), 128.39 (C12), 120.13 (phenyl), 115.59 (d, *J* = 22 Hz, phenyl), 81.63 (C3), 61.65 (C9), 55.04 (C5), 48.22 (C18), 45.45 (C14), 43.77 (C20), 43.19 (C8), 40.81 (C19), 38.73 (C1), 38.25 (C4), 37.68 (C22), 36.88 (C10), 32.66 (C7), 31.85 (C17), 30.88 (C21), 28.53 (C29), 28.44 (C28), 28.07 (C23), 26.44 (C2), 26.36 (C15), 23.89 (C16), 23.35 (C27), 18.65 (C26), 17.34 (C6), 16.81 (C25), 16.40 (C24); HRMS (*m*/*z*): [M + H]^+^ calcd. for C_37_H_51_FNO_5_: 608.37513, found: 608.38190.

*3β-(((4-(trifluoromethyl)phenyl)carbamoyl)oxy)-11-oxo-olean-12-en-30-oic acid* (**3i**), white solid; Yield, 90.8%; m.p. 242.1–242.9 °C; ^1^H-NMR (400 MHz, Chloroform-*d*) δ 7.52 (q, *J* = 8.7 Hz, 4H, phenyl-H), 6.87 (s, 1H, N-H), 5.71 (s, 1H, CH-12), 4.52 (dd, *J* = 10.5, 5.8 Hz, 1H, CH-3), 2.82 (dt, *J* = 13.6, 3.6 Hz, 1H, CH-1), 2.37 (s, 1H, CH-9), 2.18 (dd, *J* = 13.5, 4.1 Hz, 1H, CH-16), 1.37 (s, 3H, CH_3_-27), 1.22 (s, 3H, CH_3_-25), 1.16 (s, 3H, CH_3_-26), 1.12 (s, 3H, CH_3_-29), 0.95 (s, 3H, CH_3_-23), 0.89 (s, 3H, CH_3_-24), 0.82 (s, 3H, CH_3_-28), 0.81 (s, 1H, CH-5); ^13^C-NMR (101 MHz, Chloroform-*d*) δ 200.35 (C11), 181.40 (C30), 169.64 (C13), 153.37 (carbamoyl), 141.21 (phenyl), 128.37 (C12), 126.29 (q, *J* = 3.8 Hz, CF_3_), 126.27 (CF_3_), 126.23 (CF_3_), 125.50 (phenyl), 125.13 (phenyl), 124.80 (phenyl), 122.80 (phenyl), 117.97 (phenyl), 82.26 (C3), 61.64 (C9), 55.05 (C5), 48.23 (C18), 45.45 (C14), 43.78 (C20), 43.20 (C8), 40.81 (C19), 38.72 (C1), 38.24 (C4), 37.68 (C22), 36.88 (C10), 32.64 (C7), 31.85 (C17), 30.88 (C21), 28.53 (C29), 28.44 (C28), 28.08 (C23), 26.44 (C2), 26.36 (C15), 23.86 (C16), 23.35 (C27), 18.65 (C26), 17.33 (C6), 16.83 (C25), 16.40 (C24); HRMS (*m*/*z*): [M + H]^+^ calcd. for C_38_H_51_F_3_NO_5_: 658.37193, found: 658.37843.

*3β-(((3-(trifluoromethyl)phenyl)carbamoyl)oxy)-11-oxo-olean-12-en-30-oic acid* (**3j**), white solid; Yield, 89.2%; m.p. 239.1–240.7 °C; ^1^H-NMR (400 MHz, Chloroform-*d*) δ 7.72 (s, 1H, phenyl-H), 7.40 (t, *J* = 8.0 Hz, 1H, phenyl-H), 7.28 (d, *J* = 7.8 Hz, 1H, phenyl-H), 6.82 (s, 1H, N-H), 5.71 (s, 1H, CH-12), 4.52 (t, *J* = 8.3 Hz, 1H, CH-3), 2.86–2.78 (m, 1H, CH-1), 2.37 (s, 1H, CH-9), 2.23–2.13 (m, 1H, CH-16), 1.37 (s, 3H, CH_3_-27), 1.22 (s, 3H, CH_3_-25), 1.16 (s, 3H, CH_3_-26), 1.12 (s, 3H, CH_3_-29), 0.95 (s, 3H, CH_3_-23), 0.89 (s, 3H, CH_3_-24), 0.82 (s, 3H, CH_3_-28), 0.81 (s, 1H, CH-5); ^13^C-NMR (101 MHz, Chloroform-*d*) δ 200.32 (C11), 181.25 (C30), 169.57 (C13), 153.42 (carbamoyl), 138.63 (phenyl), 131.56 (CF_3_), 131.24 (CF_3_), 130.91 (CF_3_), 129.50 (phenyl), 128.39 (C12), 125.21 (phenyl), 122.50 (phenyl), 119.76 (phenyl), 82.36 (C3), 61.64 (C9), 55.06 (C5), 48.23 (C18), 45.44 (C14), 43.77 (C20), 43.19 (C8), 40.81 (C19), 38.72 (C1), 38.22 (C4), 37.68 (C22), 36.88 (C10), 32.64 (C7), 31.85 (C17), 30.89 (C21), 28.52 (C29), 28.43 (C28), 28.09 (C23), 26.44 (C2), 26.36 (C15), 23.85 (C16), 23.36 (C27), 18.65 (C26), 17.34 (C6), 16.79 (C25), 16.39 (C24); HRMS (*m*/*z*): [M + H]^+^ calcd. for C_38_H_51_F_3_NO_5_: 658.37193, found: 658.37843.

*3β-(((3,5-bis (trifluoromethyl)phenyl)carbamoyl)oxy)-11-oxo-olean-12-en-30-oic acid* (**3k**), white solid; Yield, 87.0%; m.p. 248.8–251.7 °C; ^1^H-NMR (400 MHz, Chloroform-*d*) δ 7.90 (s, 2H, phenyl-H), 7.53 (s, 1H, phenyl-H), 6.97 (s, 1H, N-H), 5.70 (s, 1H, CH-12), 4.53 (dd, *J* = 10.6, 5.9 Hz, 1H, CH-3), 2.84 (dd, *J* = 10.2, 3.4 Hz, 1H, CH-1), 2.37 (s, 1H, CH-9), 2.22–2.13 (m, 1H, CH-16), 1.37 (s, 3H, CH_3_-27), 1.22 (s, 3H, CH_3_-25), 1.16 (s, 3H, CH_3_-26), 1.13 (s, 3H, CH_3_-29), 0.95 (s, 3H, CH_3_-23), 0.89 (s, 3H, CH_3_-24), 0.82 (s, 3H, CH_3_-28), 0.81 (s, 1H, CH-5); ^13^C-NMR (101 MHz, Chloroform-*d*) δ 200.21 (C11), 180.40 (C30), 169.51 (C13), 153.11 (carbamoyl), 139.60 (phenyl), 132.52 (phenyl), 132.19 (phenyl), 128.41 (C12), 124.43 (CF_3_), 121.72 (CF_3_), 118.07 (phenyl), 116.43 (phenyl), 82.59 (C3), 61.61 (C9), 55.06 (C5), 48.23 (C18), 45.42 (C14), 43.72 (C20), 43.20 (C8), 40.85 (C19), 38.69 (C1), 38.20 (C4), 37.66 (C22), 36.87 (C10), 32.63 (C7), 31.85 (C17), 30.92 (C21), 28.51 (C29), 28.39 (C28), 28.10 (C23), 26.44 (C2), 26.36 (C15), 23.81(C16), 23.36 (C27), 18.65 (C26), 17.33 (C6), 16.77(C25), 16.38 (C24); HRMS (*m*/*z*): [M + H]^+^ calcd. for C_39_H_50_F_6_NO_5_: 726.35932, found: 726.36406.

*3β-(((3-methoxyphenyl)carbamoyl)oxy)-11-oxo-olean-12-en-30-oic acid* (**3l**), white solid; Yield, 86.9%; m.p. 259.5–261.1°C; ^1^H-NMR (400 MHz, Chloroform-*d*) δ 7.17 (t, *J* = 8.2 Hz, 1H, phenyl-H), 6.84 (d, *J* = 8.0 Hz, 1H, phenyl-H), 6.65 (s, 1H, N-H), 6.59 (dd, *J* = 8.4, 2.4 Hz, 1H, phenyl-H), 5.70 (s, 1H, CH-12), 4.55–4.45 (m, 1H, CH-3), 3.79 (s, 3H, CH_3_), 2.85–2.76 (m, 1H, CH-1), 2.37 (s, 1H, CH-9), 2.18 (dd, *J* = 13.4, 4.1 Hz, 1H, CH-16), 1.37 (s, 3H, CH_3_-27), 1.21 (s, 3H, CH_3_-25), 1.16 (s, 3H, CH_3_-26), 1.12 (s, 3H, CH_3_-29), 0.94 (s, 3H, CH_3_-23), 0.88 (s, 3H, CH_3_-24), 0.82 (s, 3H, CH_3_-28), 0.80 (s, 1H, CH-5); ^13^C-NMR (101 MHz, Chloroform-*d*) δ 200.37 (C11), 181.35 (C30), 169.53 (C13), 160.22 (phenyl), 153.37 (carbamoyl), 139.31 (phenyl), 129.67(phenyl), 128.39 (C12), 109.15 (phenyl), 81.72 (C3), 61.66 (C9), 55.25 (-OCH_3_), 55.08 (C5), 48.23 (C18), 45.45 (C14), 43.77 (C20), 43.18 (C8), 40.81 (C19), 38.76 (C1), 38.25 (C4), 37.68 (C22), 36.89 (C10), 32.67 (C7), 31.85 (C17), 30.88 (C21), 28.53 (C29), 28.44 (C28), 28.07 (C23), 26.44 (C2), 26.37 (C15), 23.89 (C16), 23.34 (C27), 18.66 (C26), 17.34 (C6), 16.83 (C25), 16.40 (C24); HRMS (*m*/*z*): [M + Na]^+^ calcd. for C_38_H_53_NNaO_6_: 642.37706, found: 642.37890.

*3β-(((4-methoxyphenyl)carbamoyl)oxy)-11-oxo-olean-12-en-30-oic acid* (**3m**), white solid; Yield, 89.3%; m.p. 264.0–264.8 °C; ^1^H-NMR (400 MHz, Chloroform-*d*) δ 7.28 (s, 2H, phenyl-H), 6.83 (d, *J* = 8.9 Hz, 2H, phenyl-H), 6.50 (s, 1H, N-H), 5.70 (s, 1H, CH-12),4.48 (t, *J* = 8.2 Hz, 1H, CH-3), 3.76 (s, 3H, CH_3_), 2.80 (dt, *J* = 13.5, 3.7 Hz, 1H, CH-1), 2.37 (s, 1H, CH-9), 2.22–2.13 (m, 1H, CH-16), 1.36 (s, 3H, CH_3_-27), 1.21 (s, 3H, CH_3_-25), 1.15 (s, 3H, CH_3_-26), 1.11 (s, 3H, CH_3_-29), 0.93 (s, 3H, CH_3_-23), 0.86 (s, 3H, CH_3_-24), 0.82 (s, 3H, CH_3_-28), 0.80 (s, 1H, CH-5); ^13^C-NMR (101 MHz, Chloroform-*d*) δ 200.39 (C11), 181.40 (C30), 169.53 (C13), 153.85 (carbamoyl), 131.13 (phenyl), 128.39 (C12), 120.33 (phenyl), 114.16 (phenyl), 81.45 (C3), 61.66 (C9), 55.48 (-OCH_3_), 55.04 (C5), 48.21 (C18), 45.45 (C14), 43.77 (C20), 43.18 (C8), 40.81 (C19), 38.74 (C1), 38.26 (C4), 37.69 (C22), 36.89 (C10), 32.67 (C7), 31.85 (C17), 30.89 (C21), 28.52 (C29), 28.44 (C28), 28.07 (C23), 26.44 (C2), 26.37 (C15), 23.91 (C16), 23.35 (C27), 18.65 (C26), 17.34 (C6), 16.81 (C25), 16.39 (C24); HRMS (*m*/*z*): [M + Na]^+^ calcd. for C_38_H_53_NNaO_6_: 642.37706, found: 642.38301.

*3β-(((4-(trifluoromethoxy)phenyl)carbamoyl)oxy)-11-oxo-olean-12-en-30-oic acid* (**3n**), white solid; Yield, 92.1%; m.p. 256.4–258.4°C; ^1^H-NMR (400 MHz, Chloroform-*d*) δ 7.40 (d, *J* = 8.5 Hz, 2H, phenyl-H), 7.14 (d, *J* = 8.6 Hz, 2H, phenyl-H), 6.72 (s, 1H, N-H), 5.70 (s, 1H, CH-12), 4.58–4.42 (m, 1H, CH-3), 2.85–2.76 (m, 1H, CH-1), 2.37 (s, 1H, CH-9), 2.18 (dd, *J* = 13.5, 4.1 Hz, 1H, CH-16), 1.36 (s, 3H, CH_3_-27), 1.21 (s, 3H, CH_3_-25), 1.16 (s, 3H, CH_3_-26), 1.12 (s, 3H, CH_3_-29), 0.94 (s, 3H, CH_3_-23), 0.88 (s, 3H, CH_3_-24), 0.82 (s, 3H, CH_3_-28), 0.80 (s, 1H, CH-5); ^13^C-NMR (101 MHz, Chloroform-*d*) δ 200.36 (C11), 181.49 (C30), 169.62 (C13), 153.29 (carbamoyl), 144.53(phenyl), 136.79 (phenyl), 128.37 (C12), 124.28 (CF_3_), 121.84 (phenyl), 121.73 (phenyl), 119.47 (phenyl), 119.18 (phenyl), 81.99 (C3), 61.64 (C9), 55.04 (C5), 48.22 (C18), 45.44 (C14), 43.78 (C20), 43.19 (C8), 40.80 (C19), 38.72 (C1), 38.25 (C4), 37.68 (C22), 36.88 (C10), 32.65 (C7), 31.85 (C17), 30.88 (C21), 28.52 (C29), 28.44 (C28), 28.06 (C23), 26.44 (C2), 26.36 (C15), 23.87 (C16), 23.35 (C27), 18.65 (C26), 17.33 (C6), 16.81(C25), 16.39 (C24); HRMS (*m*/*z*): [M + H]^+^ calcd. for C_38_H_51_F_3_NO_6_: 674.36685, found: 674.37311.

*3β-(((3,5-dimethylphenyl)carbamoyl)oxy)-11-oxo-olean-12-en-30-oic acid* (**3o**), white solid; Yield, 92.1%; m.p. 279.8–280.6 °C; ^1^H-NMR (400 MHz, Chloroform-*d*) δ 7.01 (s, 2H, phenyl-H), 6.68 (s, 1H, phenyl-H), 6.55 (s, 1H, N-H), 5.71 (s, 1H, CH-12), 4.49 (t, *J* = 8.2 Hz, 1H, CH-3), 2.85–2.76 (m, 1H, CH-1), 2.37 (s, 1H, CH-9), 2.27 (s, 6H, phenyl-CH_3_), 2.18 (dd, *J* = 13.5, 4.1 Hz, 1H, CH-16), 1.37 (s, 3H, CH_3_-27), 1.22 (s, 3H, CH_3_-25), 1.16 (s, 3H, CH_3_-26), 1.12 (s, 3H, CH_3_-29), 0.94 (s, 3H, CH_3_-23), 0.88 (s, 3H, CH_3_-24), 0.82 (s, 3H, CH_3_-28), 0.80 (s, 1H, CH-5); ^13^C-NMR (101 MHz, Chloroform-*d*) δ 200.38 (C11), 181.43 (C30), 169.53 (C13), 153.61 (carbamoyl), 138.68 (phenyl), 137.84 (phenyl), 128.40 (C12), 124.95 (phenyl), 119.26 (phenyl), 81.69 (C3), 61.65 (C9), 55.07 (C5), 48.22 (C18), 45.45 (C14), 43.77 (C20), 43.19 (C8), 40.81 (C19), 38.75 (C1), 38.24 (C4), 37.69 (C22), 36.90 (C10), 32.67 (C7), 31.85 (C17), 30.89 (C21), 28.53 (C29), 28.44 (C28), 28.08 (C23), 26.44 (C2), 26.37 (C15), 23.90 (C16), 23.37 (C27), 21.37 (phenyl-CH_3_), 21.34 (phenyl-CH_3_), 18.66 (C26), 17.35 (C6), 16.82 (C25), 16.39 (C24); HRMS (*m*/*z*): [M + Na]^+^ calcd. for C_39_H_55_NNaO_5_: 640.39779, found: 640.340185.

#### 3.2.3. General Procedure for Preparation of Carbamate Derivatives (**4a**–**4o**)

A mixture of compound **2** (0.22 g, 0.40 mmol) and substituted isocyanates (0.48 mmol) in ethyl acetate was stirred under reflux for 24 h. The organic layer was washed with 10% aqueous hydrochloric acid, 5% of aqueous NaHCO_3_, brine and was dried over anhydrous Na_2_SO_4_. The organic layer was then concentrated under reduced pressure. The residue obtained was purified by silica gel column chromatography using CH_2_Cl_2_–CH_3_OH as the eluent.

*3β-(((3,4-dichlorophenyl)carbamoyl)oxy)-30-morpholino-olean-12-ene-11,30-dione* (**4a**), white solid; Yield, 93.0%; m.p.292.5–293.0 °C; ^1^H-NMR (400 MHz, Chloroform-*d*) δ 7.63 (s, 1H, phenyl-H), 7.32 (d, *J* = 8.8 Hz, 1H, phenyl-H), 7.18 (d, *J* = 8.8 Hz, 1H, phenyl-H), 6.72 (s, 1H, N-H), 5.68 (s, 1H, CH-12), 4.48 (dd, *J* = 10.8, 5.7 Hz, 1H, CH-3), 3.65 (t, *J* = 6.3 Hz, 8H, morpholine-H), 2.81 (dt, *J* = 13.4, 3.8 Hz, 1H, CH-1), 2.35 (s, 1H, CH-9), 2.27 (d, *J* = 12.7 Hz, 1H, CH-16), 1.35 (s, 3H, CH_3_-27), 1.20 (s, 3H, CH_3_-25), 1.14 (s, 3H, CH_3_-26), 1.10 (s, 3H, CH_3_-29), 0.93 (s, 3H, CH_3_-23), 0.86 (s, 3H, CH_3_-24), 0.80 (s, 3H, CH_3_-28), 0.79 (s, 1H, CH-5); ^13^C-NMR (101 MHz, Chloroform-*d*) δ 199.99 (C11), 174.02 (C30), 169.71 (C13), 153.23 (carbamoyl), 137.66 (phenyl), 132.78 (phenyl), 130.44 (phenyl), 128.46 (C12), 126.27 (phenyl), 120.13 (phenyl), 117.70 (phenyl), 82.25 (C3), 66.93 (morpholine C), 61.64 (C9), 55.05 (C5), 48.20 (C18), 45.28 (C14), 43.79 (C20), 43.66 (morpholine C), 43.29 (C8), 38.74 (C1), 38.22 (C4), 37.67 (C22), 36.89 (C10), 33.23 (morpholine C), 32.69 (C7), 31.77 (C17), 28.41 (C28), 28.08 (C23), 26.96 (C2), 26.68 (C15), 26.40 (C16), 23.84 (C29), 23.11(C27), 18.66 (C26), 17.34 (C6), 16.79 (C25), 16.41 (C24); HRMS (*m*/*z*): [M + Na]^+^ calcd. for C_41_H_56_Cl_2_N_2_NaO_5_: 749.34640, found: 749.34901.

*3β-(((4-chloro-3-(trifluoromethyl)phenyl)carbamoyl)oxy)-30-morpholino-olean-12-ene-11,30-dione* (**4b**), white solid; Yield, 92.5%; m.p. 209.5–211.1 °C; ^1^H-NMR (400 MHz, Chloroform-*d*) δ 7.77 (d, *J* = 2.5 Hz, 1H, phenyl-H), 7.54 (d, *J* = 8.6 Hz, 1H, phenyl-H), 7.40 (d, *J* = 8.7 Hz, 1H, phenyl-H), 6.85 (s, 1H, N-H), 5.68 (s, 1H, CH-12), 4.50 (dd, *J* = 11.0, 5.5 Hz, 1H, CH-3), 3.70–3.56 (m, 8H, morpholine-H), 2.81 (dt, *J* = 13.7, 3.7 Hz, 1H, CH-1), 2.35 (s, 1H, CH-9), 2.32–2.23 (m, 1H, CH-16), 1.35 (s, 3H, CH_3_-27), 1.21 (s, 3H, CH_3_-25), 1.15 (s, 3H, CH_3_-26), 1.11 (s, 3H, CH_3_-29), 0.93 (s, 3H, CH_3_-23), 0.87 (s, 3H, CH_3_-24), 0.82 (s, 3H, CH_3_-28), 0.80 (s, 1H, CH-5); ^13^C-NMR (101 MHz, Chloroform-*d*) δ 199.97 (C11), 174.04 (C30), 169.70 (C13), 153.30 (carbamoyl), 137.09 (phenyl), 131.94 (phenyl), 128.90 (phenyl), 128.58 (phenyl), 128.48 (C12), 125.29 (q, *J* = 271 Hz, CF_3_), 121.21 (phenyl), 117.45 (phenyl), 82.43 (C3), 66.95 (morpholine C), 61.65 (C9), 55.08 (C5), 48.21 (C18), 45.29 (C14), 43.80 (C20), 43.68 (morpholine C), 43.30 (C8), 38.75 (C1), 38.22 (C4), 37.69 (C22), 36.90 (C10), 33.24 (morpholine C), 32.71 (C7), 31.78 (C17), 28.42 (C28), 28.10 (C23), 26.97 (C2), 26.70 (C15), 26.41 (C16), 23.85 (C29), 23.11(C27), 18.67(C26), 17.35 (C6), 16.79 (C25), 16.42 (C24); HRMS (*m*/*z*): [M + Na]^+^ calcd. for C_42_H_56_ClF_3_N_2_NaO_5_: 783.37275, found: 783.37677.

*3β-(((3,5-dichlorophenyl)carbamoyl)oxy)-30-morpholino-olean-12-ene-11,30-dione* (**4c**), white solid; Yield, 91.7%; m.p. 278.2–280.3 °C; ^1^H-NMR (400 MHz, Chloroform-*d*) δ 7.35 (s, 2H, phenyl-H), 7.01 (q, *J* = 2.7, 1.9 Hz, 1H, phenyl-H), 6.78 (s, 1H, N-H), 5.68 (s, 1H, CH-12), 4.48 (dd, *J* = 10.9, 5.5 Hz, 1H, CH-3), 3.65 (q, *J* = 6.4, 5.8 Hz, 8H, morpholine-H), 2.81 (dt, *J* = 13.4, 3.4 Hz, 1H, CH-1), 2.35 (s, 1H, CH-9), 2.31–2.22 (m, 1H, CH-16), 1.35 (s, 3H, CH_3_-27), 1.21 (s, 3H, CH_3_-25), 1.14 (s, 3H, CH_3_-26), 1.11 (s, 3H, CH_3_-29), 0.93 (s, 3H, CH_3_-23), 0.87 (s, 3H, CH_3_-24), 0.82(s, 1H, CH-5), 0.80 (s, 3H, CH_3_-28); ^13^C-NMR (101 MHz, Chloroform-*d*) δ 199.97 (C11), 174.03 (C30), 169.69 (C13), 153.08 (carbamoyl), 140.05 (phenyl), 135.23 (phenyl), 128.47 (C12), 123.06 (phenyl), 116.67 (phenyl), 82.38 (C3), 66.94 (morpholine C), 61.64 (C9), 55.05 (C5), 48.21 (C18), 45.28 (C14), 43.79 (C20), 43.65 (morpholine C), 43.29 (C8), 38.73 (C1), 38.21 (C4), 37.67 (C22), 36.89 (C10), 33.25 (morpholine C), 32.69 (C7), 31.77 (C17), 28.41 (C28), 28.08 (C23), 26.96(C2), 26.68 (C15), 26.40 (C16), 23.82 (C29), 23.12 (C27), 18.66 (C26), 17.34 (C6), 16.79 (C25), 16.41 (C24); HRMS (*m*/*z*): [M + Na]^+^ calcd. for C_41_H_56_Cl_2_N_2_NaO_5_: 749.34640, found: 749.34951.

*3β-(((4-chlorophenyl)carbamoyl)oxy)-30-morpholino-olean-12-ene-11,30-dione* (**4d**), white solid; Yield, 92.8%; m.p. 298.4–299.7 °C; ^1^H-NMR (400 MHz, Chloroform-*d*) δ 7.33 (d, *J* = 8.6 Hz, 2H, phenyl-H), 7.24 (d, *J* = 7.8 Hz, 2H, phenyl-H), 6.64 (s, 1H, N-H), 5.68 (s, 1H, CH-12), 4.48 (dd, *J* = 10.6, 5.9 Hz, 1H, CH-3), 3.67-3.56 (m, 8H, morpholine-H), 2.80 (dt, *J* = 13.6, 3.6 Hz, 1H, CH-1), 2.35 (s, 1H, CH-9), 2.31–2.22 (m, 1H, CH-16), 1.35 (s, 3H, CH_3_-27), 1.20 (s, 3H, CH_3_-25), 1.15 (s, 3H, CH_3_-26), 1.11 (s, 3H, CH_3_-29), 0.93 (s, 3H, CH_3_-23), 0.87 (s, 3H, CH_3_-24), 0.82(s, 1H, CH-5), 0.80 (s, 3H, CH_3_-28); ^13^C-NMR (101 MHz, Chloroform-*d*) δ 199.98 (C11), 174.01 (C30), 169.61 (C13), 153.46 (carbamoyl), 136.69 (phenyl), 128.97 (phenyl), 128.48 (C12), 128.13 (phenyl), 119.68 (phenyl), 81.88 (C3), 66.94 (morpholine C), 61.66 (C9), 55.06 (C5), 48.21 (C18), 45.28 (C14), 43.78 (C20), 43.64 (morpholine C), 43.28 (C8), 38.76 (C1), 38.23 (C4), 37.67 (C22), 36.89 (C10), 33.27 (morpholine C), 32.71 (C7), 31.77 (C17), 28.41 (C28), 28.07 (C23), 26.96 (C2), 26.69 (C15), 26.40 (C16), 23.87 (C29), 23.11 (C27), 18.66 (C26), 17.35 (C6), 16.80 (C25), 16.41 (C24); HRMS (*m*/*z*): [M + Na]^+^ calcd. for C_41_H_57_ClN_2_NaO_5_: 715.38537, found: 715.38855.

*3β-(((3-chlorophenyl)carbamoyl)oxy)-30-morpholino-olean-12-ene-11,30-dione* (**4e**), white solid; Yield, 94.4%; m.p. 291.0–292.5 °C; ^1^H-NMR (400 MHz, Chloroform-*d*) δ 7.52 (s, 1H, phenyl-H), 7.19 (d, *J* = 5.1 Hz, 2H, phenyl-H), 7.00 (dq, *J* = 7.1, 2.0 Hz, 1H, phenyl-H), 6.70 (s, 1H, N-H), 5.68 (s, 1H, CH-12), 4.49 (dd, *J* = 10.7, 5.9 Hz, 1H, CH-3), 3.69–3.56 (m, 8H, morpholine-H), 2.80 (dt, *J* = 13.6, 3.6 Hz, 1H, CH-1), 2.35 (s, 1H, CH-9), 2.27 (d, *J* = 13.2 Hz, 1H, CH-16), 1.35 (s, 3H, CH_3_-27), 1.20 (s, 3H, CH_3_-25), 1.15 (s, 3H, CH_3_-26), 1.11 (s, 3H, CH_3_-29), 0.93 (s, 3H, CH_3_-23), 0.87 (s, 3H, CH_3_-24), 0.82(s, 1H, CH-5), 0.80 (s, 3H, CH_3_-28); ^13^C-NMR (101 MHz, Chloroform-*d*) δ 199.98 (C11), 174.02 (C30), 169.63 (C13), 153.34 (carbamoyl), 139.30 (phenyl), 134.70 (phenyl), 129.94 (phenyl), 128.48 (C12), 123.18 (phenyl), 118.53 (phenyl), 116.42 (phenyl), 80.20 (C3), 66.94 (morpholine C), 61.65 (C9), 55.06 (C5), 48.22 (C18), 45.28 (C14), 43.78 (C20), 43.63 (morpholine C), 43.28 (C8), 38.75 (C1), 38.22 (C4), 37.67 (C22), 36.89 (C10), 33.28 (morpholine C), 32.70 (C7), 31.77 (C17), 28.41 (C28), 28.07 (C23), 26.96 (C2), 26.69 (C15), 26.40 (C16), 23.86 (C29), 23.11 (C27), 18.66 (C26), 17.35 (C6), 16.80 (C25), 16.41 (C24); HRMS (*m*/*z*): [M + Na]^+^ calcd. for C_41_H_57_ClN_2_NaO_5_: 715.38537, found: 715.38920.

*3β-(((3-chloro-4-methylphenyl)carbamoyl)oxy)-30-morpholino-olean-12-ene-11,30-dione* (**4f**), white solid; Yield, 91.8%; m.p. 293.7–295.6 °C; ^1^H-NMR (400 MHz, Chloroform-*d*) δ 7.50 (s, 1H, phenyl-H), 7.12 (s, 2H, phenyl-H), 6.57 (s, 1H, N-H), 5.69 (s, 1H, CH-12), 4.49 (dd, *J* = 10.3, 6.1 Hz, 1H, CH-3), 3.64 (qd, *J* = 8.5, 8.1, 3.5 Hz, 8H, morpholine-H), 2.81 (dt, *J* = 13.6, 3.6 Hz, 1H, CH-1), 2.36 (s, 1H, CH-9), 2.33–2.28 (m, 3H, CH_3_), 2.25 (d, *J* = 3.2 Hz, 1H, CH-16), 1.36 (s, 3H, CH_3_-27), 1.21 (s, 3H, CH_3_-25), 1.16 (s, 3H, CH_3_-26), 1.12 (s, 3H, CH_3_-29), 0.94 (s, 3H, CH_3_-23), 0.88 (s, 3H, CH_3_-24), 0.83 (s, 1H, CH-5), 0.81 (s, 3H, CH_3_-28); ^13^C-NMR (101 MHz, Chloroform-*d*) δ 199.98 (C11), 174.02 (C30), 169.59 (C13), 153.45 (carbamoyl), 136.85 (phenyl), 134.50 (phenyl), 130.95 (phenyl), 130.62 (phenyl), 128.49 (C12), 119.10 (phenyl), 116.78 (phenyl), 81.85 (C3), 66.95 (morpholine C), 61.67 (C9), 55.07 (C5), 48.23 (C18), 45.29 (C14), 43.78 (C20), 43.62 (morpholine C), 43.28 (C8), 38.77 (C1), 38.23 (C4), 37.67 (C22), 36.90 (C10), 33.30 (morpholine C), 32.71 (C7), 31.77 (C17), 28.41 (C28), 28.08 (C23), 26.97 (C2), 26.69 (C15), 26.41 (C16), 23.87 (C29), 23.12 (C27), 19.32 (CH_3_), 18.67 (C26), 17.35 (C6), 16.80 (C25), 16.41 (C24); HRMS (*m*/*z*): [M + Na]^+^ calcd. for C_42_H_59_ClN_2_NaO_5_: 729.40102, found: 729.40510.

*3β-(((4-bromophenyl)carbamoyl)oxy)-30-morpholino-olean-12-ene-11,30-dione* (**4g**), white solid; Yield, 90.6%; m.p. 309.7–3101 °C; ^1^H-NMR (400 MHz, Chloroform-*d*) δ 7.38 (dd, *J* = 8.9, 2.1 Hz, 2H, phenyl-H), 7.28 (d, *J* = 8.4 Hz, 2H, phenyl-H), 6.66 (s, 1H, N-H), 5.68 (d, *J* = 1.9 Hz, 1H, CH-12), 4.48 (dd, *J* = 11.3, 5.5 Hz, 1H, CH-3), 3.73–3.55 (m, 8H, morpholine-H), 2.85–2.75 (m, 1H, CH-1), 2.35 (s, 1H, CH-9), 2.27 (d, *J* = 12.8 Hz, 1H, CH-16), 1.35 (s, 3H, CH_3_-27), 1.20 (s, 3H, CH_3_-25), 1.14 (s, 3H, CH_3_-26), 1.10 (s, 3H, CH_3_-29), 0.93 (s, 3H, CH_3_-23), 0.87 (s, 3H, CH_3_-24), 0.82(s, 1H, CH-5), 0.80 (s, 3H, CH_3_-28); ^13^C-NMR (101 MHz, Chloroform-*d*) δ 199.97 (C11), 174.01 (C30), 169.62 (C13), 153.42 (carbamoyl), 137.22 (phenyl), 131.90 (phenyl), 128.48 (C12), 120.05 (phenyl), 115.62 (phenyl), 81.95 (C3), 66.94 (morpholine C), 61.66 (C9), 55.06 (C5), 48.21 (C18), 45.28 (C14), 43.78 (C20), 43.64 (morpholine C), 43.28 (C8), 38.76 (C1), 38.23 (C4), 37.67 (C22), 36.89 (C10), 33.26 (morpholine C), 32.70 (C7), 31.77 (C17), 28.41 (C28), 28.08 (C23), 26.96 (C2), 26.69 (C15), 26.40 (C16), 23.87 (C29), 23.11 (C27), 18.66 (C26), 17.35 (C6), 16.80 (C25), 16.41 (C24); HRMS (*m*/*z*): [M + Na]^+^ calcd. for C_41_H_57_BrN_2_NaO_5_: 759.33486, found: 759.33486, 761.33783.

*3β-(((4-fluorophenyl)carbamoyl)oxy)-30-morpholino-olean-12-ene-11,30-dione* (**4h**), white solid; Yield, 94.0%; m.p. 305.1–311.4 °C; ^1^H-NMR (400 MHz, Chloroform-*d*) δ 7.33 (s, 2H, phenyl-H), 6.97 (td, *J* = 8.6, 1.5 Hz, 2H, phenyl-H), 6.62 (s, 1H, N-H), 5.67 (s, 1H, CH-12), 4.48 (dd, *J* = 10.3, 6.1 Hz, 1H, CH-3), 3.65-3.59 (m, 8H, morpholine-H), 2.80 (dt, *J* = 14.1, 3.6 Hz, 1H, CH-1), 2.35 (s, 1H, CH-9), 2.31–2.22 (m, 1H, CH-16), 1.35 (s, 3H, CH_3_-27), 1.20 (s, 3H, CH_3_-25), 1.14 (s, 3H, CH_3_-26), 1.11 (s, 3H, CH_3_-29), 0.93 (s, 3H, CH_3_-23), 0.86 (s, 3H, CH_3_-24), 0.82 (s, 1H, CH-5), 0.80 (s, 3H, CH_3_-28); ^13^C-NMR (101 MHz, Chloroform-*d*) δ 199.99 (C11), 174.02 (C30), 169.60 (C13), 153.76 (carbamoyl), 134.05 (phenyl), 128.48 (C12), 120.14 (phenyl), 115.58 (d, *J* = 22.0 Hz, phenyl), 81.70 (C3), 66.94 (morpholine C), 61.67 (C9), 55.06 (C5), 48.21 (C18), 45.28 (C14), 43.78 (C20), 43.63 (morpholine C), 43.28 (C8), 38.77 (C1), 38.24 (C4), 37.67 (C22), 36.90 (C10), 33.27 (morpholine C), 32.71 (C7), 31.77 (C17), 28.41 (C28), 28.07 (C23), 26.96 (C2), 26.69 (C15), 26.40 (C16), 23.89 (C29), 23.10 (C27), 18.66 (C26), 17.35 (C6), 16.79 (C25), 16.41 (C24); HRMS (*m*/*z*): [M + Na]^+^ calcd. for C_41_H_58_FN_2_O_5_: 677.43298, found: 677.43850.

*3β-(((4-(trifluoromethyl)phenyl)carbamoyl)oxy)-30-morpholino-olean-12-ene-11,30-dione* (**4i**), white solid; Yield, 91.4%; m.p. 296.4–298.0 °C; ^1^H-NMR (400 MHz, Chloroform-*d*) δ 7.52 (q, *J* = 8.8 Hz, 4H, phenyl-H), 6.83 (s, 1H, N-H), 5.68 (s, 1H, CH-12), 4.51 (dd, *J* = 10.8, 5.6 Hz, 1H, CH-3), 3.70–3.56 (m, 8H, morpholine-H), 2.82 (dt, *J* = 13.6, 3.6 Hz, 1H, CH-1), 2.35 (s, 1H, CH-9), 2.28 (dd, *J* = 13.9, 3.6 Hz, 1H, CH-16), 1.35 (s, 3H, CH_3_-27), 1.21 (s, 3H, CH_3_-25), 1.15 (s, 3H, CH_3_-26), 1.11 (s, 3H, CH_3_-29), 0.94 (s, 3H, CH_3_-23), 0.88 (s, 3H, CH_3_-24), 0.83 (s, 1H, CH-5), 0.80 (s, 3H, CH_3_-28); ^13^C-NMR (101 MHz, Chloroform-*d*) δ 199.96 (C11), 174.01 (C30), 169.65 (C13), 153.23 (carbamoyl), 141.24 (phenyl), 128.48 (C12), 126.28 (q, *J* = 3.8 Hz, CF_3_), 125.50 (phenyl), 125.10 (phenyl), 124.78 (phenyl), 122.80 (phenyl), 117.93 (phenyl), 82.20 (C3), 66.94 (morpholine C), 61.65 (C9), 55.06 (C5), 48.20 (C18), 45.28 (C14), 43.78 (C20), 43.66 (morpholine C), 43.28 (C8), 38.75 (C1), 38.23 (C4), 37.67 (C22), 36.89 (C10), 33.24 (morpholine C), 32.70 (C7), 31.77 (C17), 28.41(C28), 28.08 (C23), 26.96 (C2), 26.68 (C15), 26.40 (C16), 23.85 (C29), 23.10 (C27), 18.66 (C26), 17.35 (C6), 16.80 (C25), 16.42 (C24); HRMS (*m*/*z*): [M + Na]^+^ calcd. for C_42_H_57_F_3_N_2_NaO_5_: 749.41173, found: 749.41688.

*3β-(((3-(trifluoromethyl)phenyl)carbamoyl)oxy)-30-morpholino-olean-12-ene-11,30-dione* (**4j**), white solid; Yield, 91.5%; m.p. 295.0–296.7 °C; ^1^H-NMR (400 MHz, Chloroform-*d*) δ 7.76 (s, 1H, phenyl-H), 7.56 (d, *J* = 8.1 Hz, 1H, phenyl-H), 7.41 (t, *J* = 8.0 Hz, 1H, phenyl-H), 7.30 (d, *J* = 7.7 Hz, 1H, phenyl-H), 6.86 (s, 1H, N-H H), 5.70 (s, 1H, CH-12), 4.53 (dd, *J* = 10.5, 6.0 Hz, 1H, CH-3), 3.66 (dd, *J* = 10.9, 5.4 Hz, 8H, morpholine-H), 2.83 (dt, *J* = 13.7, 3.7 Hz, 1H, CH-1), 2.37 (s, 1H, CH_3_-27), 2.34–2.25 (m, 1H, CH-9), 1.37 (s, 3H), 1.23 (s, 3H, CH_3_-25), 1.17 (s, 3H, CH_3_-26), 1.13 (s, 3H, CH_3_-29), 0.96 (s, 3H, CH_3_-23), 0.90 (s, 3H, CH_3_-24), 0.85 (s, 1H, CH-5), 0.82 (s, 3H, CH_3_-28); ^13^C-NMR (101 MHz, cdcl_3_) δ 199.98 (C11), 174.04 (C30), 169.66 (C13), 153.43 (carbamoyl), 138.71 (phenyl), 131.40 (q, *J* = 32.4 Hz, CF_3_), 129.50 (C12), 128.49 (C12), 125.23 (phenyl), 122.52 (phenyl), 121.43 (phenyl), 119.75 (phenyl), 115.21 (phenyl), 82.17 (C3), 66.95 (morpholine C), 61.67 (C9), 55.09 (C5), 48.23 (C18), 45.30 (C14), 43.80 (C20), 43.66 (morpholine C), 43.30 (C8), 38.77 (C1), 38.23 (C4), 37.69 (C22), 36.91 (C10), 33.28 (morpholine C), 32.71 (C7), 31.78 (C17), 28.42 (C28), 28.10 (C23), 26.97 (C2), 26.70 (C15), 26.42 (C16), 23.87 (C29), 23.12 (C27), 18.68 (C26), 17.36 (C6), 16.80 (C25), (C24); HRMS (*m*/*z*): [M + Na]^+^ calcd. for C_42_H_58_F_3_N_2_O_5_: 727.42978, found: 727.43507.

*3β-(((3,5-bis(trifluoromethyl)phenyl)carbamoyl)oxy)-30-morpholino-olean-12-ene-11,30-dione* (**4k**), white solid; Yield, 88.9%; m.p. 313.4–314.7 °C; ^1^H-NMR (400 MHz, Chloroform-*d*) δ 7.92 (s, 2H, phenyl-H), 7.52 (s, 1H, phenyl-H), 7.16 (d, *J* = 2.5 Hz, 1H, N-H), 5.69 (d, *J* = 2.5 Hz, 1H, CH-12), 4.52 (dd, *J* = 11.3, 5.7 Hz, 1H, CH-3), 3.68–3.64 (m, 8H, morpholine-H), 2.82 (dd, *J* = 13.7, 3.3 Hz, 1H, CH-1), 2.35 (s, 1H, CH-9), 2.28 (d, *J* = 13.6 Hz, 1H, CH-16), 1.35 (s, 3H, CH_3_-27), 1.21 (s, 3H, CH_3_-25), 1.15 (s, 3H, CH_3_-26), 1.11 (s, 3H, CH_3_-29), 0.94 (s, 3H, CH_3_-23), 0.88 (s, 3H, CH_3_-24), 0.83 (s, 1H, CH-5), 0.80 (s, 3H, CH_3_-28); ^13^C-NMR (101 MHz, Chloroform-*d* δ 199.96 (C11), 174.04 (C30), 169.76 (C13), 153.19 (carbamoyl), 139.73 (phenyl), 132.80 (CF_3_), 132.47 (CF_3_), 132.14 (CF_3_), 131.81 (CF_3_), 128.46 (C12), 127.16 (phenyl), 124.45 (phenyl), 121.74 (phenyl), 118.08 (phenyl), 116.35 (phenyl), 82.78 (C3), 66.93 (morpholine C), 61.62 (C9), 55.07 (C5), 48.20 (C18), 45.28 (C14), 43.79 (C20), 43.67 (morpholine C), 43.29 (C8), 38.72 (C1), 38.19 (C4), 37.67 (C22), 36.88 (C10), 33.23 (morpholine C), 32.68 (C7), 31.76 (C17), 28.41(C28), 28.09 (C23), 26.94 (C2), 26.68 (C15), 26.40 (C16), 23.81 (C29), 23.10 (C27), 18.66 (C26), 17.34 (C6), 16.77 (C25), 16.39 (C24); HRMS (*m*/*z*): [M + Na]^+^ calcd. for C_43_H_56_F_6_N_2_NaO_5_: 817.39911, found: 817.40464.

*3β-(((3-methoxyphenyl)carbamoyl)oxy)-30-morpholino-olean-12-ene-11,30-dione* (**4l**), white solid; Yield, 92.2%; m.p. 305.0–306.8 °C; ^1^H-NMR (400 MHz, Chloroform-*d*) δ 7.14 (s, 1H, phenyl-H), 6.85 (d, *J* = 8.1 Hz, 1H, phenyl-H), 6.62 (s, 1H, N-H), 6.60–6.57 (m, 1H, phenyl-H), 5.67 (s, 1H, CH-12), 4.49 (dd, *J* = 10.4, 6.1 Hz, 1H, CH-3), 3.81–3.75 (m, 3H, CH_3_), 3.68–3.55 (m, 8H, morpholine-H), 2.85–2.75 (m, 1H, CH-1), 2.35 (s, 1H, CH-9), 2.31–2.22 (m, 1H, CH-16), 1.35 (s, 3H, CH_3_-27), 1.21 (s, 3H, CH_3_-25), 1.15 (s, 3H, CH_3_-26), 1.11 (s, 3H, CH_3_-29), 0.94 (s, 3H, CH_3_-23), 0.88 (s, 3H, CH_3_-24), 0.83 (s, 1H, CH-5), 0.80 (s, 3H, CH_3_-28); ^13^C-NMR (101 MHz, Chloroform-*d*) δ 199.99 (C11), 174.02 (C30), 169.56 (C13), 160.22 (phenyl), 153.51 (carbamoyl), 139.33 (phenyl), 129.67 (phenyl), 128.49 (C12), 110.66 (phenyl), 109.12 (phenyl), 103.96 (phenyl), 81.68 (C3), 66.94 (morpholine C), 61.68 (C9), 55.24(CH_3_), 55.10 (C5), 48.22 (C18), 45.28 (C14), 43.78 (C20), 43.61(morpholine C), 43.27 (C8), 38.79 (C1), 38.23 (C4), 37.67 (C22), 36.90 (C10), 33.30 (morpholine C), 32.72 (C7), 31.77 (C17), 28.41(C28), 28.07 (C23), 26.97 (C2), 26.69 (C15), 26.40 (C16), 23.89 (C29), 23.09 (C27), 18.66 (C26), 17.35 (C6), 16.81(C25), 16.41 (C24); HRMS (*m*/*z*): [M + Na]^+^ calcd. for C_42_H_60_N_2_NaO_6_: 711.43491, found: 711.44018.

*3β-(((4-methoxyphenyl)carbamoyl)oxy)-30-morpholino-olean-12-ene-11,30-dione* (**4m**), white solid; Yield, 92.6%; m.p. 305.0–306.7 °C; ^1^H-NMR (400 MHz, Chloroform-*d*) δ 7.28 (s, 2H, phenyl-H), 6.86–6.79 (m, 2H, phenyl-H), 6.50 (s, 1H, N-H), 5.67 (d, *J* = 1.7 Hz, 1H, CH-12), 4.47 (t, *J* = 8.4 Hz, 1H, CH-3), 3.76 (d, *J* = 1.4 Hz, 3H, CH_3_), 3.70–3.55 (m, 8H, morpholine-H), 2.79 (d, *J* = 13.5 Hz, 1H, CH-1), 2.35 (s, 1H, CH-9), 2.30–2.21 (m, 1H, CH-16), 1.35 (s, 3H, CH_3_-27), 1.21 (s, 3H, CH_3_-25), 1.15 (s, 3H, CH_3_-26), 1.11 (s, 3H, CH_3_-29), 0.93 (s, 3H, CH_3_-23), 0.86 (s, 3H, CH_3_-24), 0.82 (s, 1H, CH-5), 0.80 (s, 3H, CH_3_-28); ^13^C-NMR (101 MHz, Chloroform-*d*) δ 200.09 (C11), 174.08 (C30), 169.64 (C13), 155.74 (phenyl), 153.96 (carbamoyl), 131.14 (phenyl), 128.46 (C12), 120.34 (phenyl), 114.16 (phenyl), 81.39 (C3), 66.93 (morpholine C), 61.68 (C9), 55.48 (CH_3_), 55.06 (C5), 48.24 (C18), 45.30 (C14), 43.79 (C20), 43.60 (morpholine C), 43.28 (C8), 38.78 (C1), 38.25 (C4), 37.66 (C22), 36.90 (C10), 33.31 (morpholine C), 32.72 (C7), 31.77 (C17), 28.40 (C28), 28.06 (C23), 26.96 (C2), 26.69 (C15), 26.40 (C16), 23.90 (C29), 23.09 (C27), 18.66 (C26), 17.35 (C6), 16.80 (C25), 16.41 (C24); HRMS (*m*/*z*): [M + Na]^+^ calcd. for C_42_H_60_N_2_NaO_6_: 711.43491, found: 711.43961.

*3β-(((4-(trifluoromethoxy)phenyl)carbamoyl)oxy)-30-morpholino-olean-12-ene-11,30-dione* (**4n**), white solid; Yield, 94.0%; m.p. 308.0–310.1 °C; ^1^H-NMR (400 MHz, Chloroform-*d*) δ 7.43 (d, *J* = 8.5 Hz, 2H, phenyl-H), 7.16 (d, *J* = 8.4 Hz, 2H, phenyl-H), 6.75 (s, 1H, N-H), 5.70 (d, *J* = 1.9 Hz, 1H, CH-12), 4.51 (dd, *J* = 11.2, 5.5 Hz, 1H, CH-3), 3.67 (d, *J* = 5.3 Hz, 8H, morpholine-H), 2.87–2.78 (m, 1, CH-1H), 2.37 (s, 1H, CH-9), 2.29 (d, *J* = 12.8 Hz, 1H, CH-16), 1.37 (s, 3H, CH_3_-27), 1.22 (s, 3H, CH_3_-25), 1.17 (s, 3H, CH_3_-26), 1.13 (s, 3H, CH_3_-29), 0.96 (s, 3H, CH_3_-23), 0.89 (s, 3H, CH_3_-24), 0.85 (s, 1H, CH-5), 0.82 (s, 3H, CH_3_-28); ^13^C-NMR (101 MHz, Chloroform-*d*) δ 199.99 (C11), 174.03 (C30), 169.64 (C13), 153.54 (carbamoyl), 144.52 (phenyl), 136.85 (phenyl), 128.50 (C12), 121.85 (phenyl), 119.50 (CF_3_), 119.20 (phenyl), 81.97 (C3), 66.95 (morpholine C), 61.68 (C9), 55.08 (C5), 48.22 (C18), 45.30 (C14), 43.80 (C20), 43.67 (morpholine C), 43.30 (C8), 38.78 (C1), 38.25 (C4), 37.69 (C22), 36.91 (C10), 33.27 (morpholine C), 32.72 (C7), 31.78 (C17), 28.42(C28), 28.08 (C23), 26.97 (C2), 26.70 (C15), 26.42 (C16), 23.89 (C29), 23.12 (C27), 18.68 (C26), 17.36 (C6), 16.81 (C25), 16.43 (C24); HRMS (*m*/*z*): [M + Na]^+^ calcd. for C_42_H_57_F_3_N_2_NaO_6_: 765.40664, found: 765.41272.

#### 3.2.4. 3β-(2-chloroacetyloxy)-11-oxo-olean-12-en-30-oic acid **5**

A mixture of 18β–GA (0.47 g, 1.0 mmol) and chloroacetic anhydride (3.42 g, 20 mmol) was heated at 130 °C for 1 h. After the reaction was completed (monitored by by thin-layer chromatography), H_2_O (20 mL) was added to the cool solution, and the mixture was stirred for 30 min at the room temperature. The product was filtered off and washed with cold H_2_O.

A white solid; Yield, 98.0%; 280.7–281.7 °C. (literature [44]: 260.8–261.8 °C); ^1^H-NMR (400 MHz, Chloroform-*d*) δ 5.72 (s, 1H, CH-12), 4.61 (dd, *J* = 11.8, 4.8 Hz, 1H, CH-3), 4.06 (d, *J* = 2.3 Hz, 2H, CH_2_-Cl), 2.83 (m, 1H, CH-1), 2.37 (s, 1H, CH-9), 2.19 (dd, *J* = 13.6, 4.1 Hz, 1H, CH-16), 1.38 (m, 3H, CH_3_-27), 1.23 (s, 3H, CH_3_-25), 1.17 (s, 3H, CH_3_-26), 1.13 (s, 3H, CH_3_-29), 0.90 (s, 6H, CH_3_-23/24), 0.84 (s, 3H, CH_3_-28), 0.80 (m, 1H, CH-5); ^13^C-NMR (101 MHz, Chloroform-*d*) δ 200.26 (C11), 181.51(C30), 169.59 (C13), 167.12 (acetyloxy C=O), 128.37 (C12), 83.00 (C3), 61.60 (C9), 54.94 (C5), 48.21 (C18), 45.43 (C14), 43.78 (C20), 43.18 (C8), 41.24 (C19), 40.80 (C-Cl), 38.64 (C1), 38.23 (C4), 37.67 (C22), 36.87 (C10), 32.62 (C7), 31.84 (C17), 30.87 (C21), 28.52 (C29), 28.43 (C28), 28.00 (C23), 26.43 (C2), 26.34 (C15), 23.41 (C16), 23.35 (C27), 18.64 (C26), 17.30 (C6), 16.61 (C25), 16.39 (C24); HRMS (*m*/*z*): [M + H]^+^ calcd. for C_32_H_48_ClO_5_: 547.3190, found: 547.3188.

#### 3.2.5. General Procedure for Preparation of Carbamate Derivatives (**6a**–**6d**)

Compound **5** (0.55 g, 1.0 mmol), substituted secondary amine (1.5 mmol), K_2_CO_3_ (0.69 g, 5.0 mmol), and a catalytic amount of I_2_ in absolute ethanol (15 mL) was stirred under reflux for 12 h. The reaction mixture was evaporated under reduced pressure and the residue was dissolved in ethanol/H_2_O mixture and the white precipitate was collected by filtration.

*3β-(2-morpholinoacetoxy)-11-oxo-olean-12-en-30-oic acid* (**6a**), white solid; Yield, 92.0%; m.p.275.3–276.4 °C; ^1^H-NMR (400 MHz, Chloroform-*d*) δ 5.72 (t, *J* = 3.0 Hz, 1H, CH-12), 4.60 (dd, *J* = 11.2, 5.5 Hz, 1H, CH-3), 3.76 (d, *J* = 4.9 Hz, 4H, morpholine), 3.26–3.20 (m, 2H, CH_2_), 2.80 (d, *J* = 12.9 Hz, 1H, CH-1), 2.63 (s, 4H, morpholine), 2.37 (t, *J* = 3.0 Hz, 1H, CH-9), 2.19 (d, *J* = 13.5 Hz, 1H, CH-16), 1.37 (s, 3H, CH_3_-27), 1.27 (s, 3H, CH_3_-25), 1.16 (s, 3H, CH_3_-26), 1.13 (s, 3H, CH_3_-29), 0.87 (s, 3H, CH_3_-23/24), 0.83 (s, 3H, CH_3_-28), 0.80 (s, 1H, CH-5); ^13^C-NMR (101 MHz, Chloroform-*d*) δ 200.25 (C11), 181.03 (C30), 169.70 (C13), 169.51 (Acetoxy, C=O), 128.40 (C12), 81.09 (C3), 66.67 (morpholine C), 61.64 (C9), 59.53 (Acetoxy, CH_2_), 54.93 (C5), 53.14 (morpholine C), 48.25 (C18), 45.43 (C14), 43.75 (C20), 43.19 (C8), 40.90 (C19), 38.68 (C1), 38.07 (C4), 37.70 (C22), 36.89 (C10), 32.65 (C7), 31.86 (C17), 30.93 (C21), 28.55 (C29), 28.44 (C28), 28.13 (C23), 26.46 (C2), 26.37 (C15), 23.66 (C16), 23.36 (C27), 18.66 (C26), 17.36 (C6), 16.78 (C25), 16.42 (C24); HRMS (*m*/*z*): [M + Na]^+^ calcd. for C_36_H_56_NO_6_: 598.41076, found: 598.41500.

*3β-(2-(4-carbamoylpiperidin-1-yl)acetoxy)-11-oxo-olean-12-en-30-oic acid* (**6b**), white solid; Yield, 91.1%; m.p. 283.5–285.0 °C; ^1^H-NMR (400 MHz, Chloroform-*d*) δ 5.67 (s, 1H, CH-12), 4.74 (s, 2H, NH_2_), 4.58 (dd, *J* = 11.6, 4.7 Hz, 1H, CH-3), 3.93 (t, *J* = 5.0 Hz, 2H, -CH_2_-C=O), 3.68 (dt, *J* = 8.8, 5.0 Hz, 4H, -CH_2_-N-CH_2_-), 3.29 (s, 2H, -CH_2_-C=O), 2.79 (d, *J* = 13.5 Hz, 1H, -CH-CONH_2_), 2.62 (dt, *J* = 11.0, 5.1 Hz, 4H, -CH_2_-N-CH_2_-), 2.35 (s, 1H, CH-9), 2.19 (dd, *J* = 13.4, 4.1 Hz, CH-16), 1.37 (s, 3H, CH_3_-27), 1.18 (s, 3H, CH_3_-25), 1.14 (s, 3H, CH_3_-26), 1.12 (s, 3H, CH_3_-29), 0.86 (s, 3H, CH_3_-23/24), 0.81 (s, 3H, CH_3_-28), 0.79 (s, 1H, CH-5); ^13^C-NMR (101 MHz, Chloroform-*d*) δ 200.09 (C11), 181.10 (C30), 169.95 (C13), 169.54 (-CONH_2_), 169.08 (-COOC-), 128.26 (C12), 81.41 (C3), 61.60 (C9), 54.88 (C5), 53.29 (-CH_2_-COO-), 53.29 (-CH_2_-N-CH_2_-), 52.95 (-CH_2_-N-CH_2_-), 52.72 (-CH_2_-N-CH_2_-), 52.44 (-CH_2_-N-CH_2_-), 52.10 (-CH_2_-N-CH_2_-), 48.43 (C18), 45.39 (C14), 43.92 (C20), 43.20 (C8), 41.26 (CH-CONH_2_), 41.06 (C19), 38.68 (C1), 38.04 (C4), 37.88 (C22), 36.85 (C10), 32.61(C7), 31.86 (C17), 31.16 (C21), 28.66 (C29), 28.60 (C28), 28.14 (C23), 26.47 (C2), 26.39 (C16), 23.60 (C15), 23.35 (C27), 21.31[-(CH_2_)-CH-CONH_2_], 18.64 (C26), 17.34 (C6), 16.75 (C25), 16.40 (C24); HRMS (*m*/*z*): [M + Na]^+^ calcd. for C_38_H_58_N_2_NaO_6_: 661.41926, found: 661.41747.

*3β-(2-(4-methylpiperazin-1-yl)acetoxy)-11-oxo-olean-12-en-30-oic acid* (**6c**), white solid; Yield, 89.9%; m.p.287.5–288.9 °C; ^1^H-NMR (400 MHz, Chloroform-*d*) δ 5.70 (s, 1H, CH-12), 4.59 (dd, *J* = 11.7, 4.7 Hz, 1H, CH-3), 3.27 (s, 2H, -CH_2_-C=O), 3.14 (q, *J* = 7.3 Hz, 4H, 4-methylpiperazin-1-yl, CH_2_), 2.87 (s, 4H, 4-methylpiperazin-1-yl, CH_2_), 2.55 (s, 3H, CH_3_-piperazin-1-yl), 2.36 (s, 1H, CH-9), 2.20 (d, *J* = 12.0 Hz, 1H, CH-16), 1.37 (s, 3H, CH_3_-27), 1.20 (s, 3H, CH_3_-25), 1.15 (s, 3H, CH_3_-26), 1.13 (s, 3H, CH_3_-29), 0.86 (s, 3H, CH_3_-23/24), 0.82 (s, 3H, CH_3_-28), 0.79 (s, 1H, CH-5); ^13^C-NMR (101 MHz, Chloroform-*d*) δ 200.08 (C11), 179.96 (C30), 169.62 (C13/ -COOC-), 128.36 (C12), 81.35 (C3), 61.61 (C9), 58.65 (-CH_2_-COO-), 54.91 (C5), 53.87 (-CH_2_-COO-), 48.37 (C18), 45.93 (4-methylpiperazin-1-yl, CH_2_), 45.38 (C14), 44.33 (CH_3_-piperazin-1-yl), 43.78 (C20), 43.18 (C8), 41.18 (C19), 38.68 (C1), 38.05 (C4), 37.80 (C22), 36.87 (C10), 32.63 (C7), 31.85 (C17), 31.10 (C21), 28.60 (C28/29), 28.51(C29), 28.13 (C23), 26.46 (C2), 26.39 (C16), 23.60 (C15), 23.35 (C27), 18.65 (C26), 17.35 (C6), 16.74 (C25), 16.40 (C24); HRMS (*m*/*z*): [M + H]^+^ calcd. for C_37_H_59_N_2_O_5_: 611.44240, found: 611.44228.

*3β-(2-(4-(pyridin-2-yl)piperazin-1-yl)acetoxy)-11-oxo-olean-12-en-30-oic acid* (**6d**), white solid; Yield, 88.6%; m.p. 294.0–295.6 °C; ^1^H-NMR (400 MHz, Chloroform-*d*) δ 8.21 (dd, *J* = 5.0, 1.8 Hz, 1H, pyridyl-H), 7.59–7.44 (m, 1H, pyridyl-H), 6.71–6.61 (m, 2H, pyridyl-H), 5.71 (s, 1H, CH-12), 4.61 (dd, *J* = 11.6, 4.8 Hz, 1H, CH-3), 3.63 (t, *J* = 5.0 Hz, 4H, piperazinyl-H), 3.30 (s, 2H, -CH_2_-C=O), 2.82–2.71 (m, 4H, piperazinyl-H), 2.37 (s, 1H, CH-9), 2.19 (dd, *J* = 13.4, 4.2 Hz, 1H, CH-16), 1.37 (s, 3H, CH_3_-27), 1.22 (s, 3H, CH_3_-25), 1.15 (s, 3H, CH_3_-26), 1.12 (s, 3H, CH_3_-29), 0.88 (s, 3H, CH_3_-23), 0.87 (s, 3H, CH_3_-24), 0.83 (s, 3H, CH_3_-28), 0.80 (s, 1H, CH-5); ^13^C-NMR (101 MHz, Chloroform-*d*) δ 200.20 (C11), 180.64 (C30), 169.80 (C13), 169.49 (-COOC-), 158.93 (pyridin-2-yl C), 147.29 (pyridin-2-yl C), 137.93 (pyridin-2-yl C), 128.38 (C12), 113.36 (pyridin-2-yl C), 107.47(pyridin-2-yl C), 81.16 (C3), 61.62 (C9), 59.26 (-CH_2_-COO-), 54.92 (C5), 52.54 (piperazinyl C), 48.24 (C18), 45.41 (C14), 45.15 (piperazinyl C), 43.73 (C20), 43.17 (C8), 40.92 (C19), 38.68 (C1), 38.06 (C4), 37.68 (C22), 36.87 (C10), 32.64 (C7), 31.85 (C17), 30.93 (C21), 28.52 (C28), 28.44 (C29), 28.14 (C23), 26.44 (C2), 26.36 (C16), 23.65 (C15), 23.35 (C27), 18.64 (C26), 17.35 (C6), 16.77 (C25), 16.40 (C24); HRMS (*m*/*z*): [M + H]^+^ calcd. for C_41_H_60_N_3_O_5_: 674.45330, found: 674.45080.

#### 3.2.6. General Procedure for Preparation of Carbamate Derivatives (**7a**–**7b**)

18β-GA (0.47 g, 1.0 mmol) was dissolved in ethyl acetate (15 mL) and triethylamine (0.39 g, 3.6 mmol) was added. While the mixture was stirred at room temperature, substituted acyl chloride (3.0 mmol) was added dropwise into the solution. After be stirred under reflux for 24 h, the reaction mixture was poured into water (40 mL). The organic layer was washed with 5% of aqueous NaHCO_3_, brine and was dried over anhydrous Na_2_SO_4_. The organic layer was then concentrated under reduced pressure. The residue obtained was purified by silica gel column chromatography using CH_2_Cl_2_–CH_3_OH as the eluent.

*3β-(2-(4-chlorophenyl)acetoxy)-11-oxo-olean-12-en-30-oic acid* (**7a**), white solid; Yield, 97.0%; m.p. 312.0–312.7 °C; ^1^H-NMR (400 MHz, Chloroform-*d*) δ 13.09 (s, 1H, -CH=C-OH), 7.32 (m, 2H, phenyl-H), 7.23 (m, 2H, phenyl-H), 7.04 (m, 2H, phenyl-H), 5.70 (s, 1H, CH-12), 4.76 (d, *J* = 7.1 Hz, 1H, -CH=C-OH), 4.46–4.40 (m, 1H, CH-3), 3.58 (s, 1H, -CH_2_-C=O), 3.38 (s, 1H, -CH=C-OH), 2.83–2.74 (m, 1H, CH-1), 2.35 (s, 1H, CH-9), 2.18 (d, *J* = 12.3 Hz, 1H, CH-16),1.36 (s, 3H, CH_3_-27), 1.22 (s, 3H, CH_3_-25), 1.14 (s, 3H, CH_3_-26), 1.12 (s, 3H, CH_3_-29), 0.83 (s, 3H, CH_3_-23), 0.80 (s, 3H, CH_3_-24), 0.75 (s, 3H, CH_3_-28), 0.72 (s, 1H, CH-5); ^13^C-NMR (101 MHz, Chloroform-*d*) δ 200.39, 200.29, 200.23, 181.25, 173.18, 172.11, 170.87, 169.48, 134.70, 134.47, 133.20, 133.12, 132.86, 132.79, 132.65, 131.38, 130.95, 130.89, 130.85, 130.63, 130.21, 129.01, 128.96, 128.90, 128.87, 128.60, 128.55, 128.38, 104.33, 81.76, 81.28, 63.34, 61.61, 54.91, 54.84, 48.20, 48.00, 47.95, 45.41, 45.39, 43.75, 43.16, 43.14, 41.27, 40.79, 38.58, 38.23, 38.17, 38.11, 38.04, 37.66, 36.86, 36.83, 36.80, 32.60, 31.83, 30.87, 28.51, 28.41, 28.01, 27.91, 26.42, 26.34, 23.47, 23.34, 18.63, 18.61, 17.27, 16.61, 16.36, 16.33, 16.29, 16.13; HRMS (*m*/*z*): [M + Na]^+^ calcd. for C_38_H_51_ClNaO_5_: 645.33227, found: 645.33653.

*3β-(2-(4- fluorophenyl)acetoxy)-11-oxo-olean-12-en-30-oic acid* (**7b**), white solid; Yield, 85.1%; m.p. 305.0–307.7 °C; ^1^H-NMR (400 MHz, Chloroform-*d*) δ 13.05 (s, 1H, -CH=C-OH), 7.30–7.20 (m, 1H, phenyl-H), 7.15–6.77 (m, 5H, phenyl-H), 5.68 (s, 1H, CH-12), 4.76 (d, *J* = 8.3 Hz, 1H, -CH=C-OH), 4.58–4.42 (m, 1H, CH-3), 3.57 (s, 1H, -CH_2_-C=O), 3.37 (s, 1H, -CH=C-OH), 2.82–2.70 (m, 1H, CH-1), 2.32 (s, 1H, CH-9), 2.16 (d, *J* = 13.1 Hz, 1H, CH-16), 1.34 (s, 3H, CH_3_-27), 1.20 (s, 3H, CH_3_-25), 1.14 (s, 3H, CH_3_-26), 1.10 (s, 3H, CH_3_-29), 0.80 (s, 3H, CH_3_-23), 0.78 (s, 3H, CH_3_-24), 0.73 (s, 3H, CH_3_-28), 0.70 (s, 1H, CH-5); ^13^C-NMR (101 MHz, Chloroform-*d*) δ 200.35, 200.30, 200.25, 181.69, 173.49, 172.34, 171.17, 169.63, 169.56, 167.94, 167.84, 163.11, 162.96, 161.42, 161.37, 160.82, 160.73, 160.67, 160.52, 131.98, 131.38, 131.30, 131.27, 131.19, 131.14, 131.11, 131.08, 131.06, 131.03, 131.00, 130.84, 130.76, 130.70, 130.55, 130.44, 130.38, 130.36, 130.31, 130.09, 130.06, 128.76, 128.36, 128.19, 127.97, 115.88, 115.83, 115.74, 115.71, 115.67, 115.62, 115.53, 115.50, 115.46, 115.41, 115.37, 115.35, 115.32, 115.25, 115.19, 115.12, 115.11, 104.25, 82.52, 82.45, 81.63, 81.17, 63.17, 63.11, 61.61, 61.57, 54.97, 54.91, 54.83, 48.20, 47.84, 47.80, 45.42, 45.40, 43.78, 43.17, 43.14, 41.11, 40.77, 40.24, 39.94, 38.65, 38.59, 38.16, 38.11, 38.07, 38.02, 38.00, 37.66, 36.87, 36.83, 36.81, 32.60, 31.83, 30.85, 28.51, 28.43, 27.99, 27.92, 27.88, 26.42, 26.34, 23.47, 23.34, 23.32, 18.63, 18.60, 17.26, 16.59, 16.36, 16.33, 16.29, 16.24, 16.05; HRMS (*m*/*z*): [M + Na]^+^ calcd. for C_38_H_51_FNaO_5_: 629.36182, found: 629.35058.

### 3.3. Crystal Structure Analysis

Single crystals of compound **3j** was recrystallized by slow evaporation from methanol. Data collection was performed at 296 on a Bruker SMART APEX II CCD diffractometer (Bruker BioSpin, Rheinstetten, Germeny) using Mo Ka (λ = 1.54178 Å) radiation. The crystal structure was solved by direct methods using SHELXS-97 and final refinement, based on F^2^, was carried out by a full matrix least squares with SHELXL-97. Refinement was performed anisotropically for all nonhydrogen atoms. In general, the hydrogen atoms were assigned to idealized positions and allowed to ride on the parent atom.

### 3.4. Primary Anticancer Assay

All the cells were seeded in 96-well plates and incubated with 5% CO2 at 37 °C for 24 h. Next, the compounds and the reference were dissolved into the culture medium. The final concentration of DMSO in the medium was less than 0.5%. After the cells were treated with compounds for 48 h, the supernatant was removed and 5 mg/mL of a fresh prepared solution of MTT was added to each well and incubated with the cells at 37 °C for another 4 h. The medium was removed, and 100 μL of testing solution was added to each well. After an overnight incubation with 5% CO_2_ at 37 °C, the absorbance was measured at 490/630 nm by the microplate reader. The IC_50_ was calculated using GraphPad Prism version 6.0 software (San Diego, CA, USA) from the non-linear curve.

### 3.5. Kinase Activity Determination

The effects of the compounds on the activities of the tyrosine kinase was determined using enzyme-linked immunosorbent assay (ELISA). Briefly, 20 μg/mL substrate [poly (Glu,Tyr)_4:1_], 50 μL aliquot of 10 μmol/L ATP solution diluted in kinase buffer (50 mmol/L HEPES pH 7.5, 50 mmol/L MgCl_2_, 0.5 mmol/L MnCl_2_, 0.2 mmol/L Na_3_VO_4_, and 1 mmol/L DTT) was added to each well; 1 μL of various concentrations of target compound and positive control drug diluted in 1% DMSO (*v/v*) were then added to each well. 1% DMSO was used as the negative control. After 5–10 min preincubation, the kinase reaction was initiated by the addition of purified ALK proteins diluted in 49 μL of kinase buffer. After 30 min preincubation, the anti-phosphotyrosine monoclonalantibody (100 μL; 1:500, diluted in 5 mg/mL BSA T-PBS) was then added. After a 30 min incubation at 37 °C, and 100 μL horseradish peroxidase conjugated goat anti-mouse IgG (1:2000, diluted in 5 mg/mL BSA T-PBS) was added. The plate was then incubated at 37 °C for 30 min. A 100 μL aliquot of a solution containing 0.03% H_2_O_2_ and 2 mg/mL o-phenylenediamine in 0.1 mol/L citrate buffer was added. The reaction was terminated by the addition of 50 μL of 2 mol/L H_2_SO_4_ as the color changed, and the plate was analyzed using a multi-well spectrophotometer at 490 nm.

### 3.6. Molecular Modeling

The human ALK in complex with Crizotinib (PDB code: 2XP2) was retrieved from the Protein Data Bank (http://www.rcsb.org). Molecular docking was performed using the CDocker protocol (the Discovery Studio 3.5 software package, Accelrys, Co. Ltd., San Diego, CA, USA). Protein preparation was carried out using the Prepare Protein protocol, and all crystallographic water was removed from the protein. The ligands preparation was carried out using the Prepare Ligand protocol. The docking parameters were set as default. The lowest binding energy was taken as the best-docked conformation of the representative compound for the protein. Molecular docking was validated by the docking of the co-crystallized inhibitor for enzyme, and root-mean-square deviation (RMSD) value for the backbone atoms between docked pose and crystallographic pose was below 1.5 Å.

## 4. Conclusions

18β-GA is considered an interesting scaffold for the development of potential antitumor inhibitors. Current structural optimization of 18β-GA primarily focused on alternation in position C3 or C30-position, exhibited remarkable chemopreventive activities in various experimental cancer models. In the present study, 35 derivatives of 18β-GA, altered in C3 and/or C30-position, were developed and evaluated for their efficacy as antitumor inhibitors. Among the mentioned derivatives, the carboxyl group at the C30-position of 18β-GA is beneficial to improve the inhibitory potency. Compound **3j** exhibited the most excellent antiproliferative activity against six human cancer cells (A549, HT29, HepG2, MCF-7, PC-3, and Karpas299). Besides, compound **3j** inhibited the proliferation of HepG2 cell in a significant concentration manner and exhibited selective antiproliferative activity against human tumor cells. Furthermore, compound **3j** exerted moderate inhibitory effects against the ALK. The results of docking analysis suggested that the hydrogen bond interactions with kinase are conducive to the binding. These results may inspire further structural modifications of 18β-GA aimed at developing potent ALK inhibitors.

## Figures and Tables

**Table 1:** Details for the crystal structure determinations of compounds **4d**, **4g**, **4h**, **4j**, and **4n**.

Entry	4d	4g	4h	4j	4n
CCDC number	1953934	1953928	1953941	1913193	1952936
Crystal color	colorless	colorless	colorless	colorless	colorless
Solution	CH_3_OH	CH_3_OH	CH_3_OH	CH_3_OH	CH_3_OH
Crystal system	Orth^a^	Orth	Orth	Orth	Orth
Space group	P2_1_2_1_2_1_	P2_1_2_1_2_1_	P2_1_2_1_2_1_	P2_1_2_1_2_1_	P2_1_2_1_2_1_
a/Å	14.5561(7)	14.5119(7)	14.5570(11)	14.1203(8)	13.7916(9)
b/Å	15.3392(8)	15.4564(8)	15.2530(11)	15.6010(9)	16.7118(11)
c/Å	17.0499(9)	17.0665(8)	17.0544(19)	18.3201(10)	17.5923(12)
α (°)	90	90	90	90	90
β (°)	90	90	90	90	90
γ (°)	90	90	90	90	90
Volume/Å^3^	3806.9(3)	3828.0(3)	3786.7(6)	4035.7(4)	4054.7(5)
Temperature/K	296	296	296	296	296
*Z*	4	4	4	4	4
Density (calculated)/g·cm^−3^	1.210	1.280	1.187	1.196	1.217
*F*000	1496	1568.0	1464.0	1560.0	1592.0
Independent reflections	9492	7350	6813	7157	7151
Goodness-of-fit on F^2^	1.053	1.029	1.017	1.023	1.035
Final R indices (I > 2σ (I))	R_1_ = 0.0478	R_1_ = 0.0478	R_1_ = 0.0505	R_1_ = 0.0615	R_1_ = 0.0509
	wR_2_ = 0.1161	wR_2_ = 0.1097	wR_2_ = 0.1302	wR_2_ = 0.1622	wR_2_ = 0.1181
R indices (all data)	R_1_ = 0.0732	R_1_ = 0.0720	R_1_ = 0.0740	R_1_=0.1008	R_1_ = 0.0801
	wR_2_ = 0.1287	wR_2_ = 0.1198	wR_2_ = 0.1459	wR_2_=0.1895	wR_2_ = 0.1343
Largest diff. peak and hole/ e.Å^−3^	0.351 and −0.436	0.801 and −0.857	0.408 and −0.280	0.267 and −0.180	0.395 and −0.251

^a^ Orth, Orthorhombic.

**Table 2:** Antiproliferative activity of the target compounds (growth inhibition, %).

Entry	A549		HT29	
	2 μg/mL	20 μg/mL	2 μg/mL	20 μg/mL
**3a**	23.94	95.92	11.34	98.05
**3b**	13.96	77.35	14.12	87.72
**3c**	18.51	95.40	8.39	98.61
**3d**	9.93	55.43	13.73	56.25
**3e**	14.32	74.92	18.84	96.44
**3f**	11.36	80.21	15.58	89.22
**3g**	9.72	63.39	18.57	76.54
**3h**	34.44	56.72	11.78	58.59
**3i**	15.91	87.47	19.41	90.12
**3j**	17.74	88.31	24.79	99.56
**3k**	18.79	88.82	10.76	94.83
**3l**	9.74	53.73	6.06	47.44
**3m**	11.68	47.51	12.82	32.33
**3n**	20.08	97.07	18.99	97.65
**3o**	24.41	86.30	55.75	98.17
**4a**	0.00	0.00	8.88	18.34
**4b**	0.00	0.00	14.40	32.80
**4c**	0.00	0.00	7.41	2.76
**4d**	0.00	54.37	16.34	53.73
**4e**	0.00	0.00	7.22	13.25
**4f**	0.00	0.00	7.10	18.45
**4g**	0.00	1.02	14.34	27.12
**4h**	0.00	8.54	14.81	30.39
**4i**	0.00	4.46	13.70	27.32
**4j**	0.00	0.00	20.31	36.42
**4k**	4.47	0.00	13.04	14.91
**4l**	0.00	0.00	13.61	16.57
**4m**	0.00	8.11	15.69	23.22
**4n**	0.00	0.00	7.12	40.21
**6a**	0.00	0.00	0.00	0.00
**6b**	0.00	0.00	0.00	12.34
**6c**	0.00	0.00	0.00	0.00
**6d**	0.00	0.00	0.00	0.00
**7a**	11.35	27.45	33.27	57.45
**7b**	15.26	31.16	44.15	59.02
18β-GA	0.00	8.41	0.00	12.93
Crizotinib	67.61	97.29	68.80	99.79

**Table 3:** Antiproliferative activity (IC_50_, μg/mL) of selected compound.

Entry	A549	HT29	HepG2	MCF-7	PC-3	Karpas299
**3a**	11.19	11.44	11.6	9.31	11.91	12.30
**3b**	11.91	11.97	9.25	8.92	12.07	18.57
**3c**	12.28	9.74	10.68	5.53	14.98	15.47
**3e**	11.34	10.91	11.95	8.51	9.75	7.31
**3j**	2.81	3.19	5.55	5.26	5.96	5.59
**3k**	2.93	6.47	11.25	7	10.33	9.16
**3n**	7.42	11.33	12.55	9.30	13.32	25.97
**3o**	3.24	7.30	4.75	7.86	10.69	11.44
18β-GA	>40	>40	>40	>40	>40	>40
Crizotinib	1.27	0.67	2.15	1.84	3.3	0.28

**Table 4:** Inhibitory effects of selected compounds on ALK activity.

Compound	3j	3k	Crizotinib
IC_50_ (nM)	120.68	278.21	1.64

## Supplementary Materials

^1^H-NMR and ^13^C-NMR spectra for the prepared compounds are available online.
