# Supplementary material for: Exploring New Structural Features of the 18β-Glycyrrhetinic Acid Scaffold for the Inhibition of Anaplastic Lymphoma Kinase
Source: Molecules. 2019 Oct 8;24(19):3631. doi: 10.3390/molecules24193631 (PMC6803848; doi:10.3390/molecules24193631)

molecules

MDPI

Supplementary Materials

Exploring New Structural Features of The 18β-Glycyrrhetinic Acid Scaffold for The Inhibition of Anaplastic Lymphoma Kinase

Dong Cai [1], ZhiHua Zhang [2], Yu Chen [3], YanYan Zhang [4], YuQi Sun [4*] and YiXia Gong [1,*]

[1] College of Public Basic Sciences, Jinzhou Medical University, Jinzhou 121001, China
[2] School of Chemical and Environmental Engineering, Liaoning University of Technology, Jinzhou 121001, China
[3] School of Life Science and Biopharmaceutics, Shenyang Pharmaceutical University, Shenyang 110016, China
[4] School of Chemical and Environmental Engineering, Liaoning University of Technology, Jinzhou,121001, China
* Correspondence: cpusyq@jzmu.edu.cn (Y.S.); gongyixia_2006@163.com (Y.G.); Tel.: +86-0461-467-3404 (Y.S.)

Table of Contents.

^{1}H- and ^{13}C-NMR and HRMS of compound (**2**)……...………...………...……...………S2

^{1}H- and ^{13}C-NMR and HRMS of compound (**2a**) ……...…...………...………...………S3

^{1}H- and ^{13}C-NMR and HRMS of compound (**3a–3o**) ……...……...……...………..…S4-18

^{1}H-and ^{13}C-NMR and HRMS of compound (**4a–4p**) ……...……...……...…………S19-32

^{1}H-and ^{13}C-NMR and HRMS of compound (**5**) ……...……………...………...……..…S33

^{1}H- and ^{13}C-NMR and HRMS of compound (**6a–6d**) ……...………...…………...…S34-37

^{1}H- and ^{13}C-NMR and HRMS of compound (**7a–7b**) ……...………...………...……S38-39

3*β*-Hydroxy-30-morpholino-olean-12-ene-11,30-dione **2**

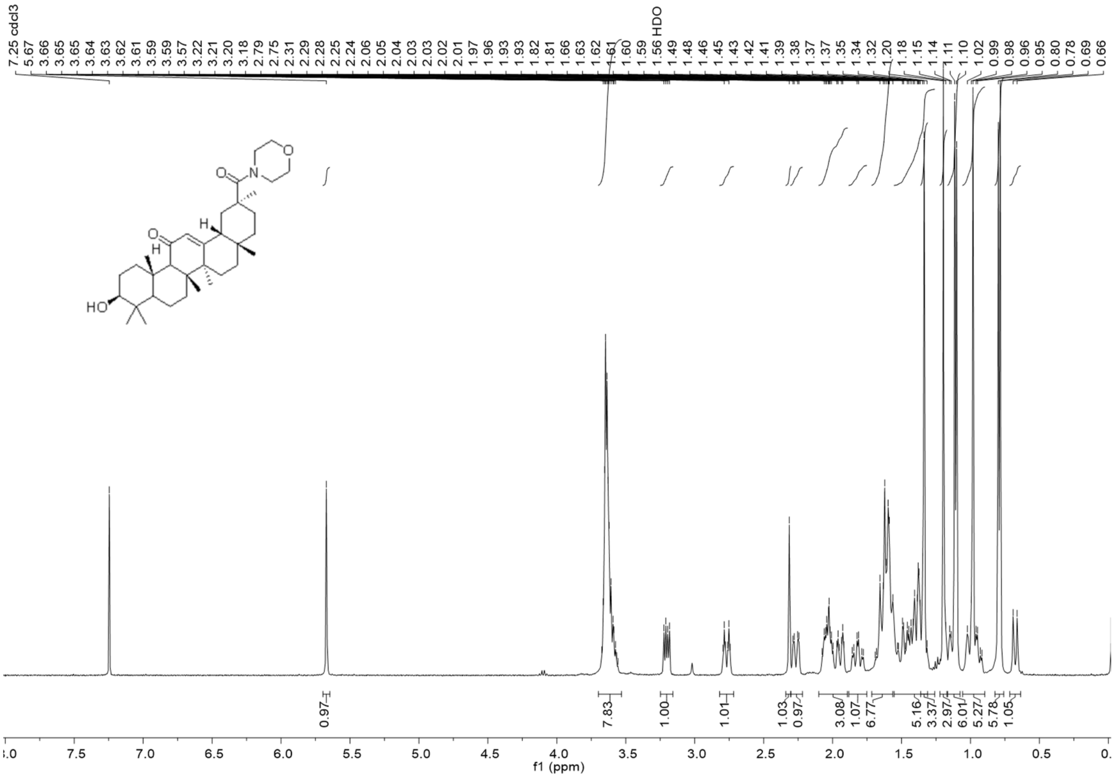

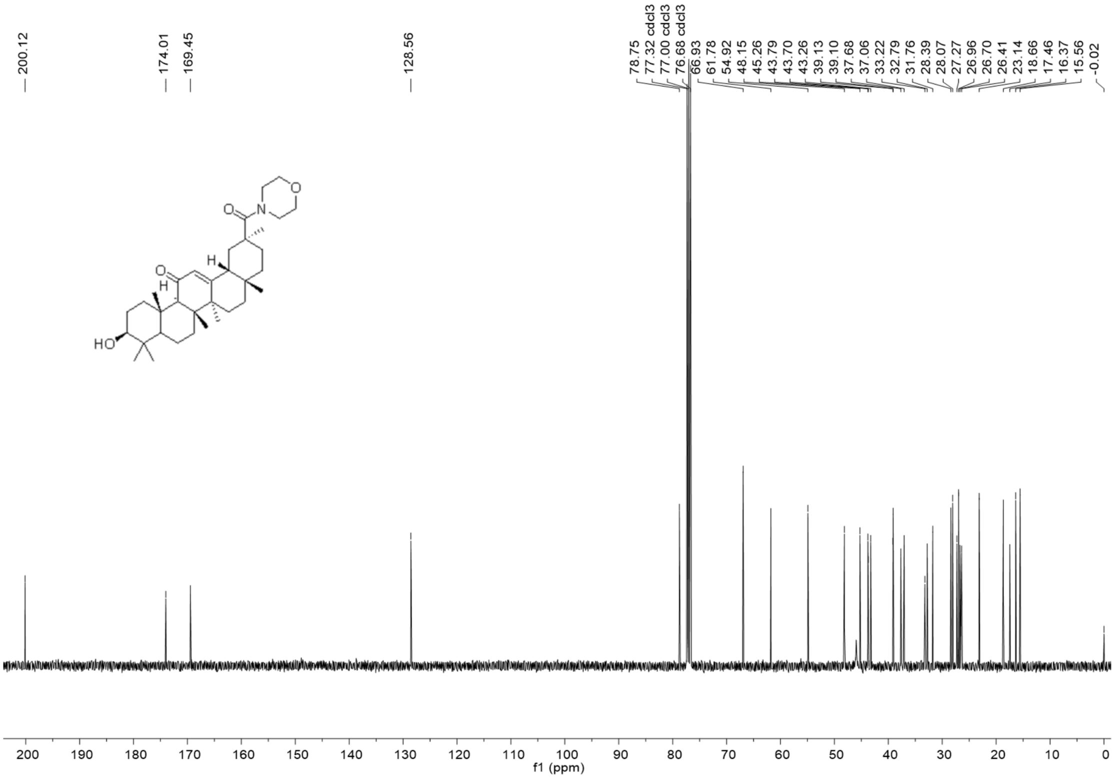

1H-benzo[d][1,2,3]triazol-1-yl 3β-hydroxy -11-oxo-olean-12-en-30-oate **2a**

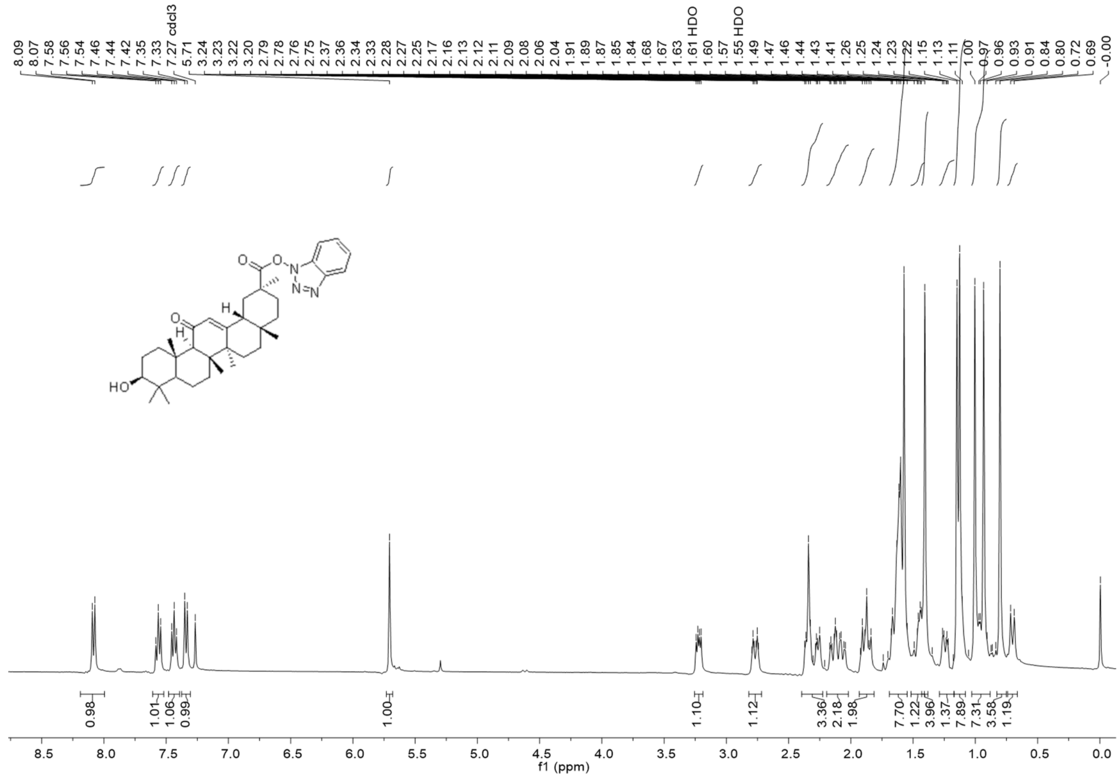

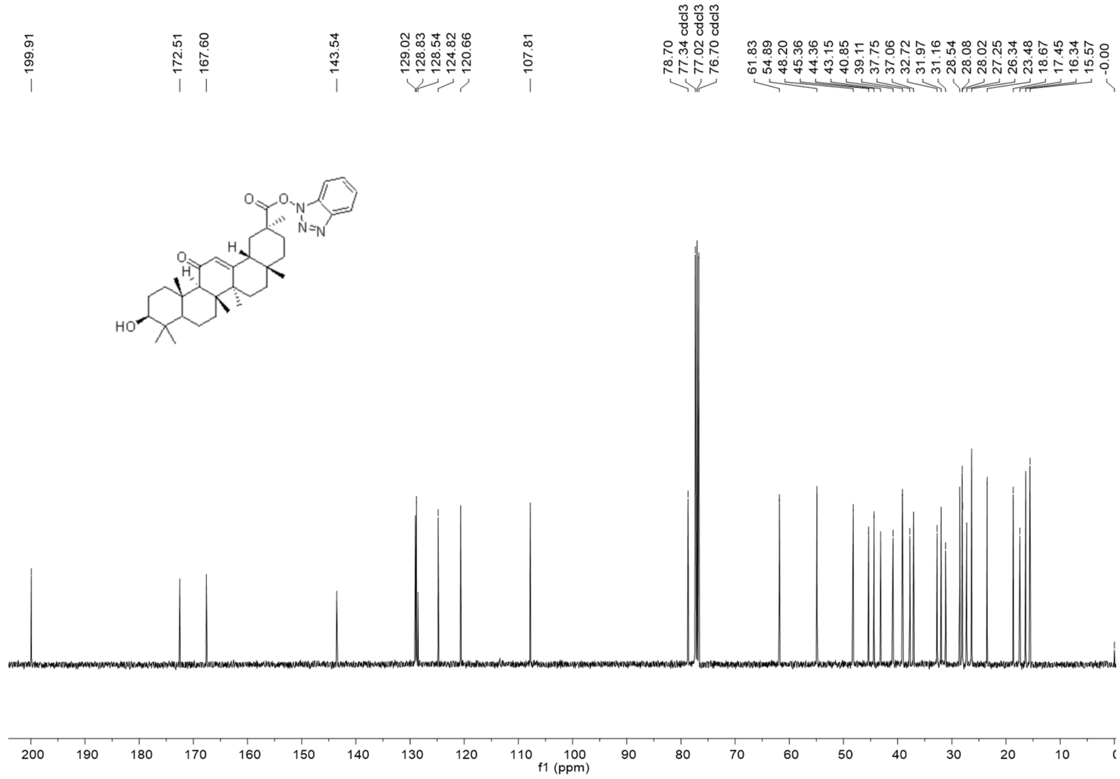

3*β*-(((3,4-dichlorophenyl)carbamoyl)oxy)-11-oxo-olean-12-en-30-oic acid **3a**

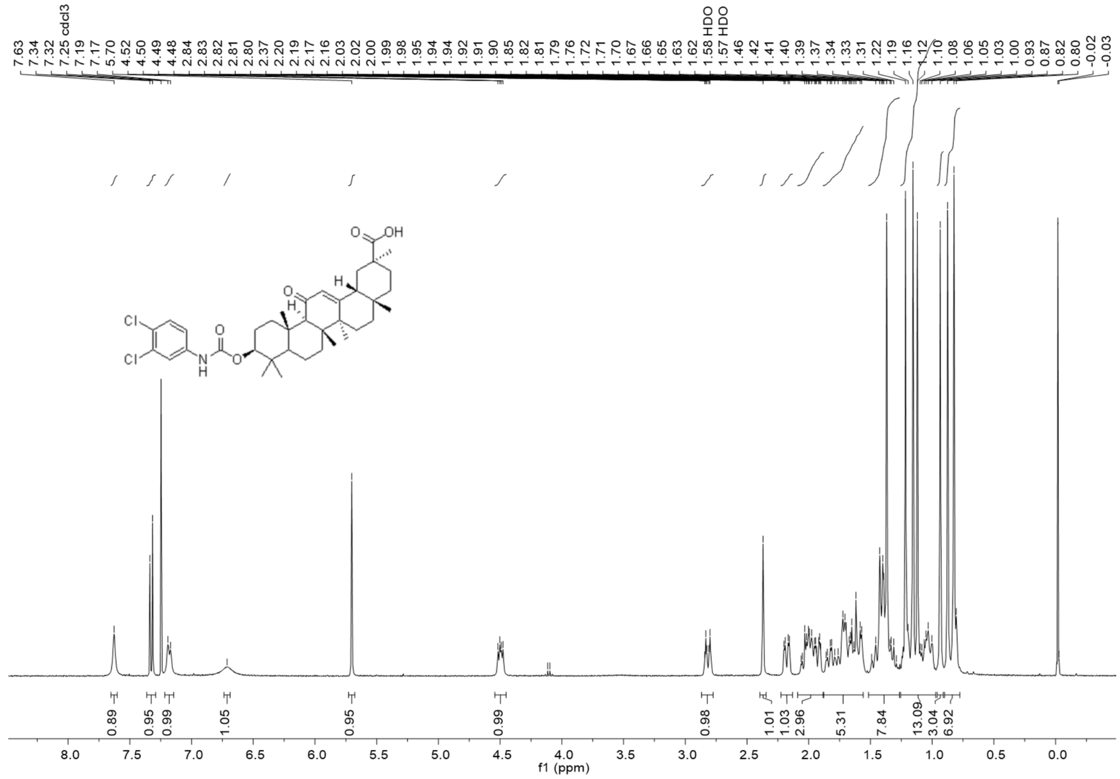

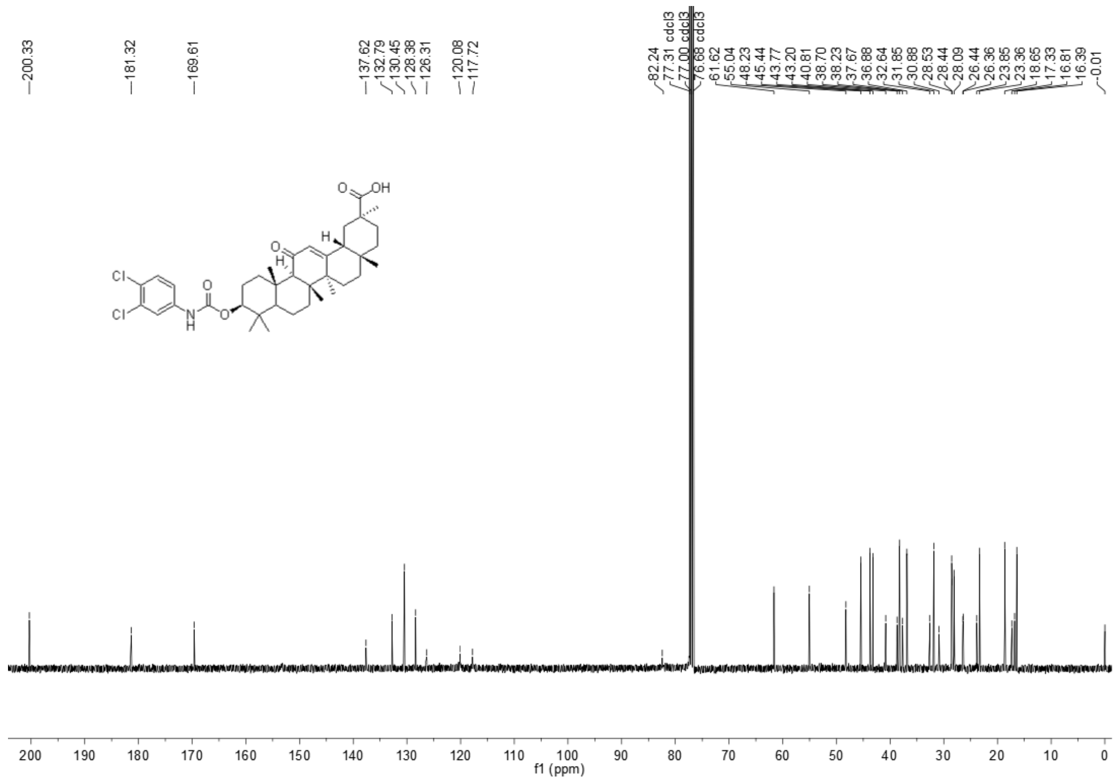

3*β*-(((4-chloro-3-(trifluoromethyl)phenyl)carbamoyl)oxy)-11-oxo-olean-12-en-30-oic acid **3b**

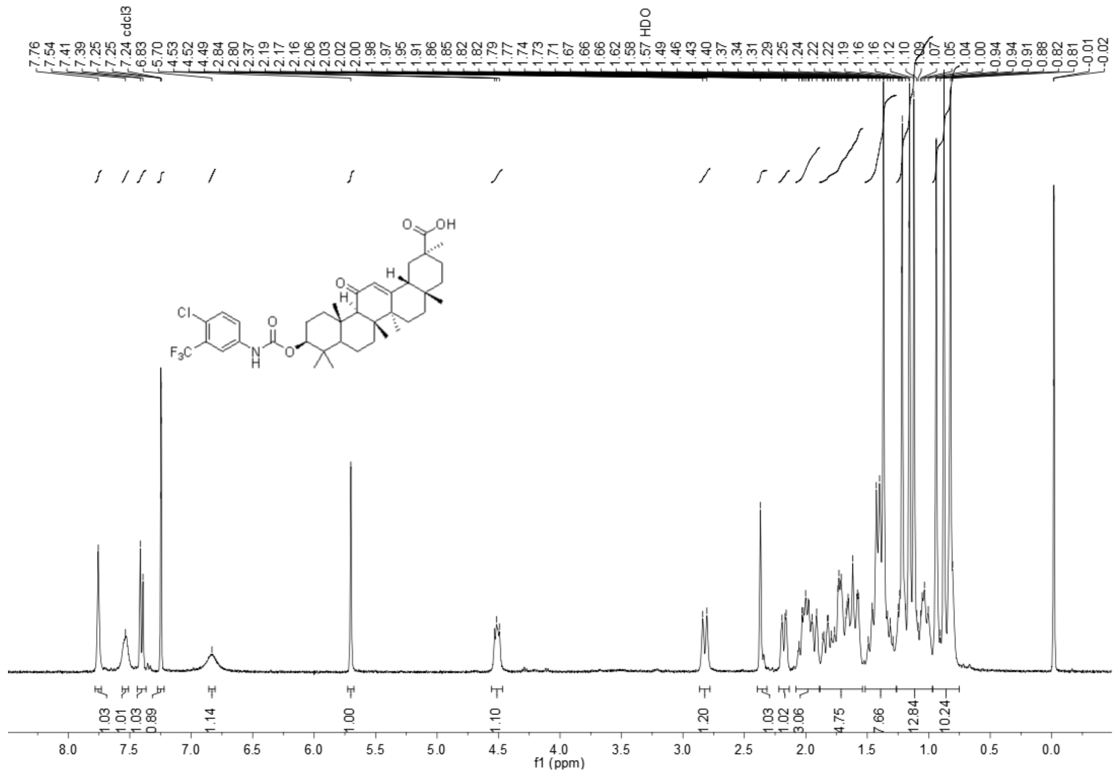

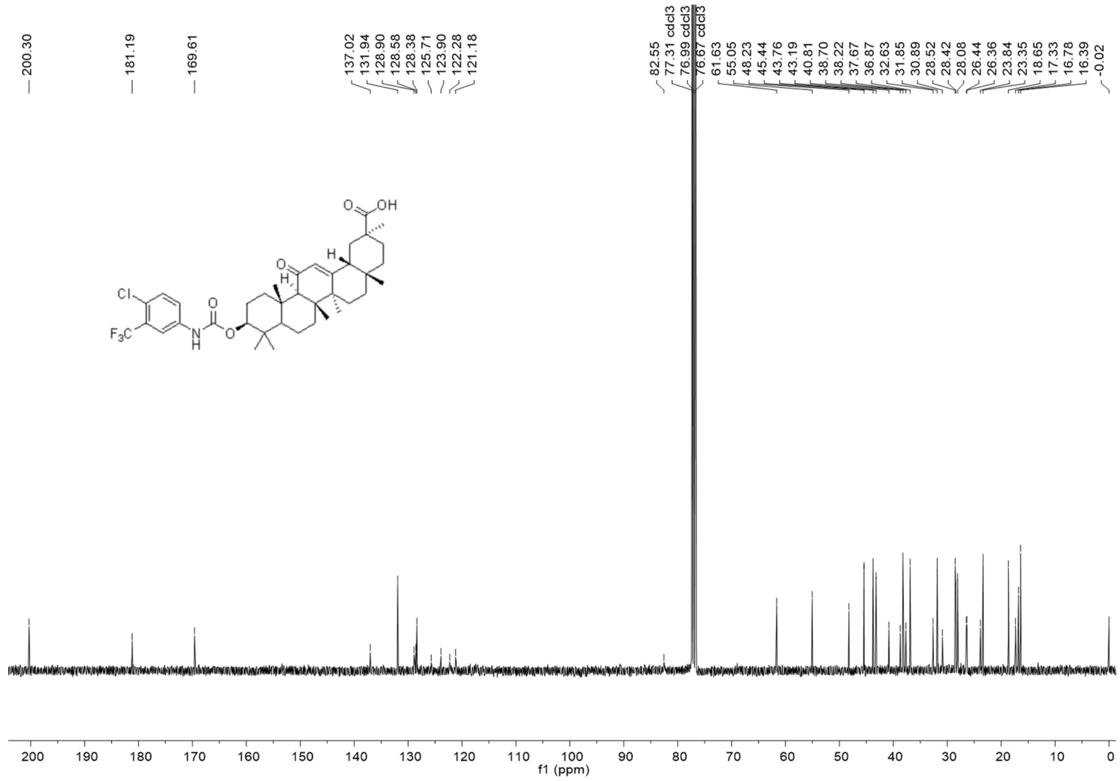

3*β*-(((3,5-dichlorophenyl)carbamoyl)oxy)-11-oxo-olean-12-en-30-oic acid **3c**

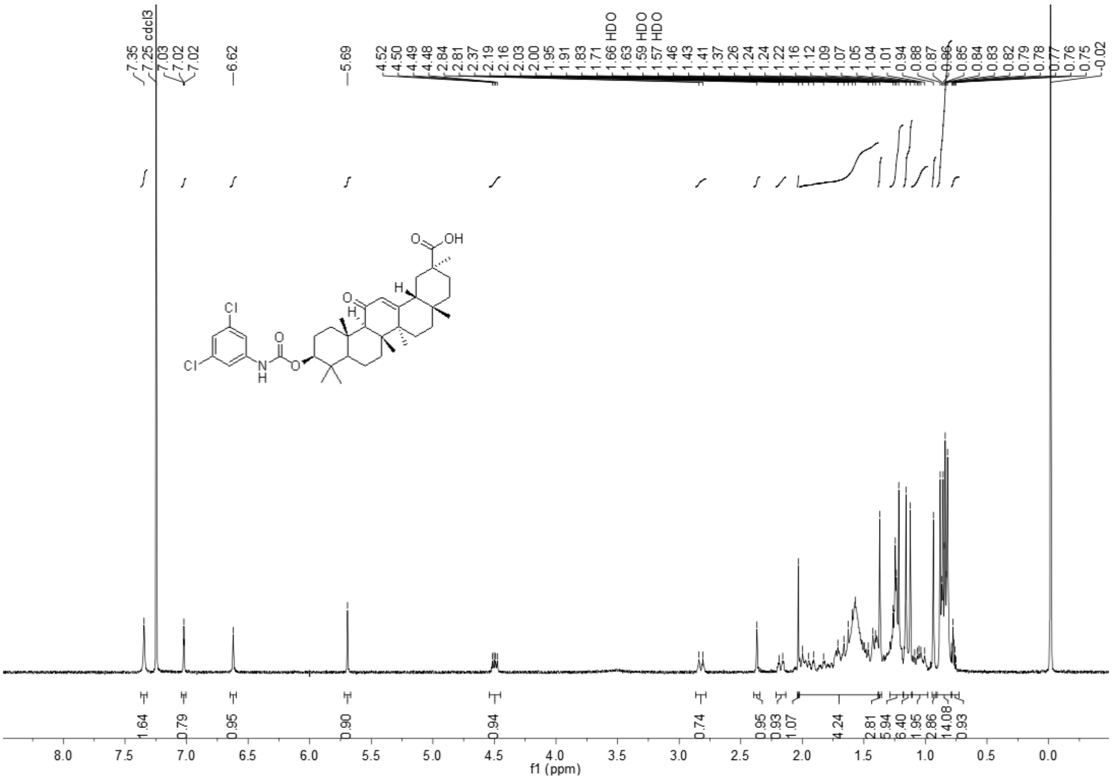

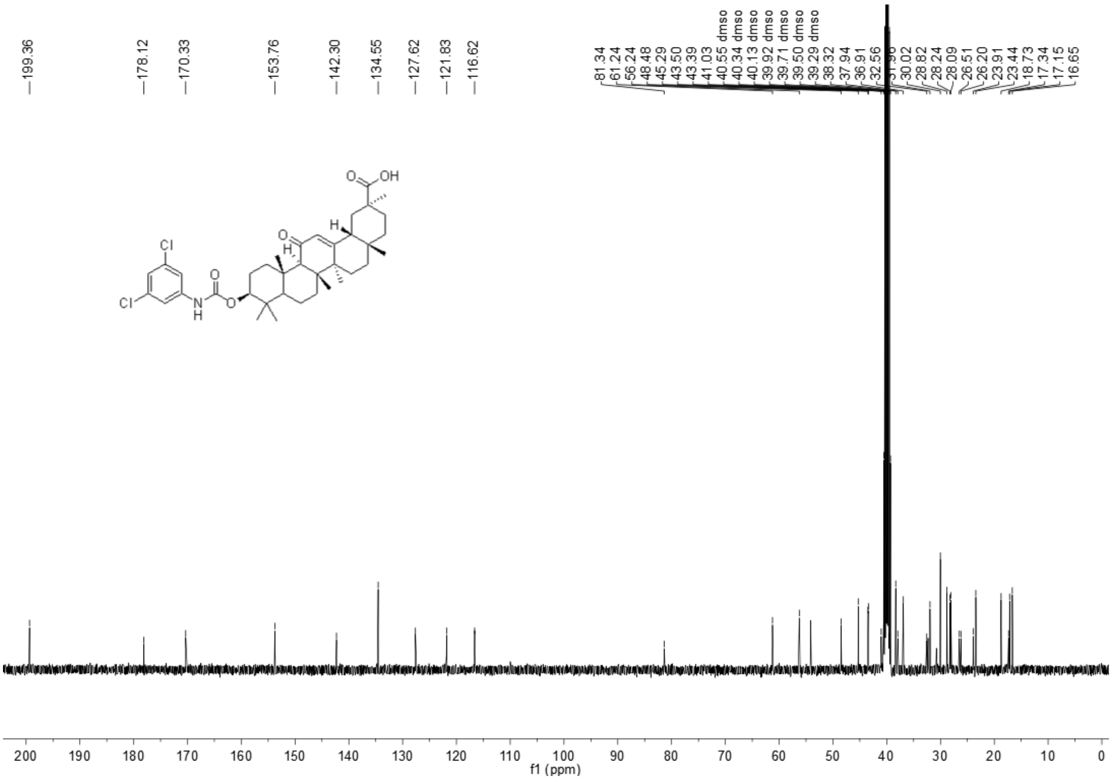

3*β*-(((4-chlorophenyl)carbamoyl)oxy)-11-oxo-olean-12-en-30-oic acid **3d**

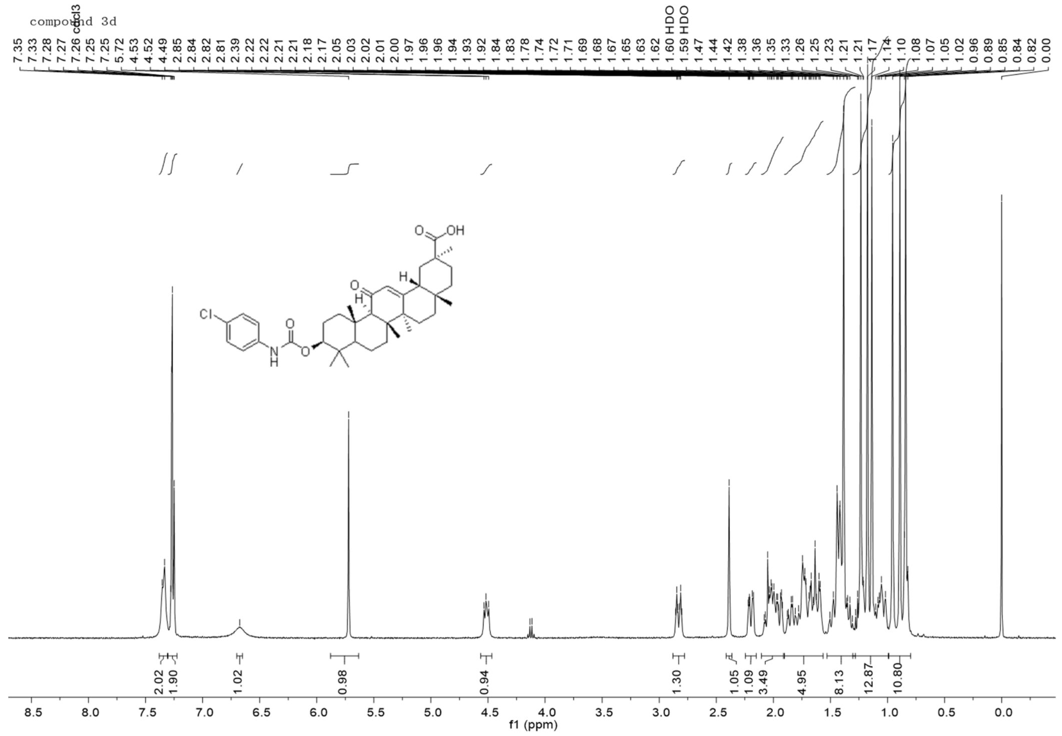

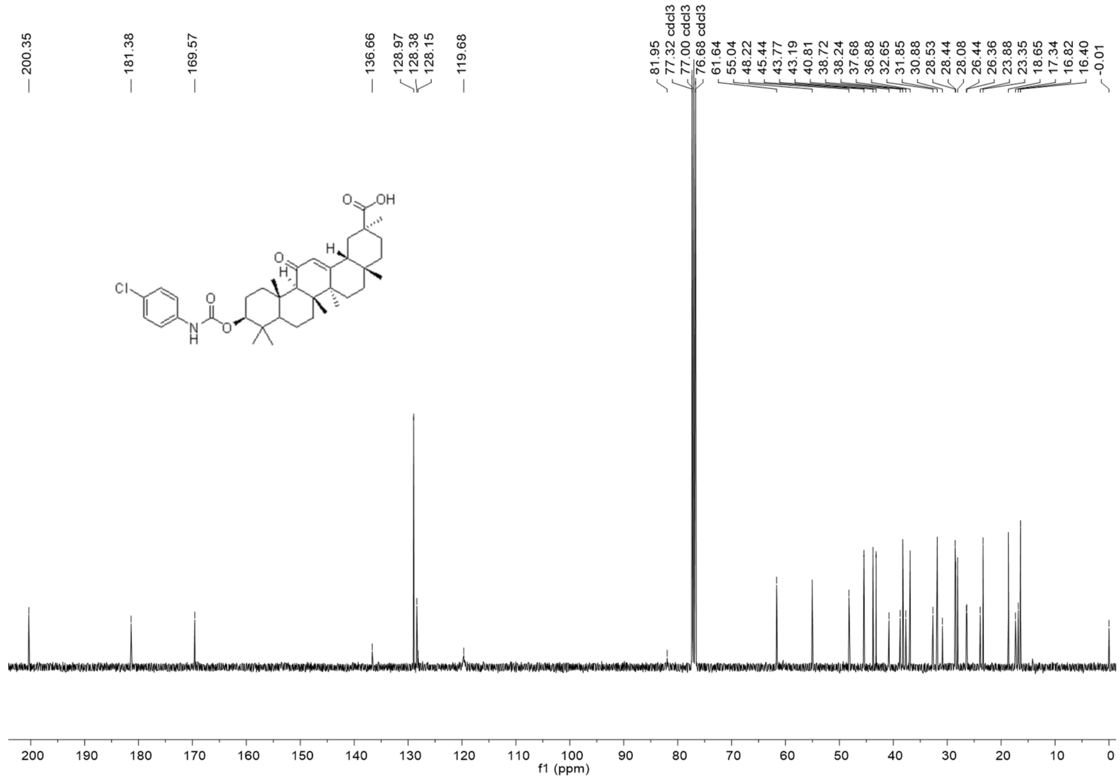

3*β*-(((3-chlorophenyl)carbamoyl)oxy)-11-oxo-olean-12-en-30-oic acid **3e**

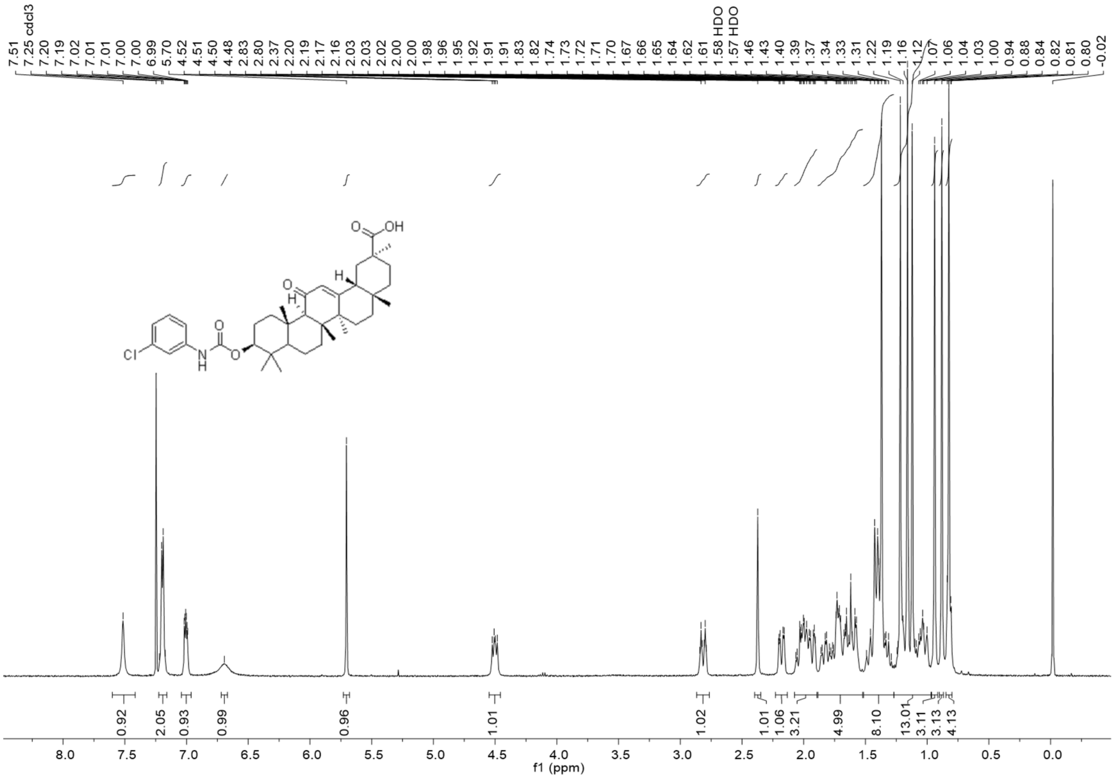

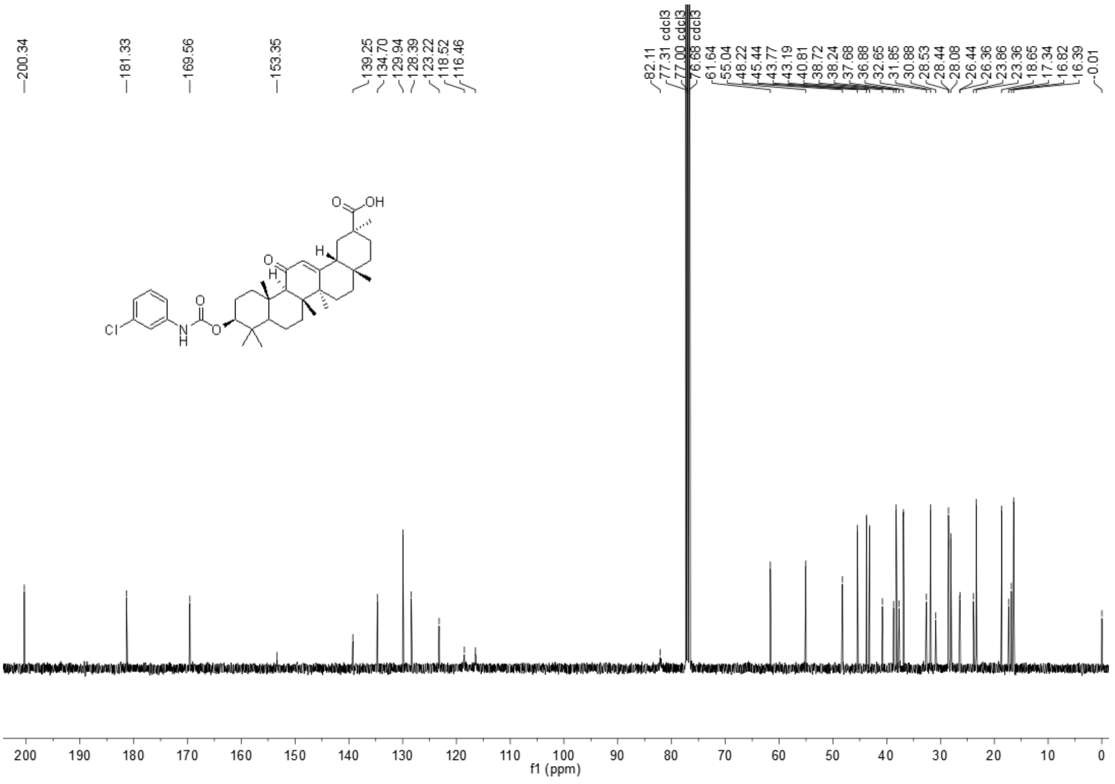

3*β*-(((3-chloro-4-methylphenyl)carbamoyl)oxy)-11-oxo-olean-12-en-30-oic acid **3f**

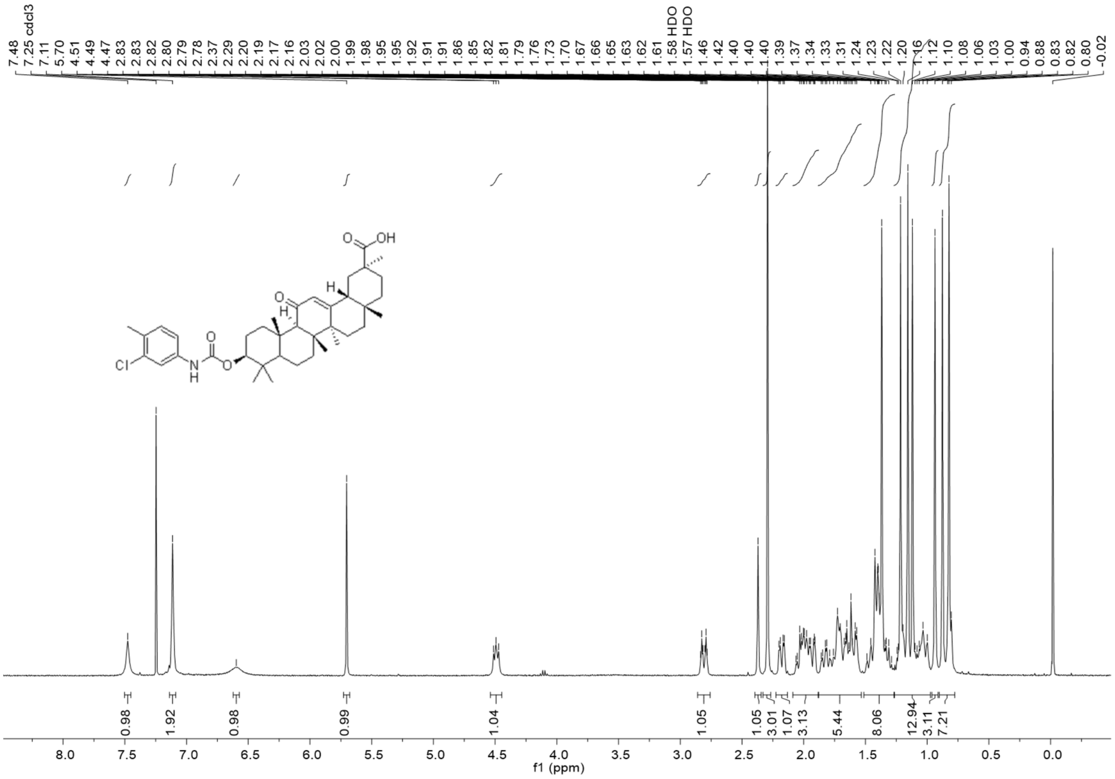

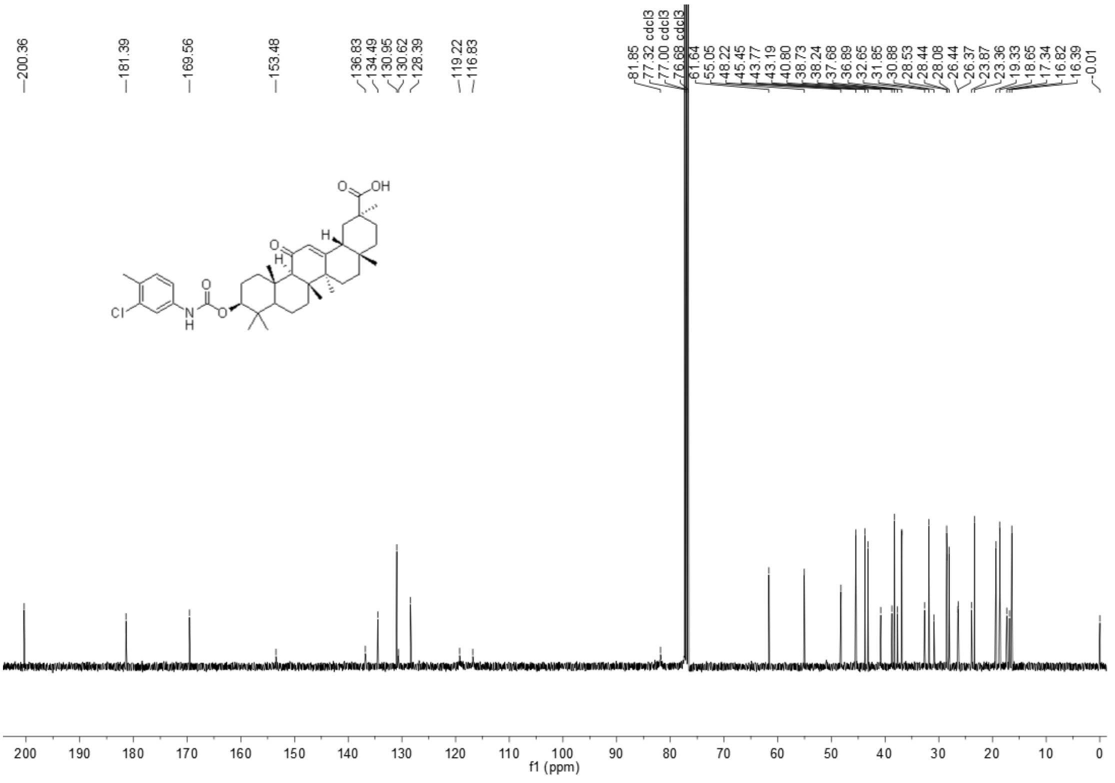

3*β*-(((4-bromophenyl)carbamoyl)oxy)-11-oxo-olean-12-en-30-oic acid **3g**

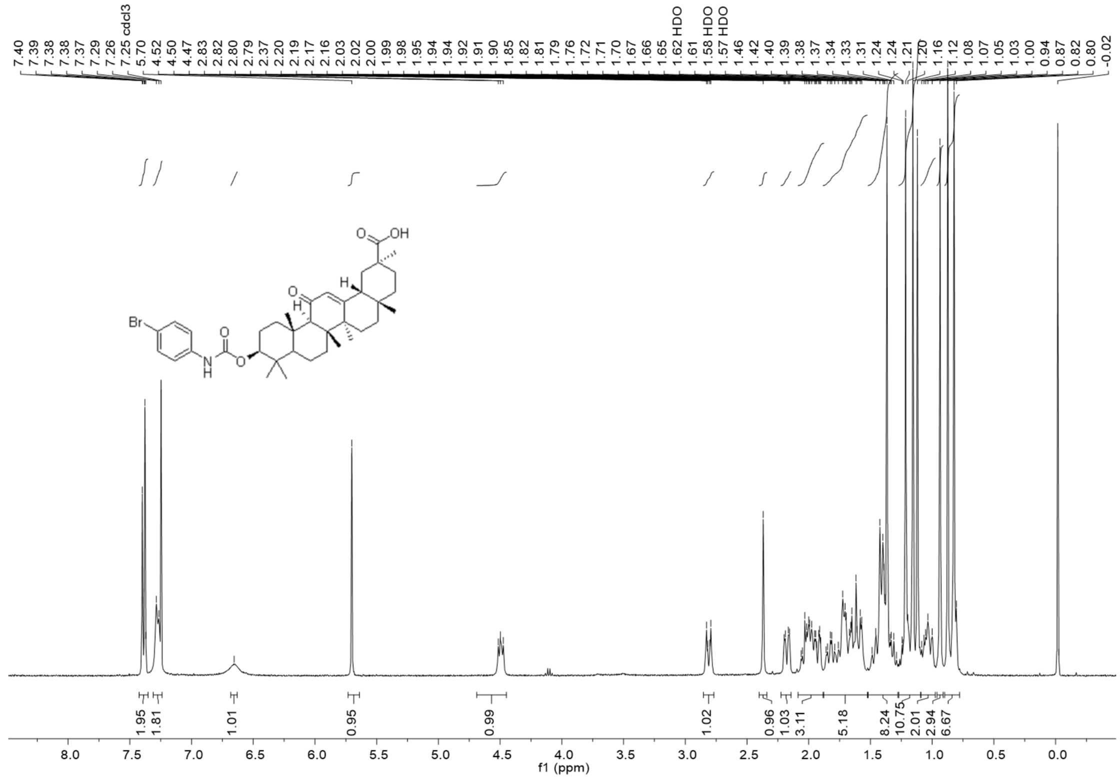

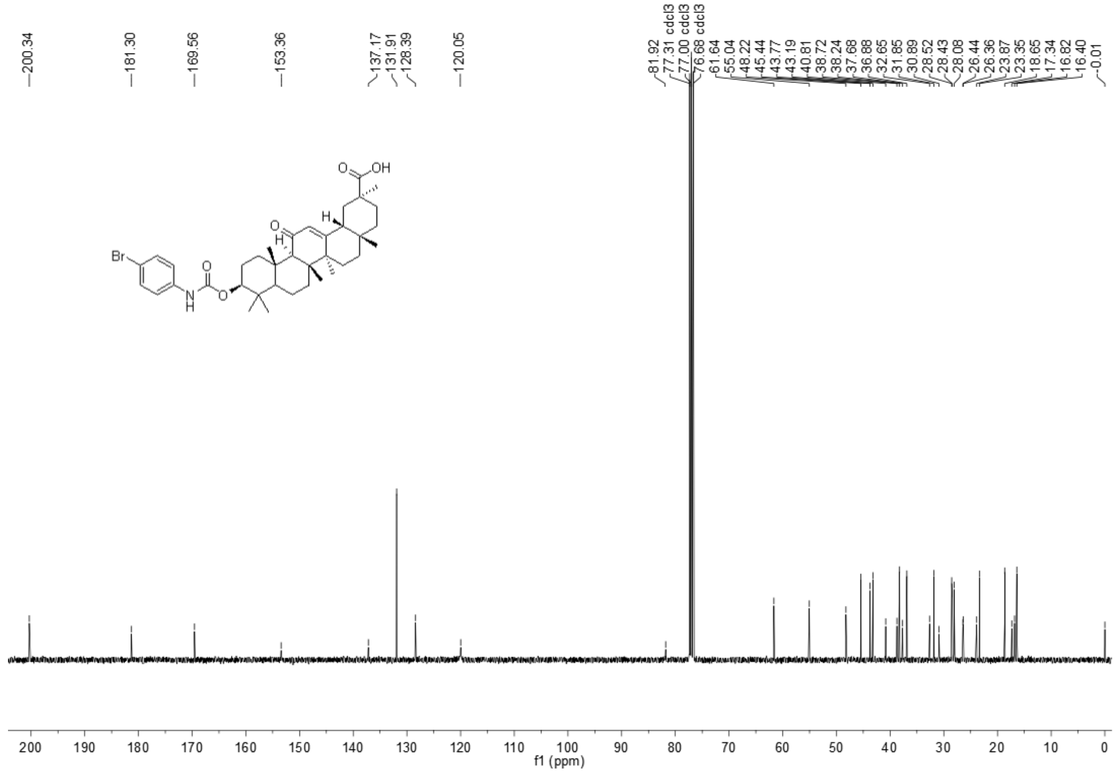

3*β*-(((4-fluorophenyl)carbamoyl)oxy)-11-oxo-olean-12-en-30-oic acid **3h**

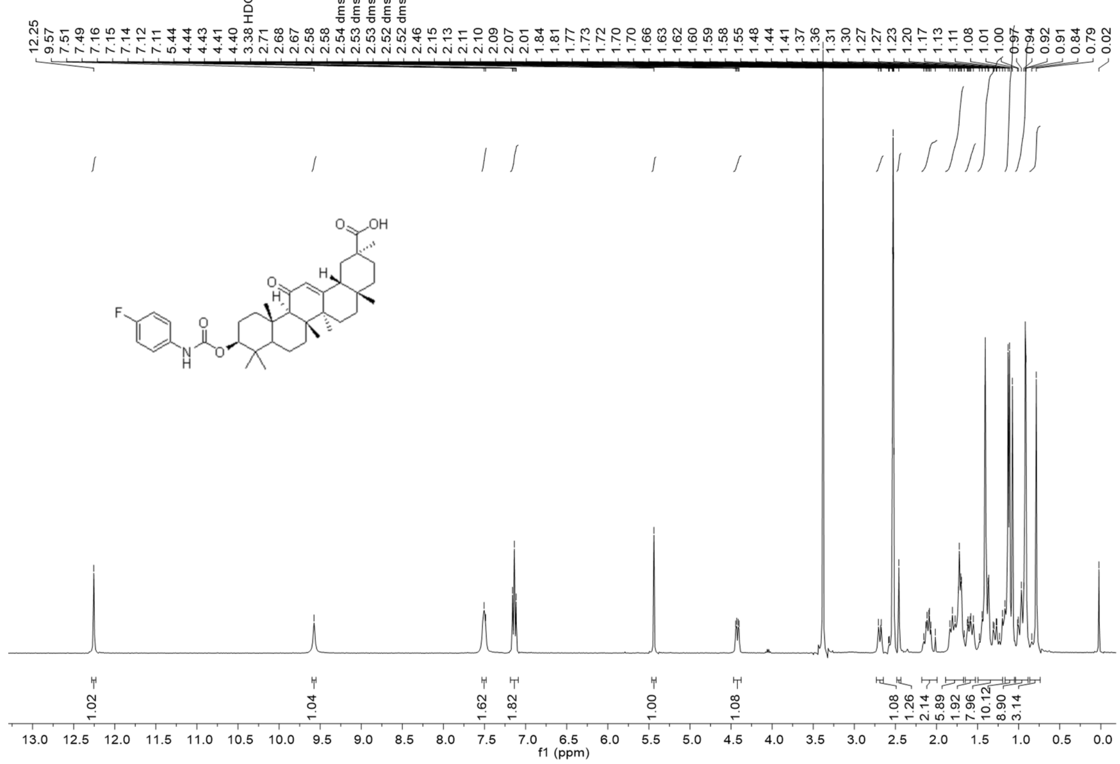

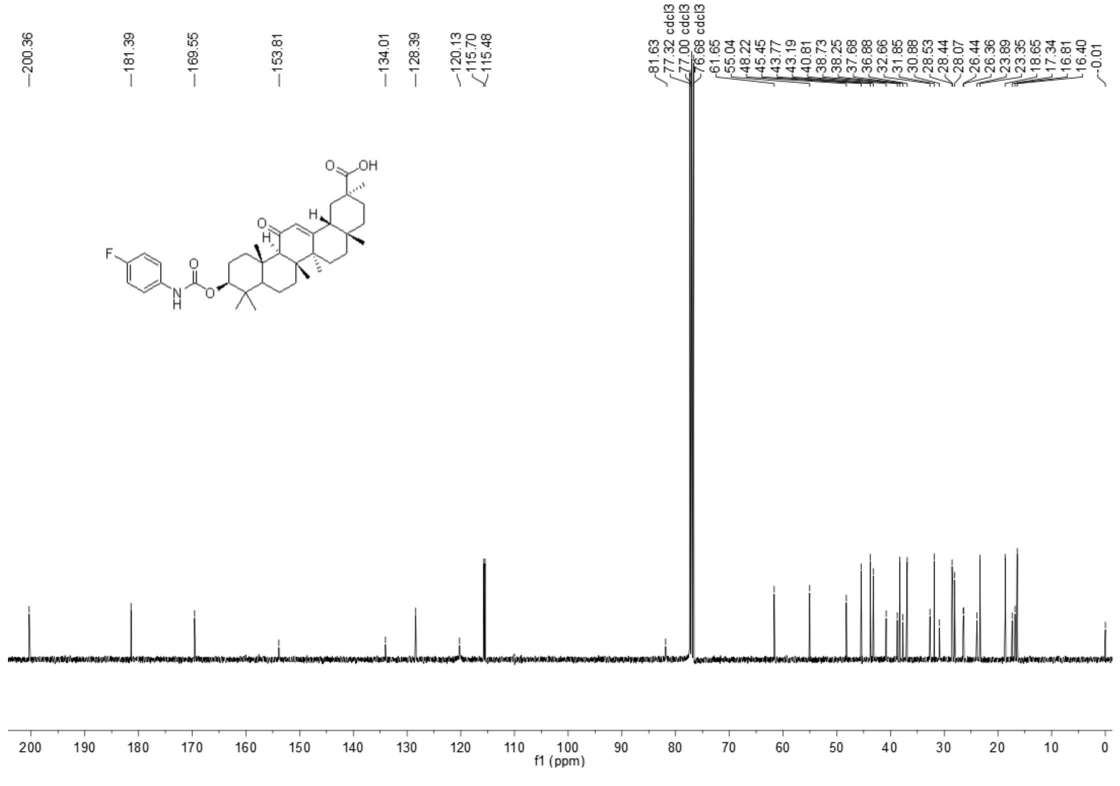

3*β*-(((4- (trifluoromethyl)phenyl)carbamoyl)oxy)-11-oxo-olean-12-en-30-oic acid **3i**

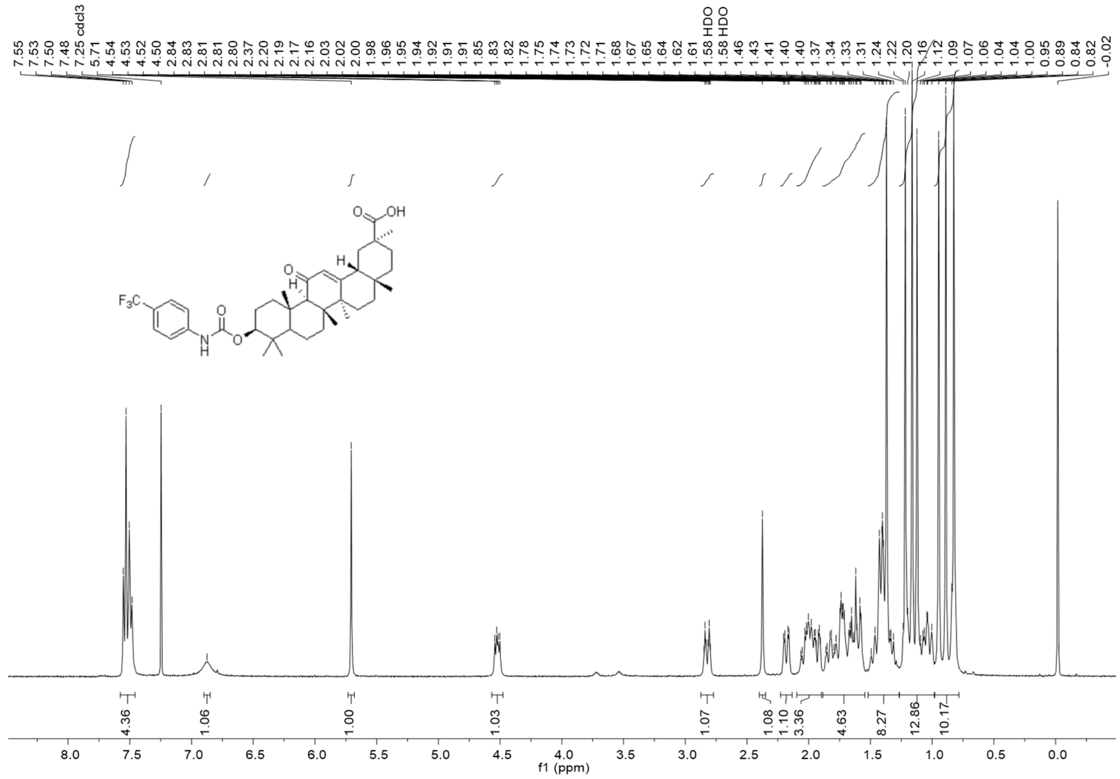

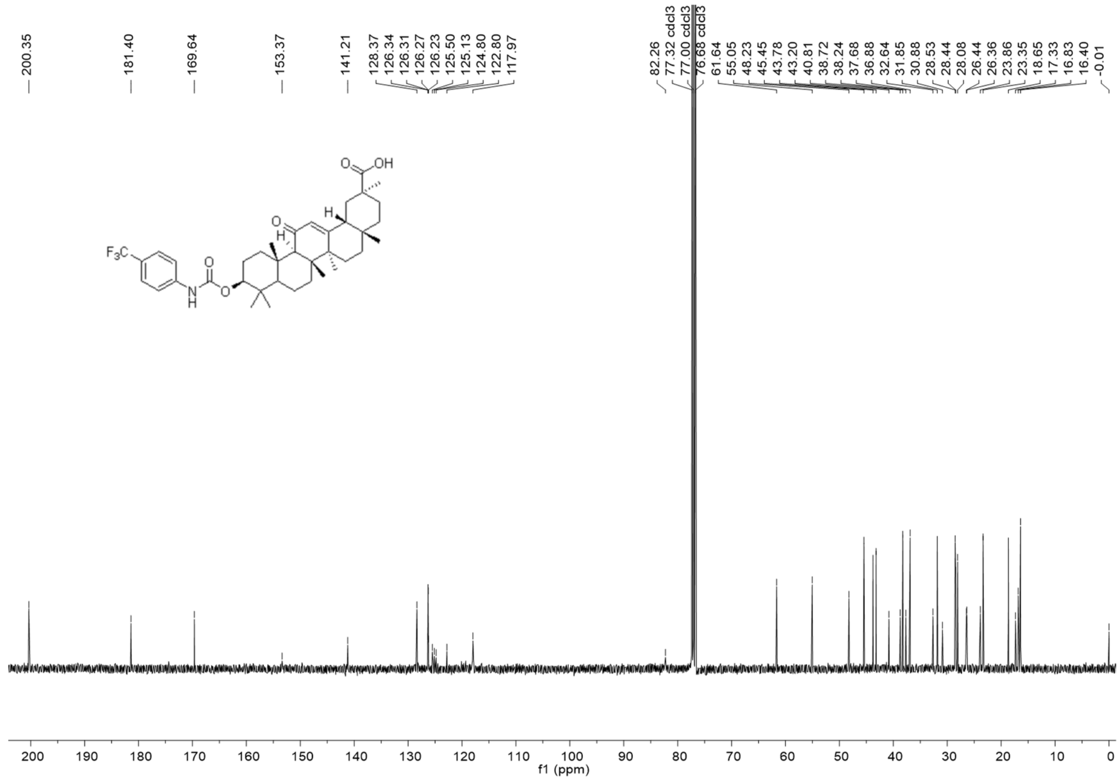

3*β*-(((3- (trifluoromethyl)phenyl)carbamoyl)oxy)-11-oxo-olean-12-en-30-oic acid **3j**

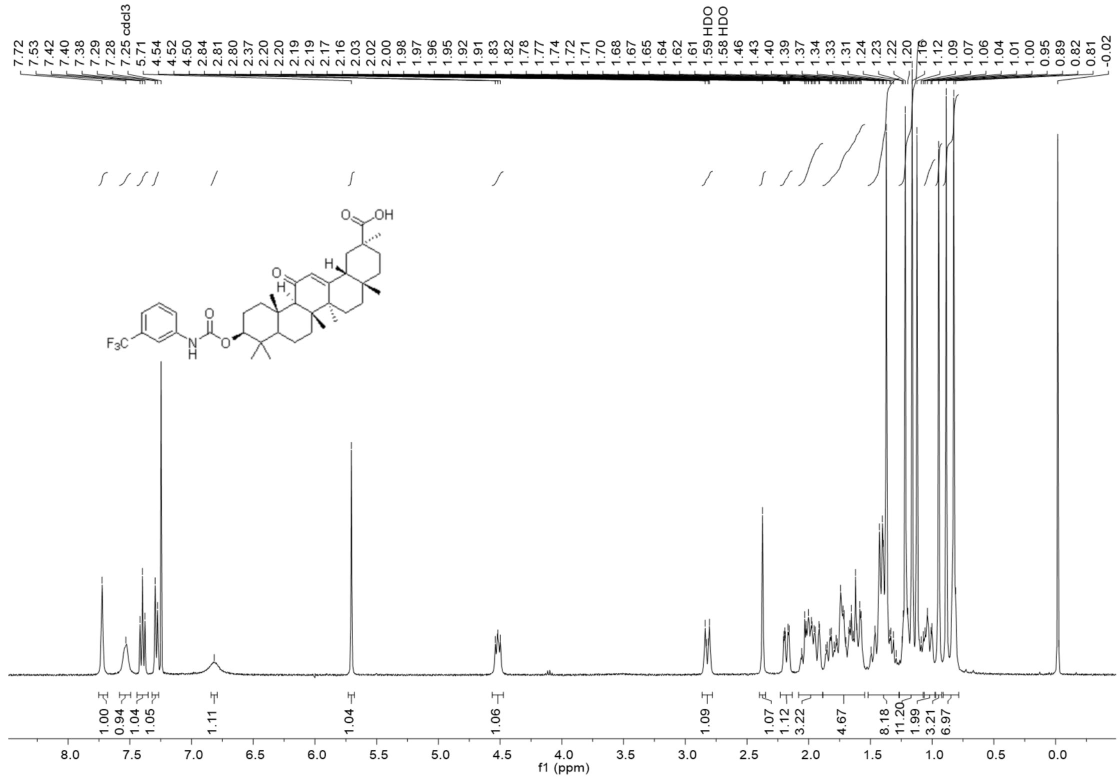

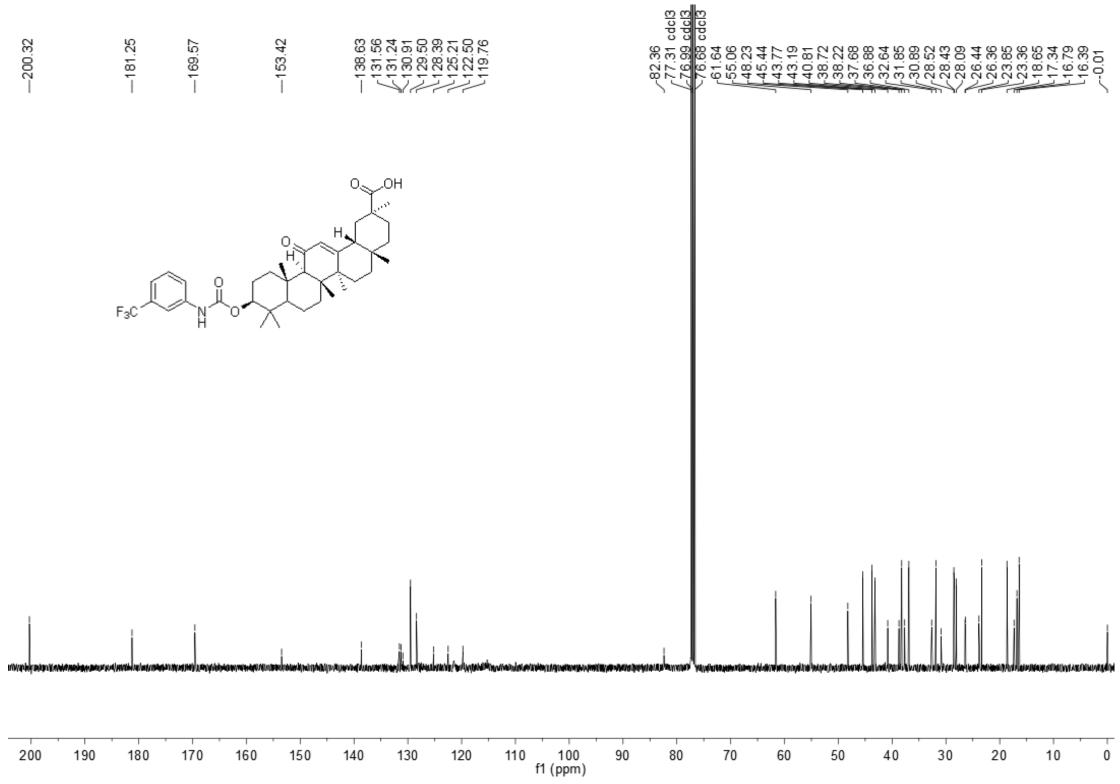

3*β*-(((3,5-bis (trifluoromethyl)phenyl)carbamoyl)oxy)-11-oxo-olean-12-en-30-oic acid **3k**

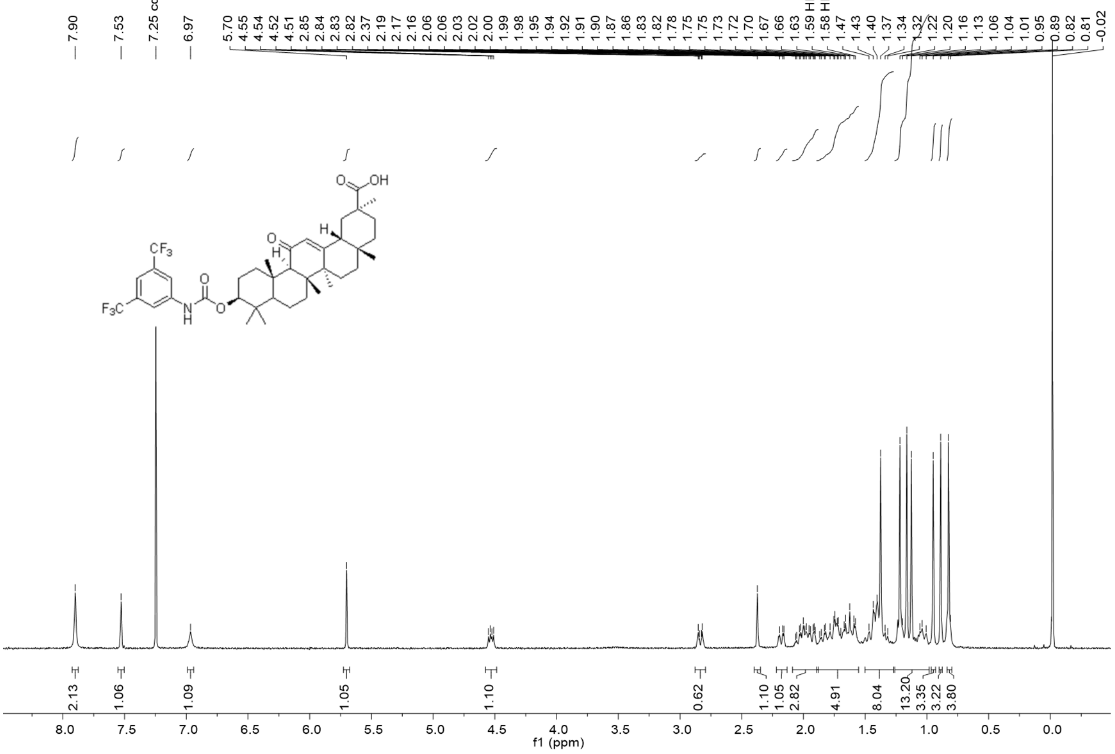

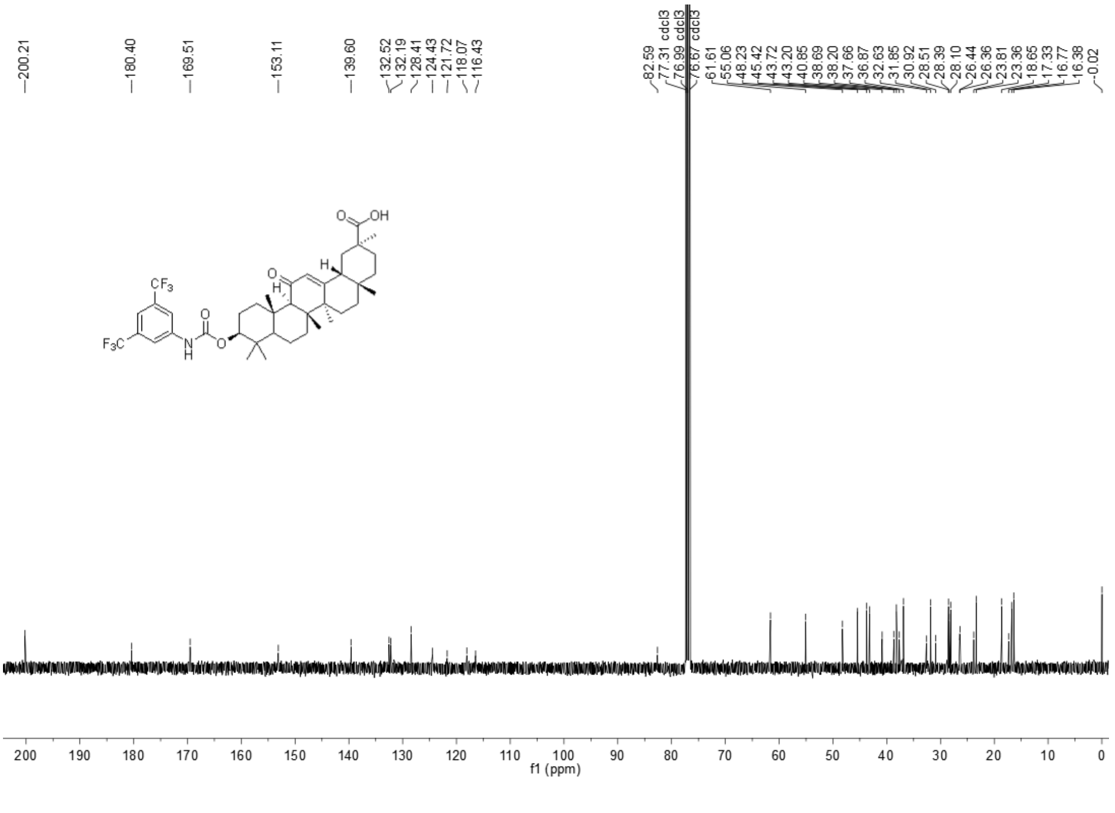

3*β*-(((3-methoxyphenyl)carbamoyl)oxy)-11-oxo-olean-12-en-30-oic acid **3l**

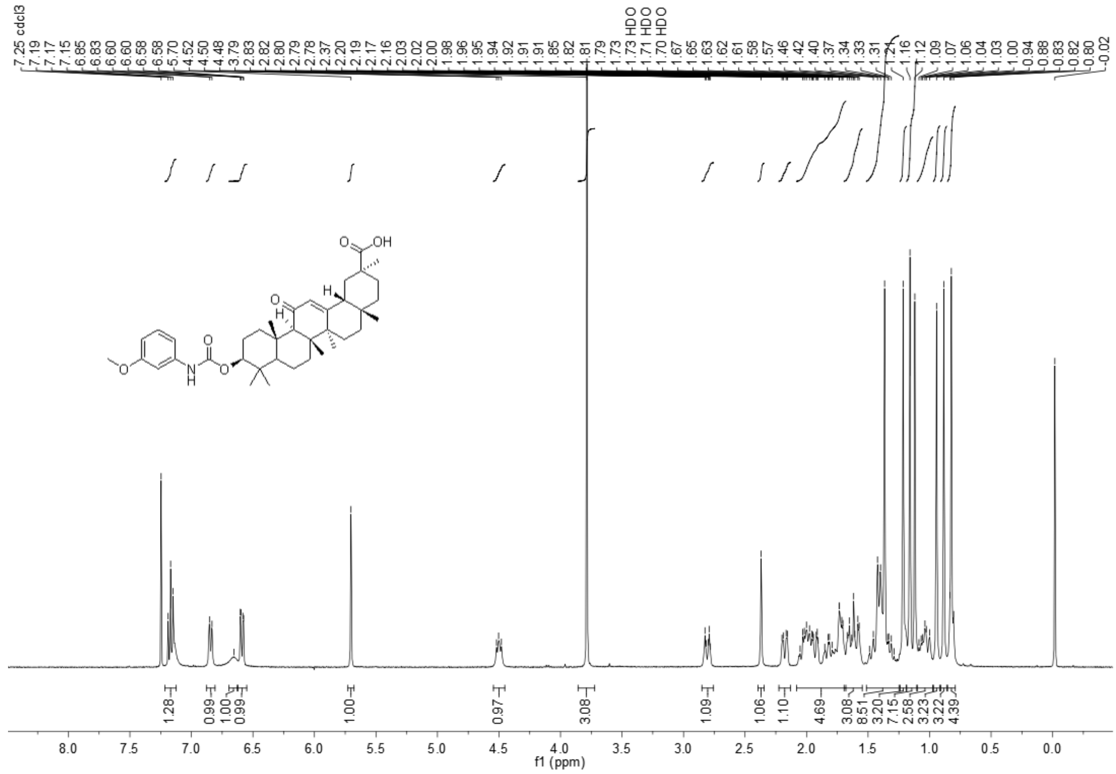

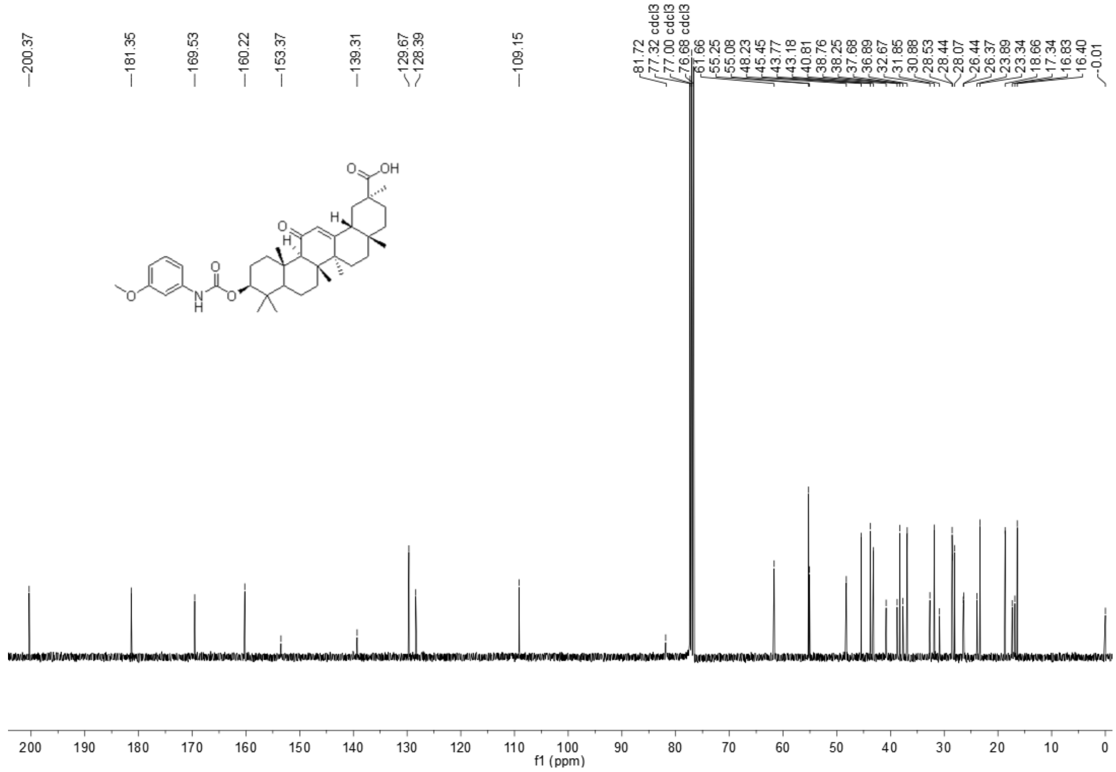

3*β*-(((4-methoxyphenyl)carbamoyl)oxy)-11-oxo-olean-12-en-30-oic acid **3m**

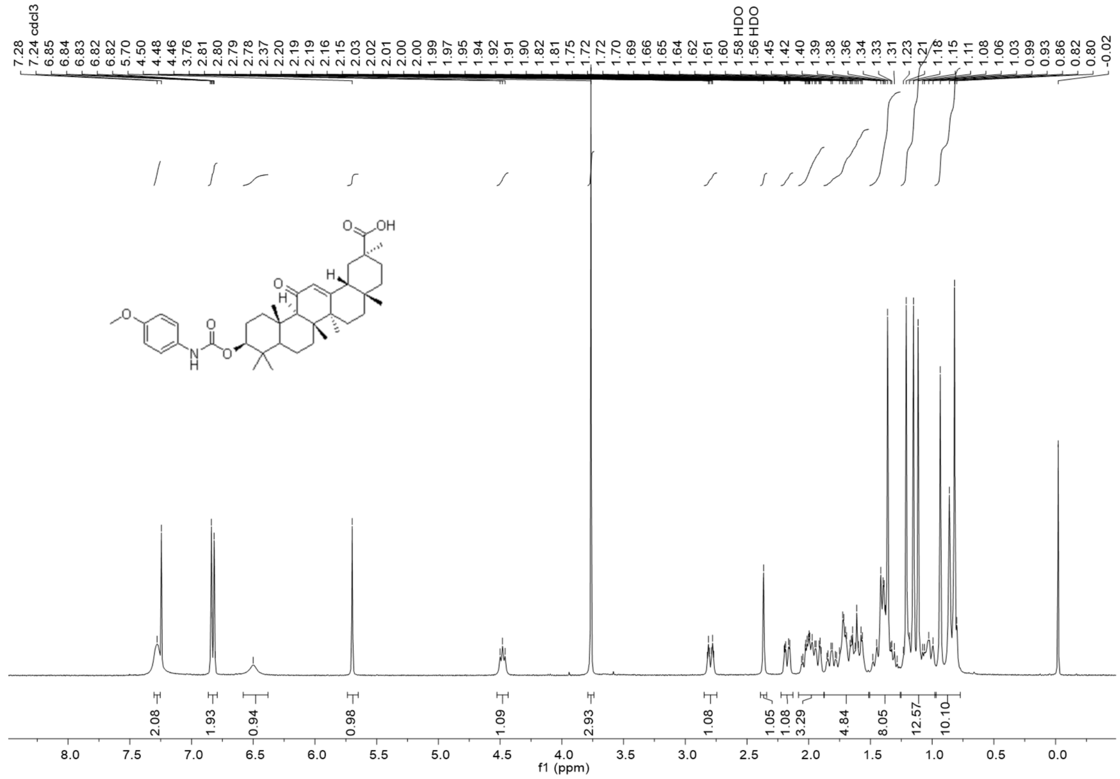

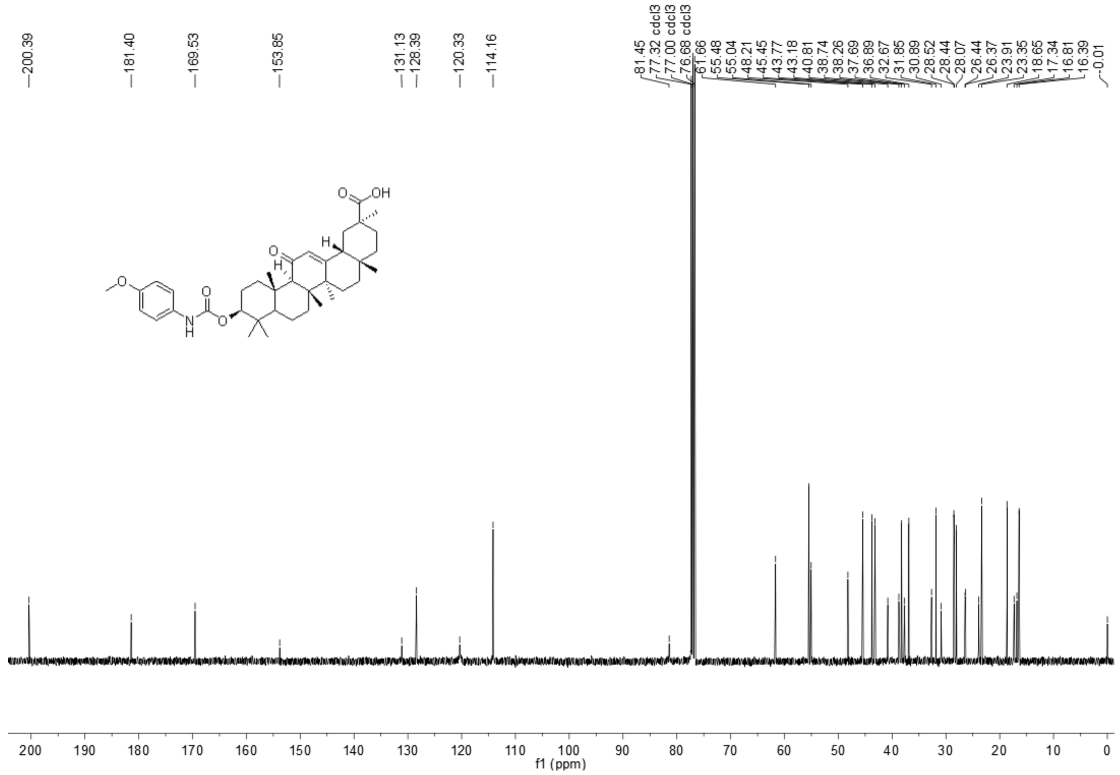

3*β*-(((4-(trifluoromethoxy)phenyl)carbamoyl)oxy)-11-oxo-olean-12-en-30-oic acid **3n**

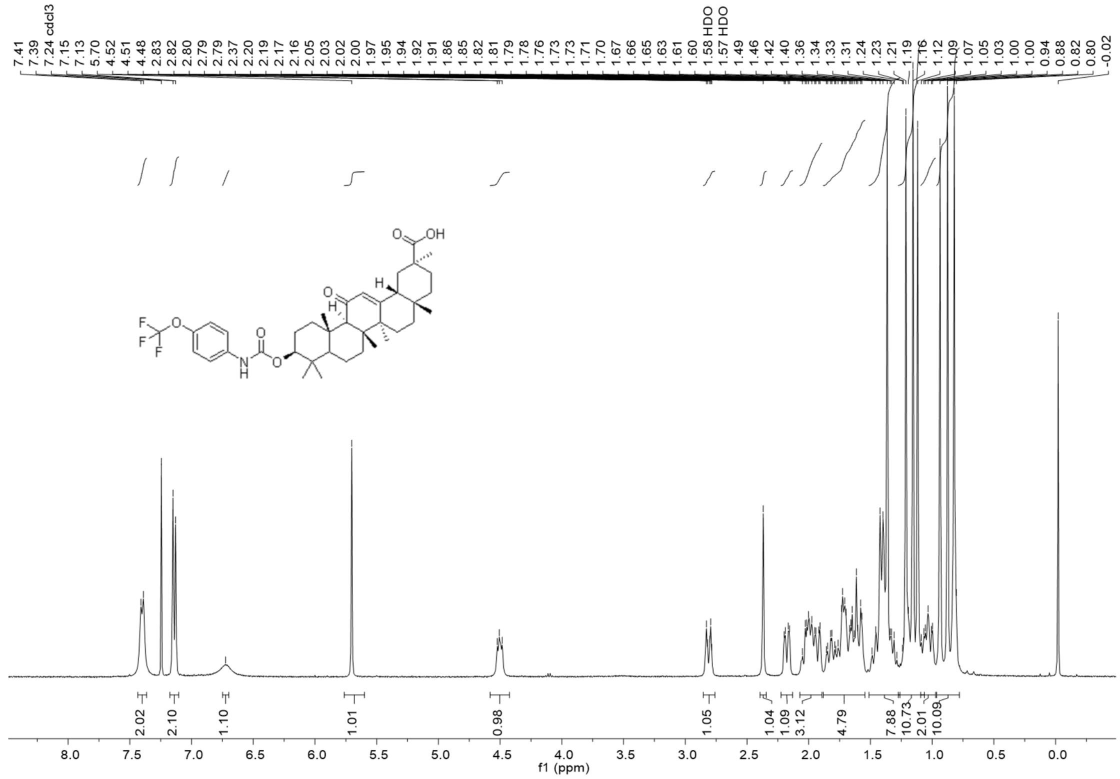

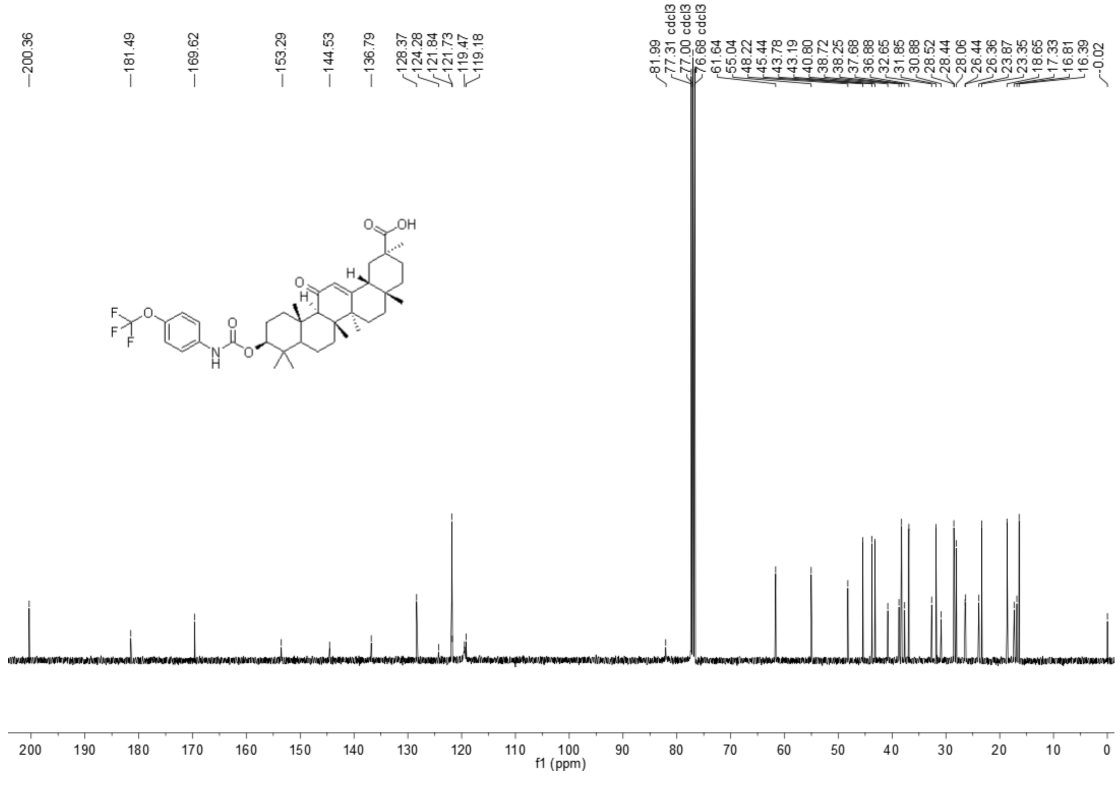

3*β*-(((3,5-dimethylphenyl)carbamoyl)oxy)-11-oxo-olean-12-en-30-oic acid **3o**

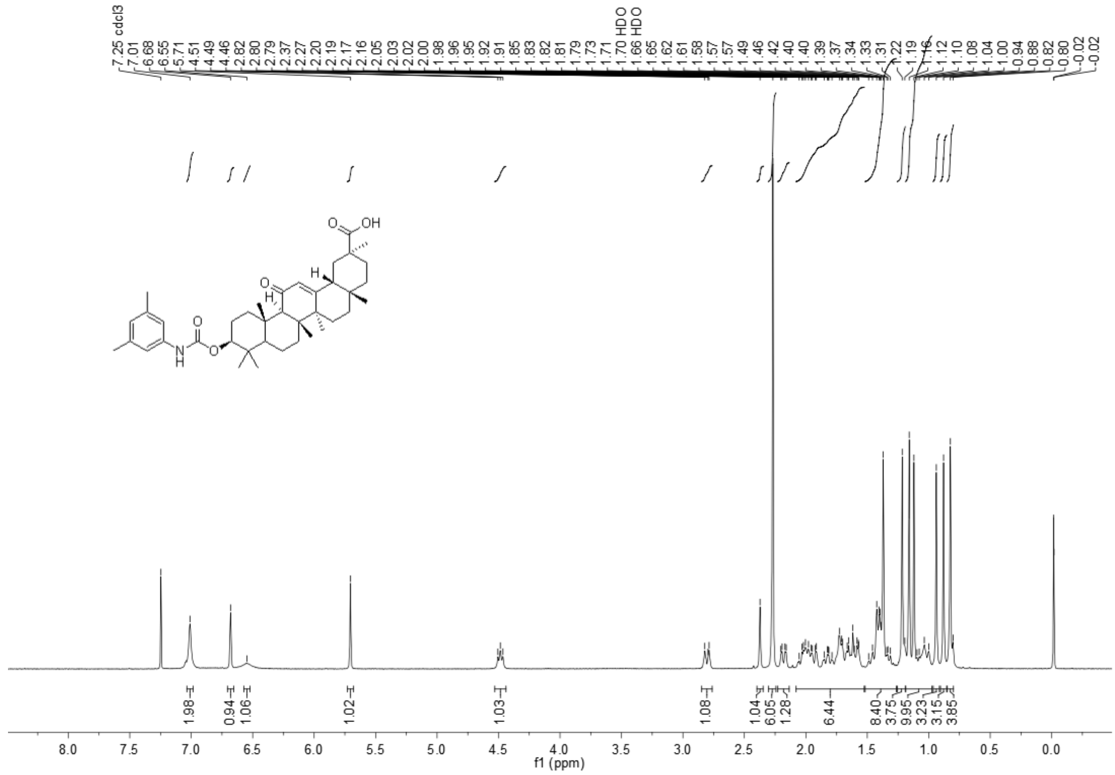

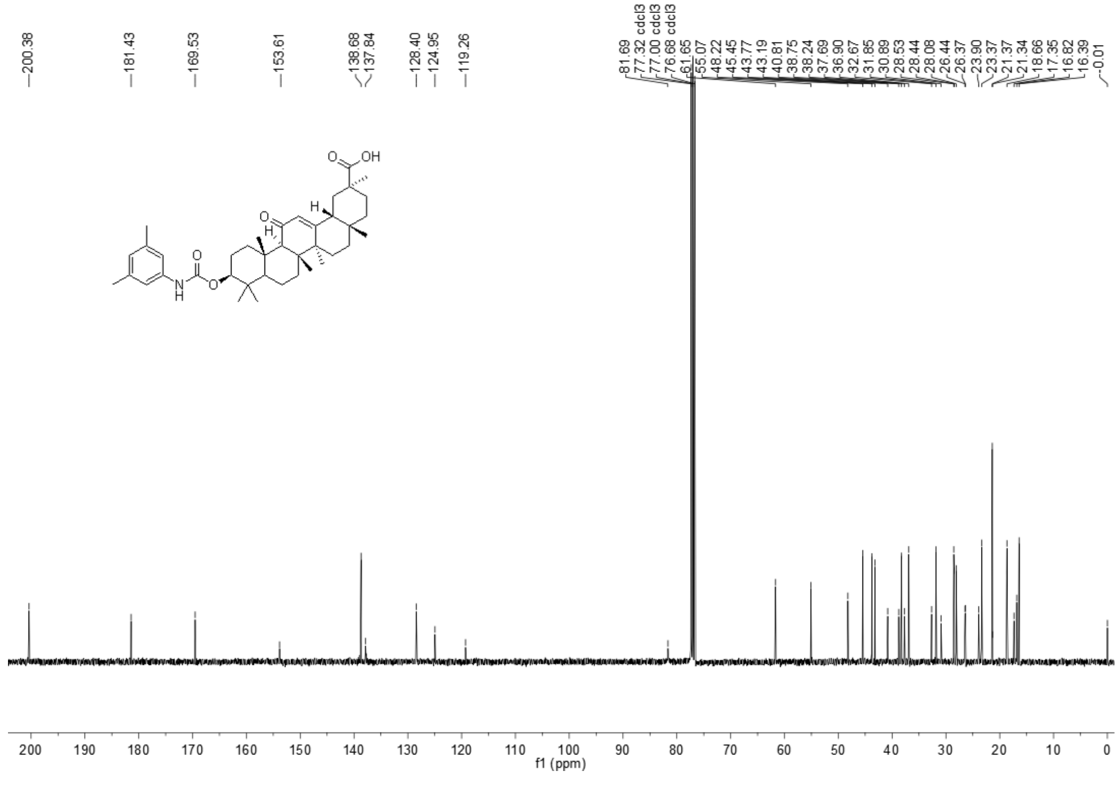

3*β*-(((3,4-dichlorophenyl)carbamoyl)oxy)-30-morpholino-olean-12-ene-11,30-dione **4a**

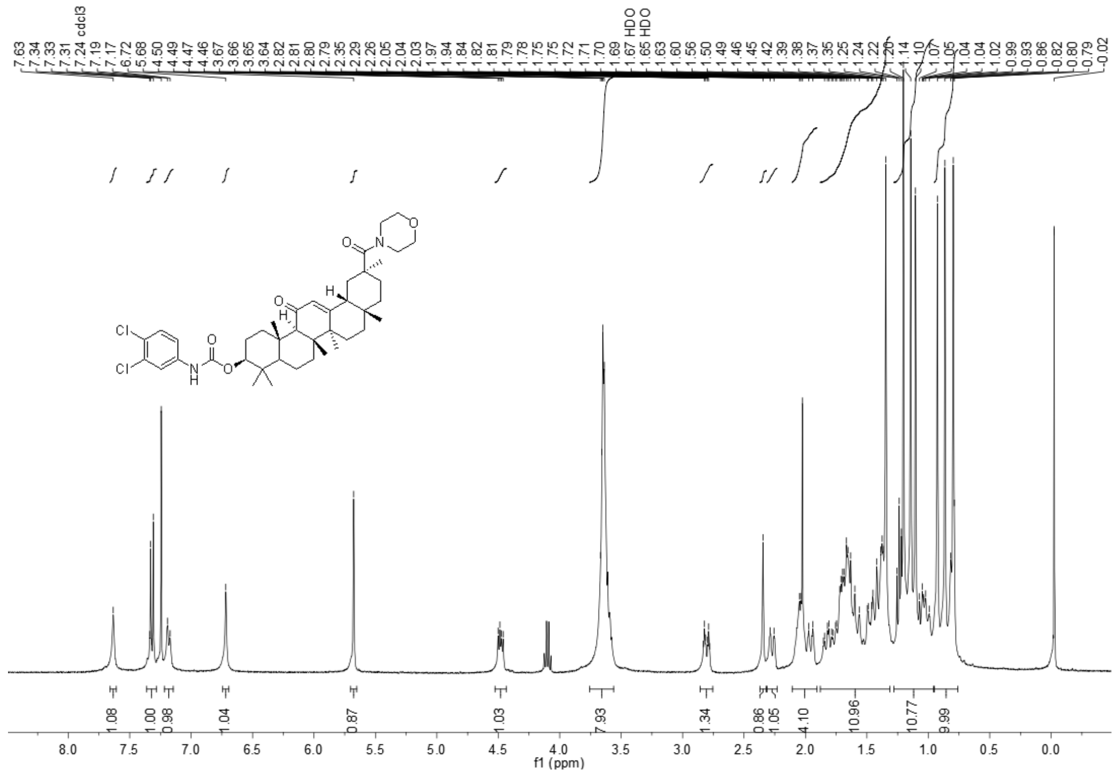

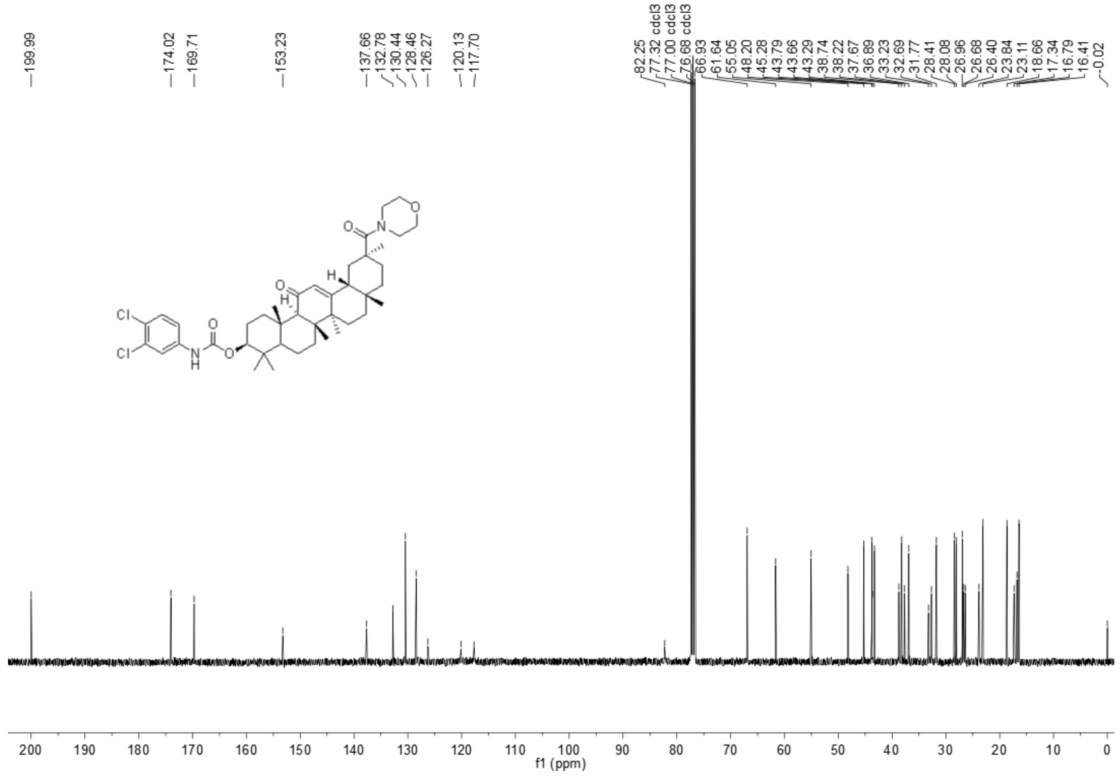

3*β*-(((4-chloro-3-(trifluoromethyl)phenyl)carbamoyl)oxy)-30-morpholino-olean-12-ene-11,30-dione **4b**

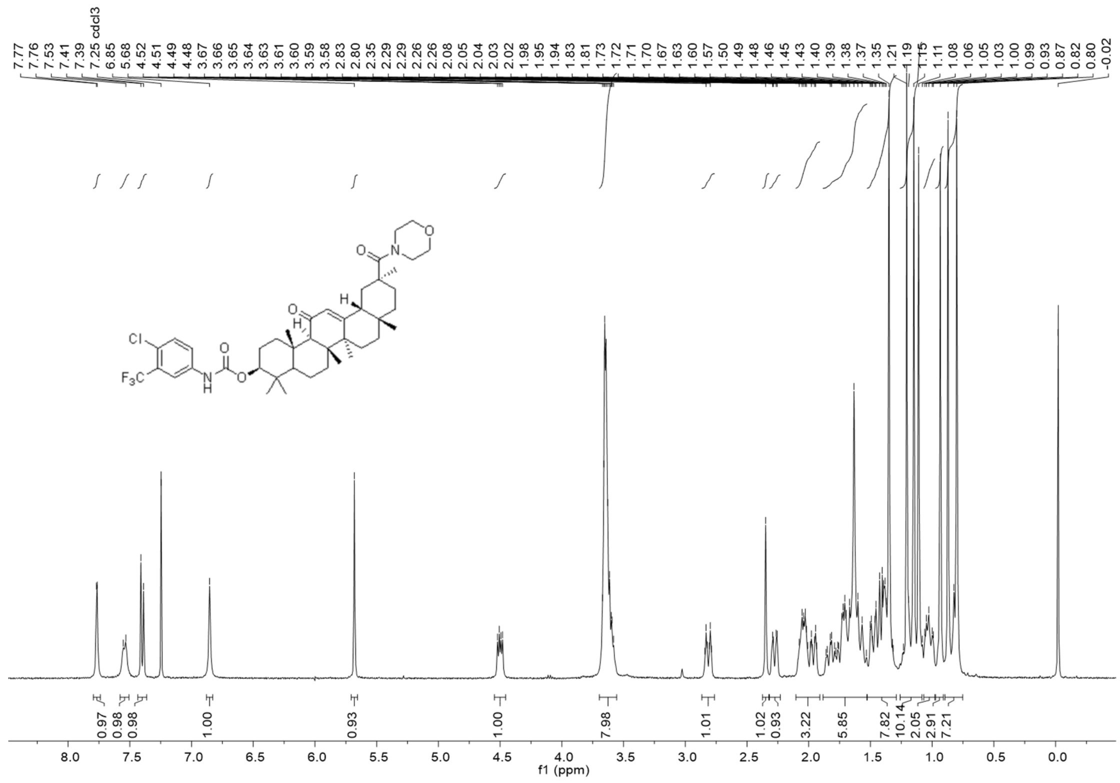

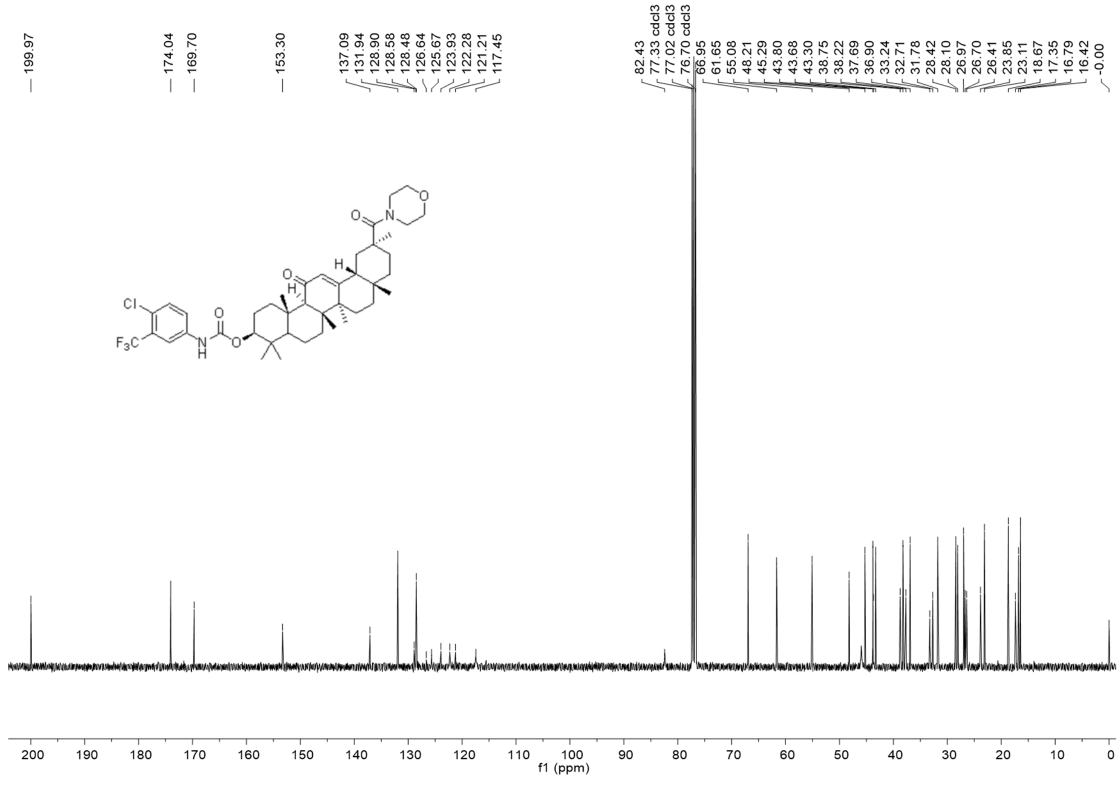

3*β*-(((3,5-dichlorophenyl)carbamoyl)oxy)-30-morpholino-olean-12-ene-11,30-dione **4c**

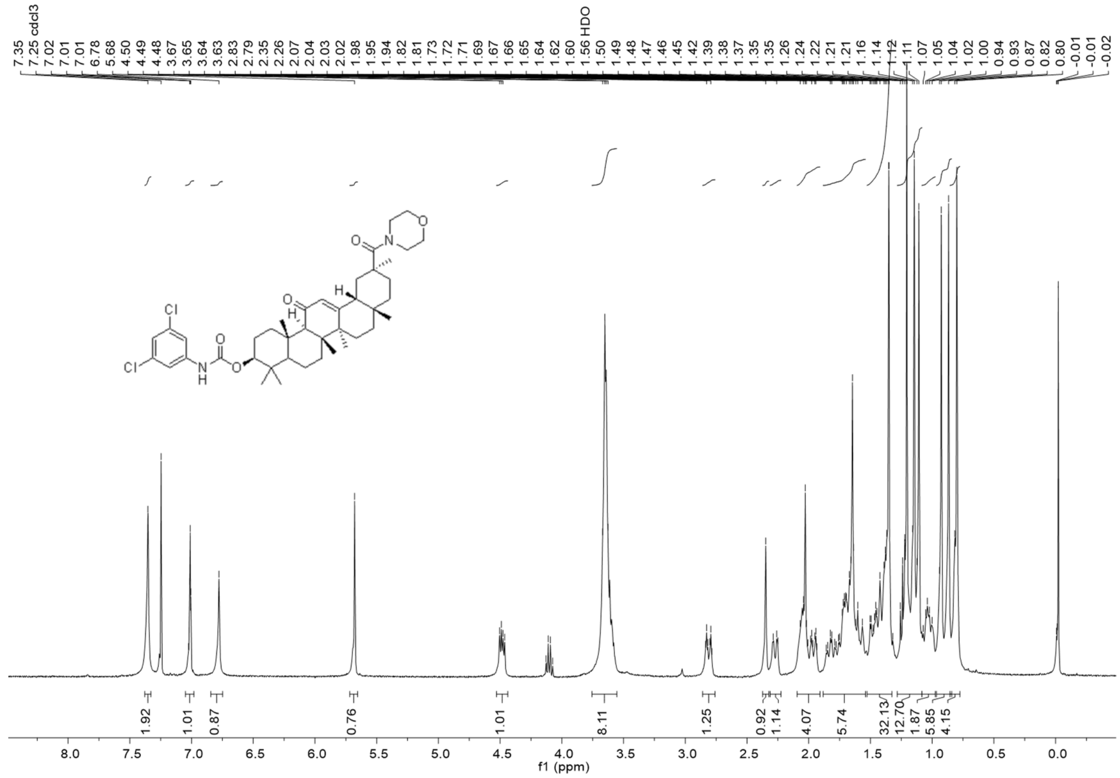

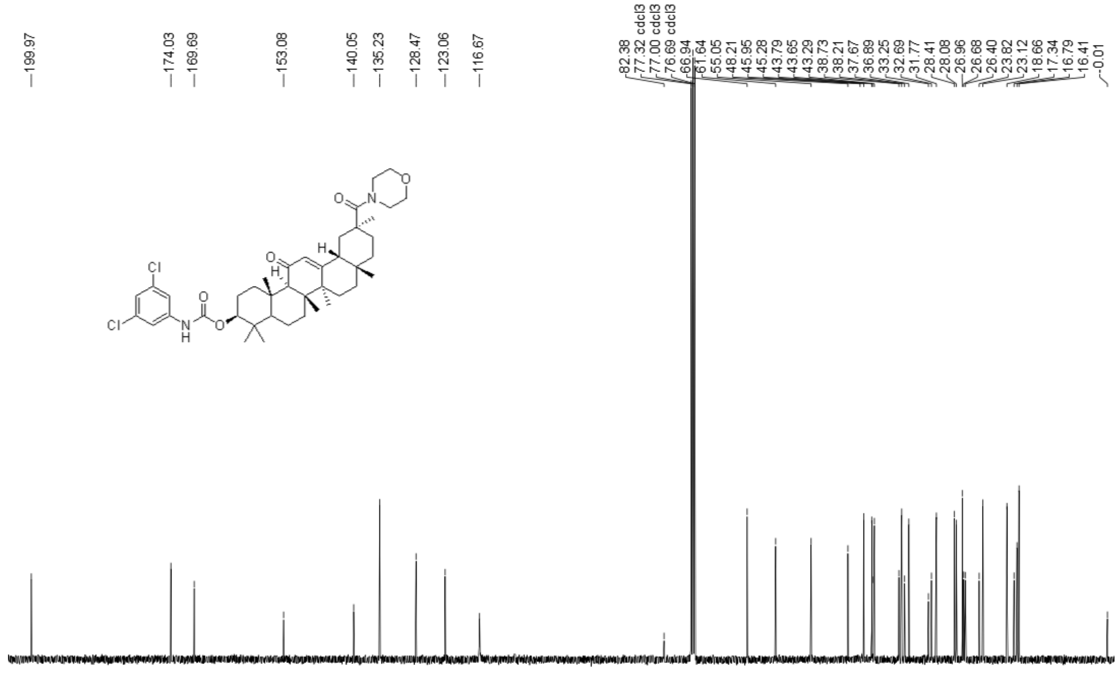

3*β*-(((4-chlorophenyl)carbamoyl)oxy)-30-morpholino-olean-12-ene-11,30-dione **4d**

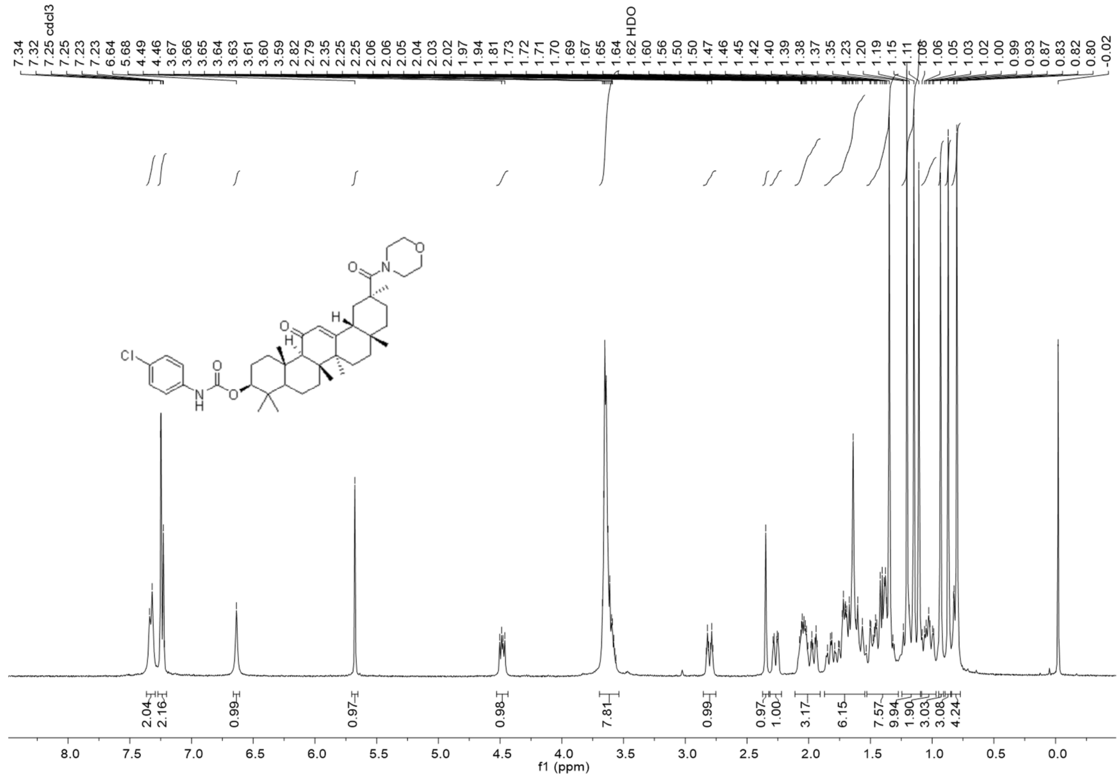

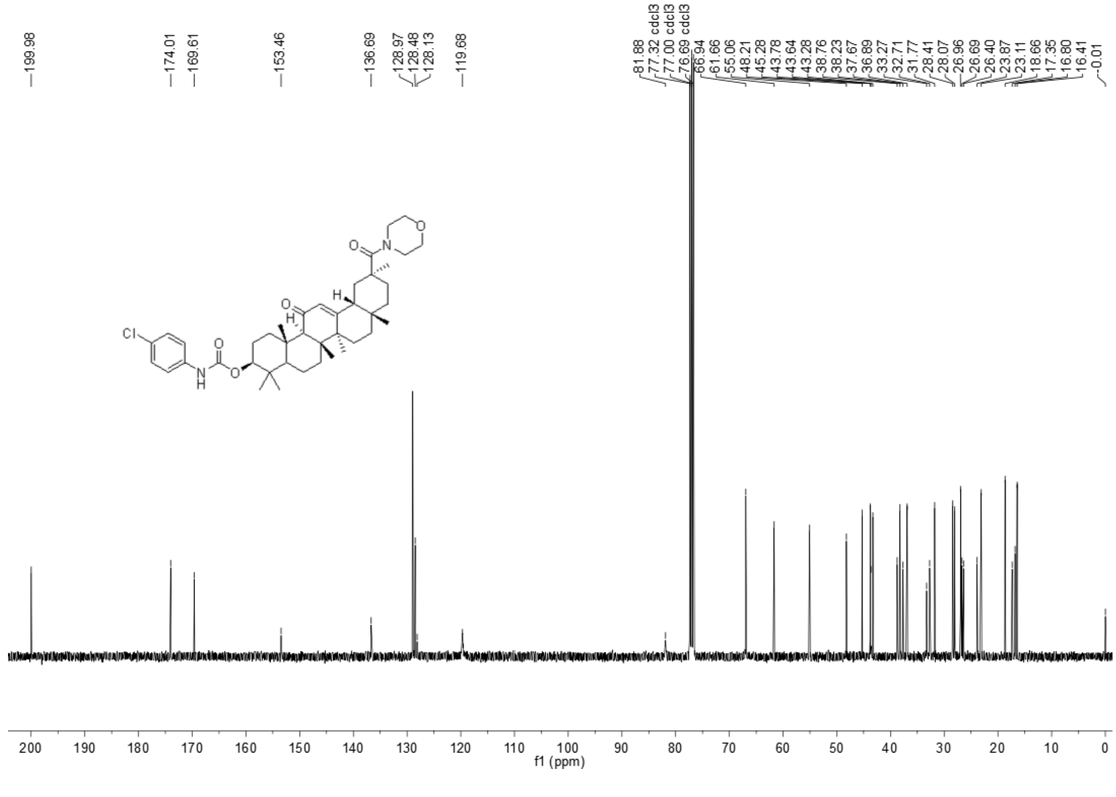

3*β*-(((3-chlorophenyl)carbamoyl)oxy)-30-morpholino-olean-12-ene-11,30-dione **4e**

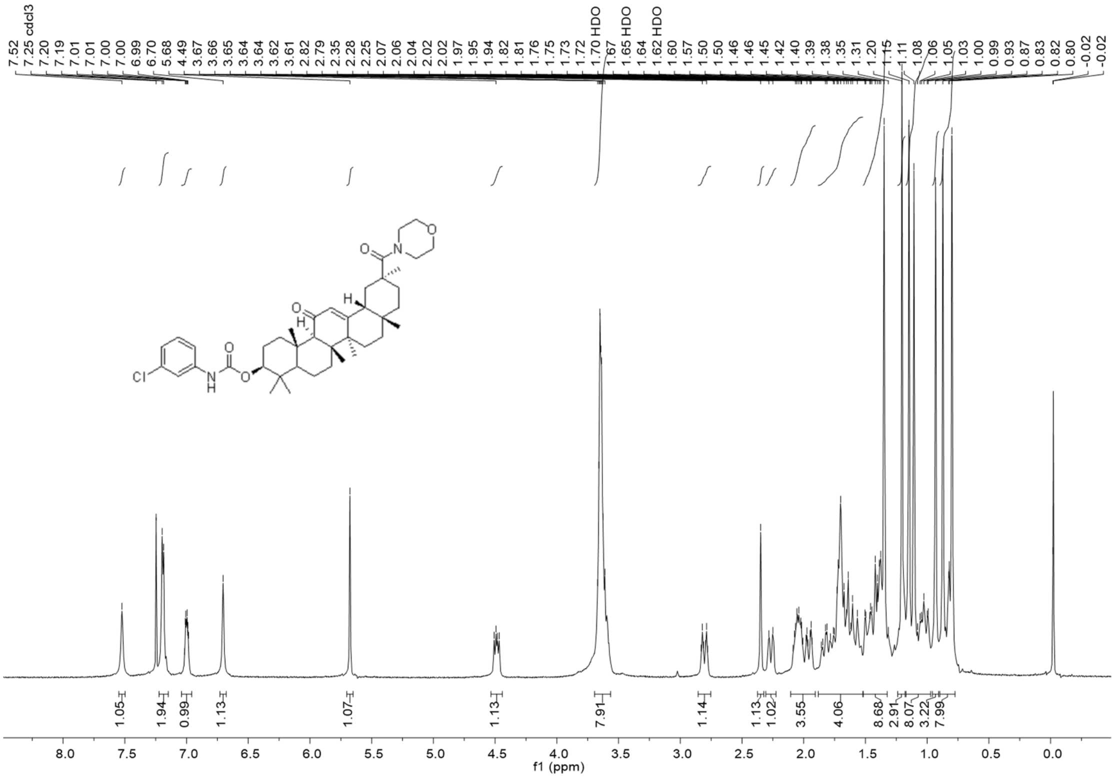

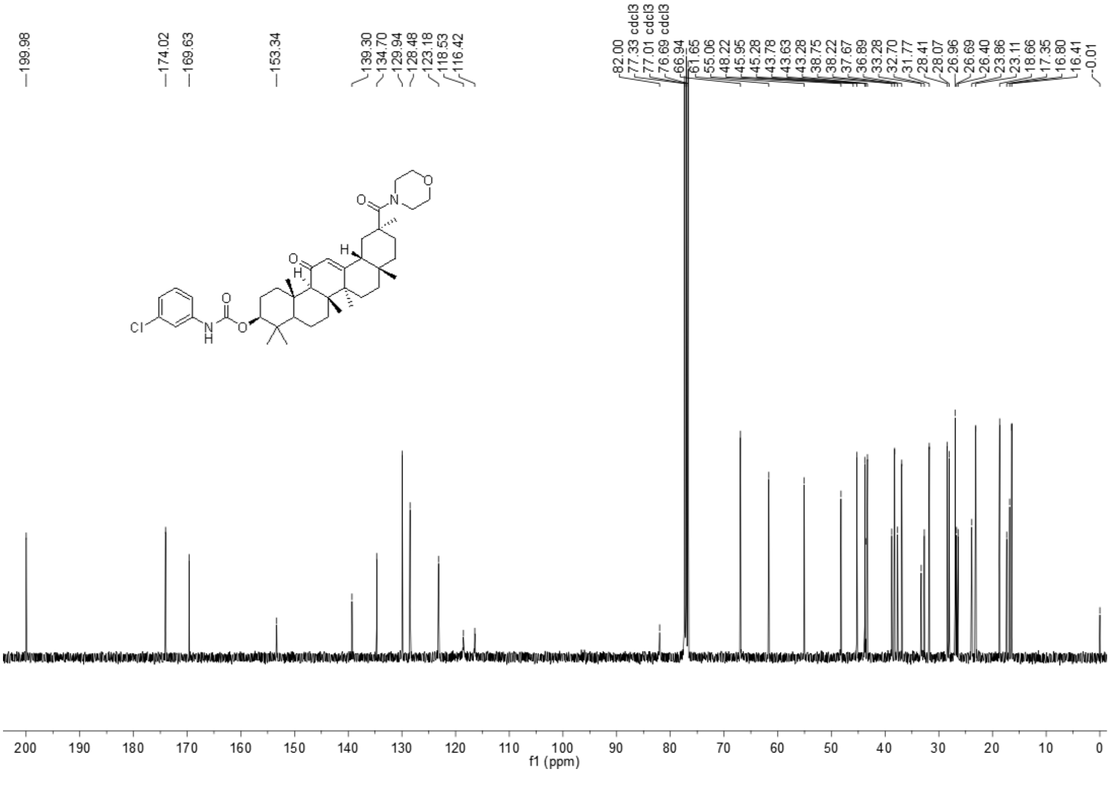

3*β*-(((3-chloro-4-methylphenyl)carbamoyl)oxy)-30-morpholino-olean-12-ene-11,30-dione **4f**

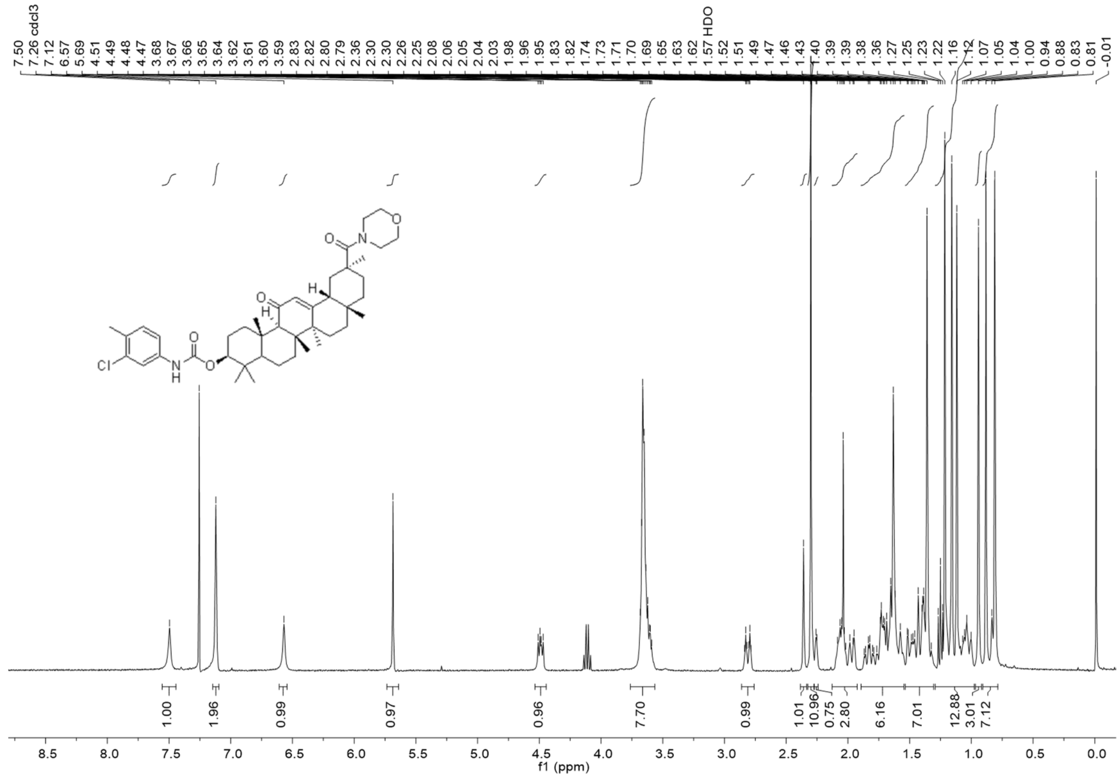

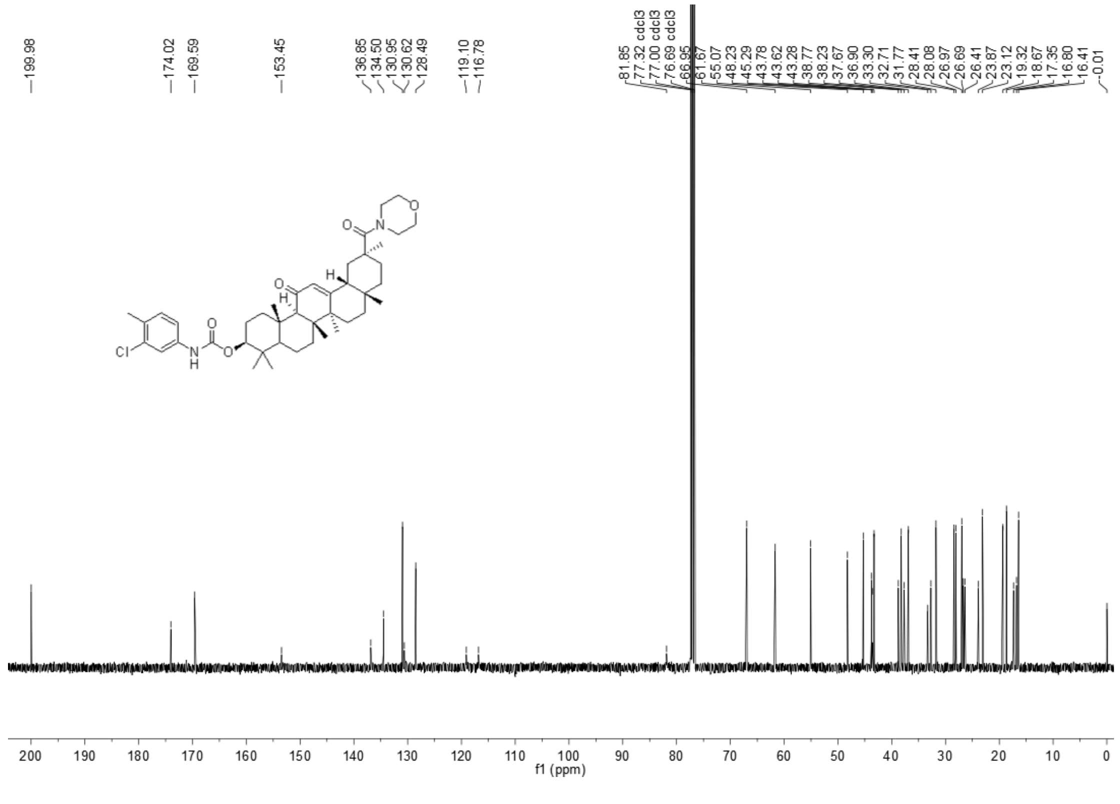

3*β*-(((4-bromophenyl)carbamoyl)oxy)-30-morpholino-olean-12-ene-11,30-dione **4g**

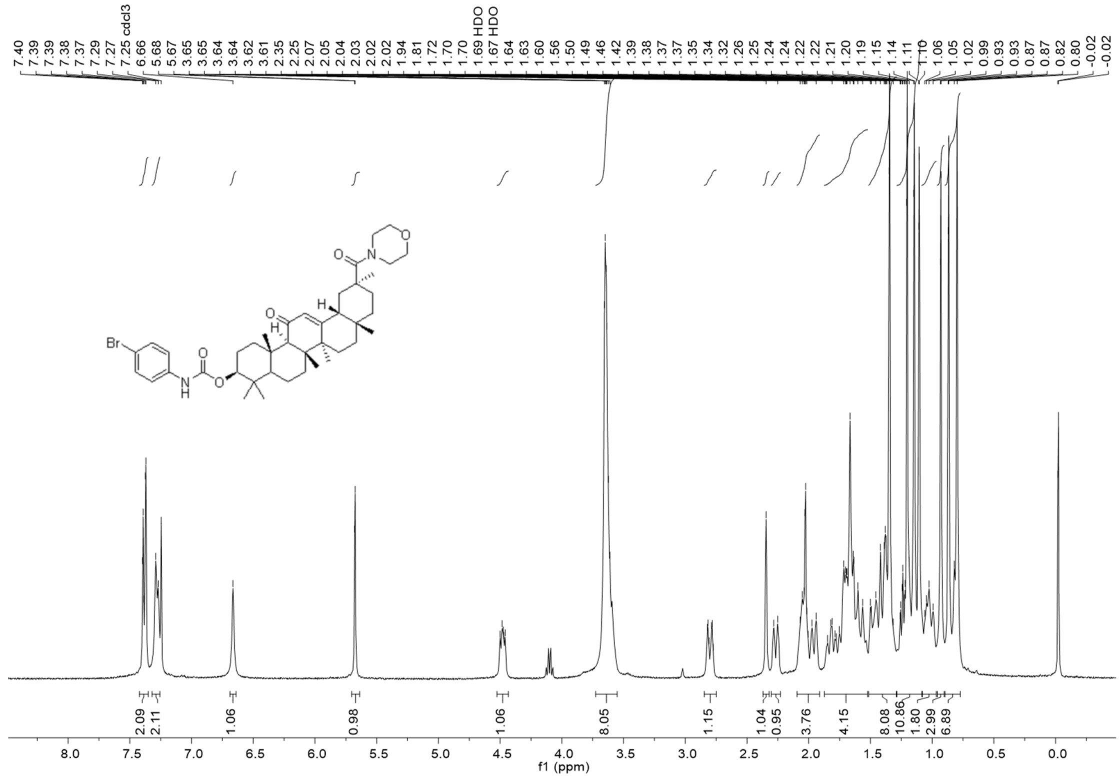

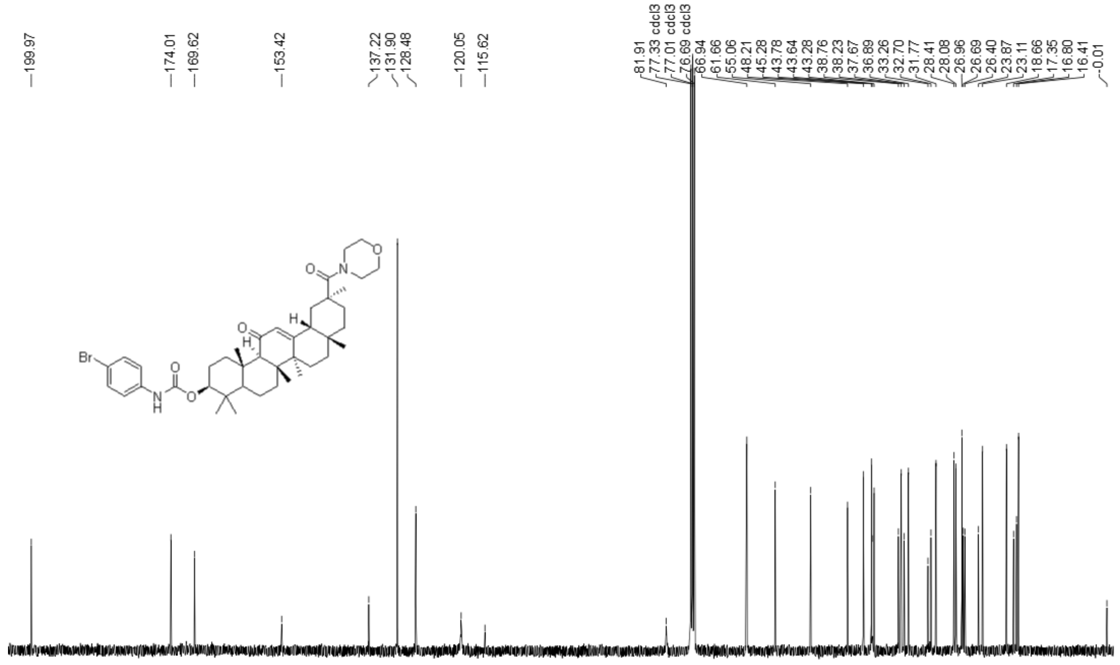

3*β*-(((4-fluorophenyl)carbamoyl)oxy)-30-morpholino-olean-12-ene-11,30-dione **4h**

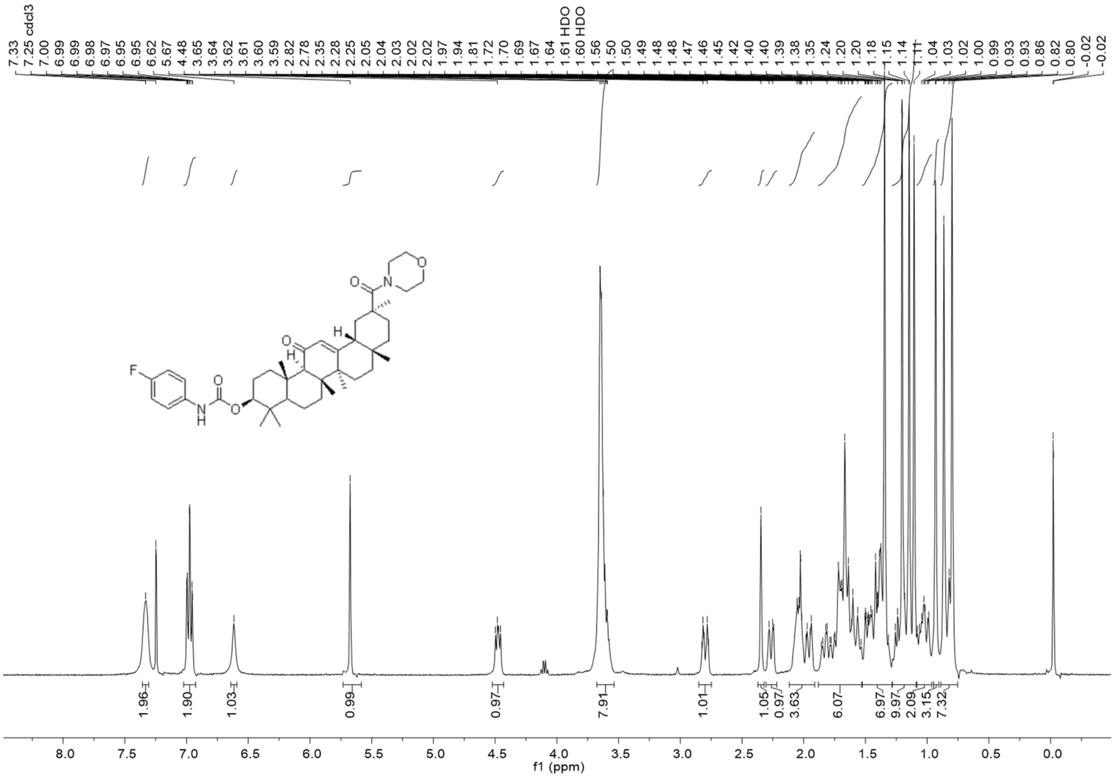

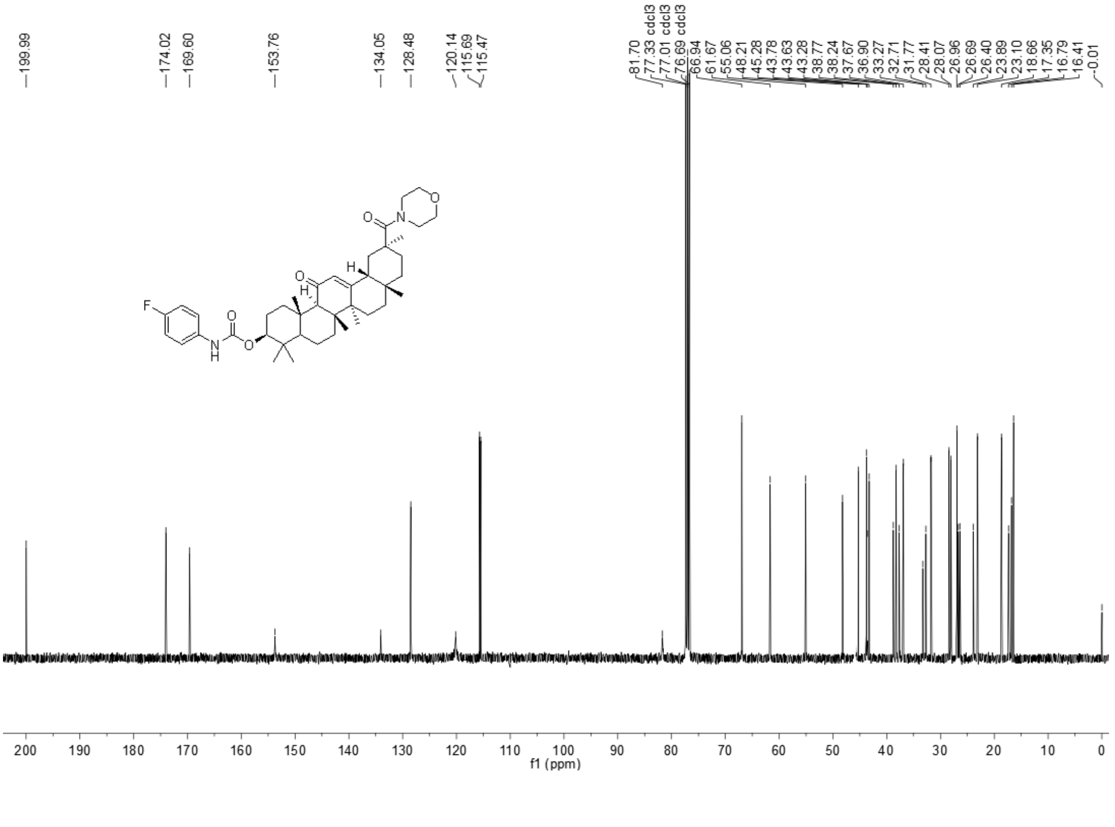

3*β*-(((4-(trifluoromethyl)phenyl)carbamoyl)oxy)-30-morpholino-olean-12-ene-11,30-dione **4i**

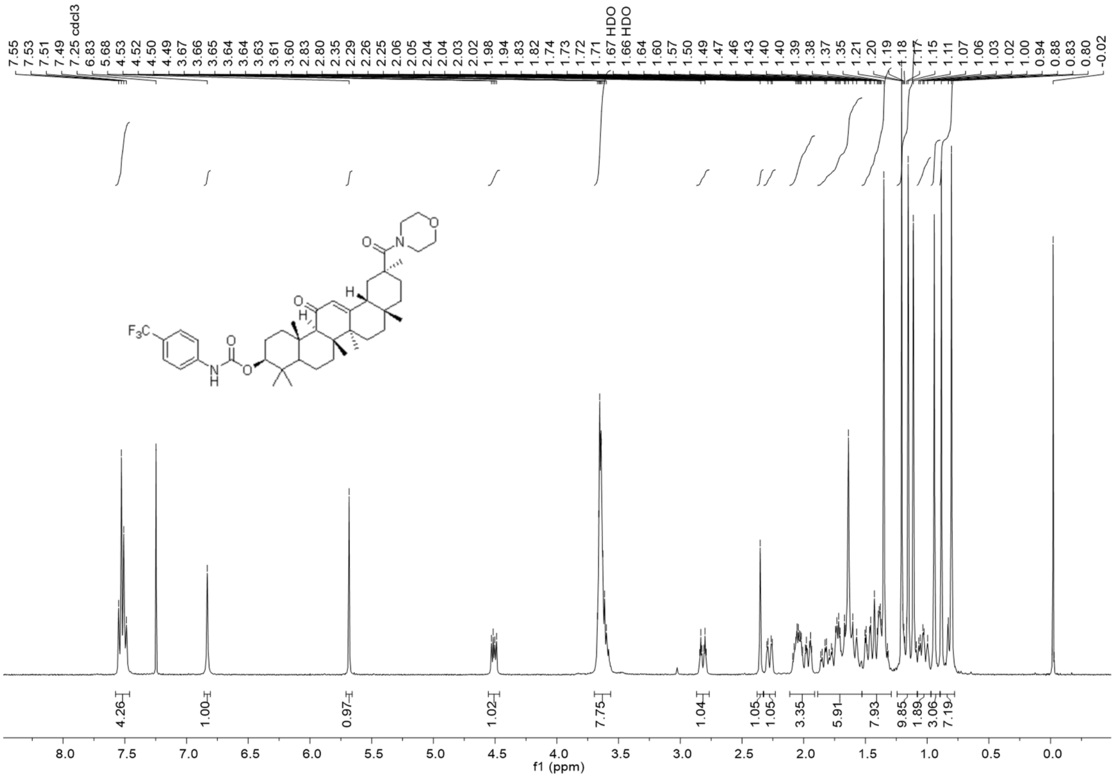

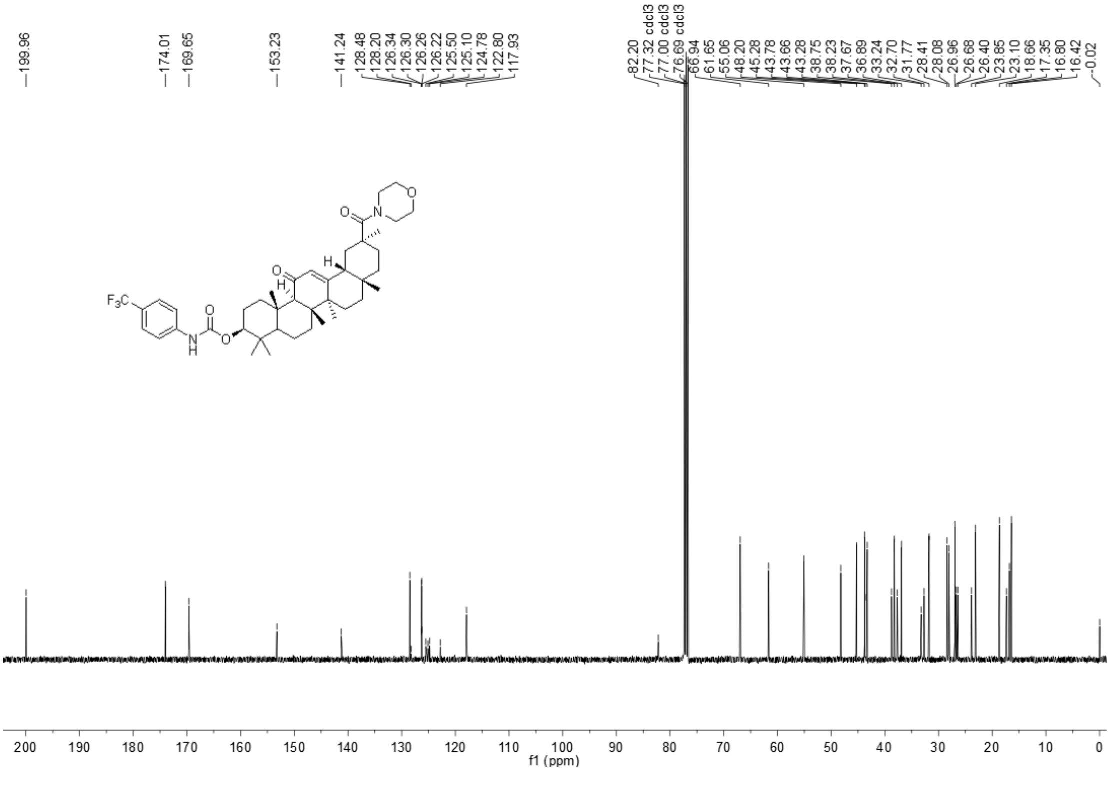

3*β*-(((3-(trifluoromethyl)phenyl)carbamoyl)oxy)-30-morpholino-olean-12-ene-11,30-dione **4j**

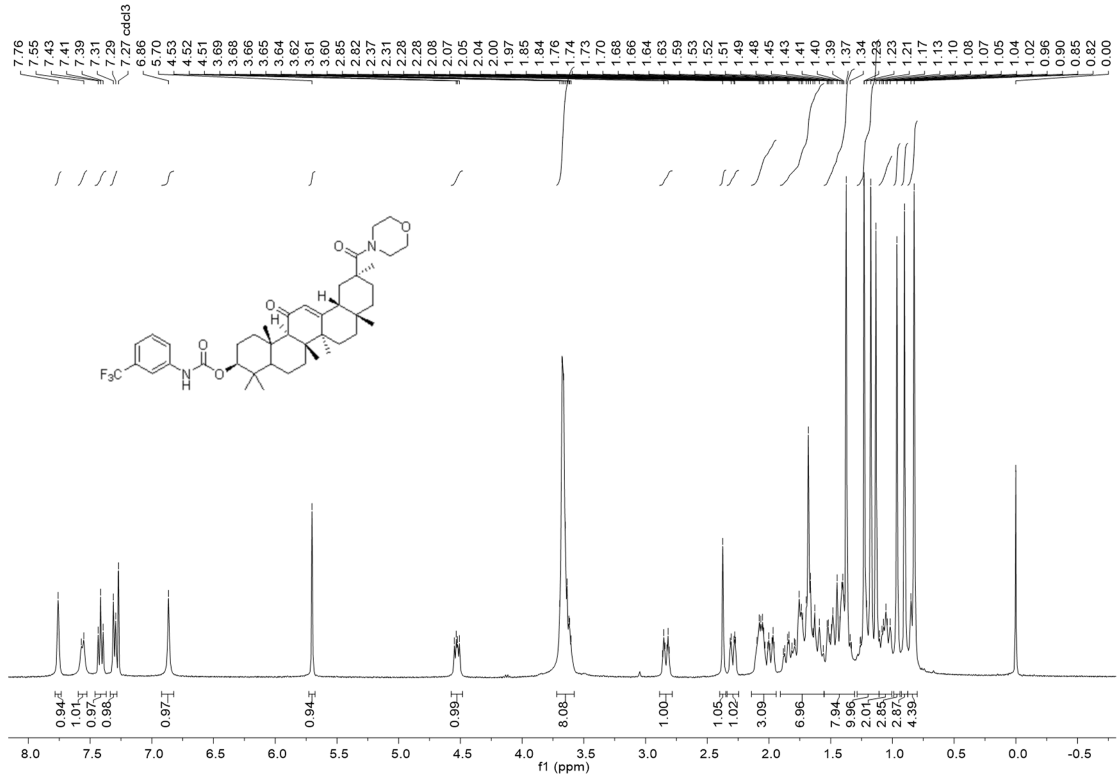

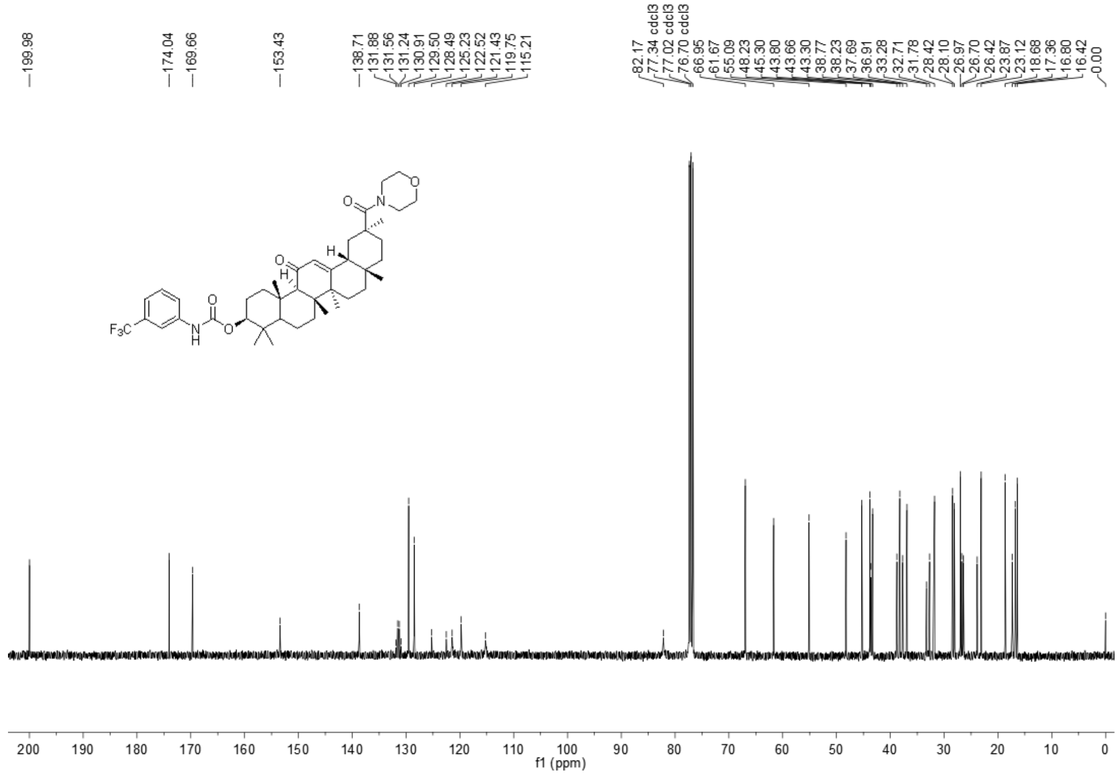

3*β*-(((3,5-bis(trifluoromethyl)phenyl)carbamoyl)oxy)-30-morpholino-olean-12-ene-11,30-dione **4k**

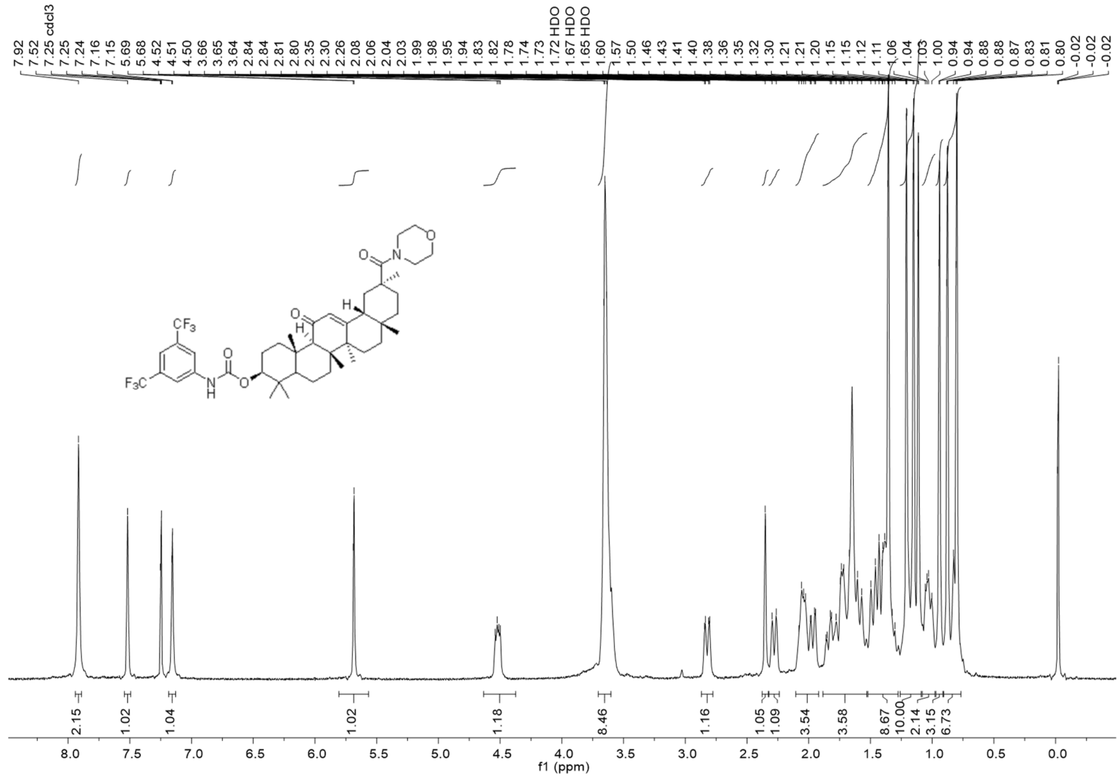

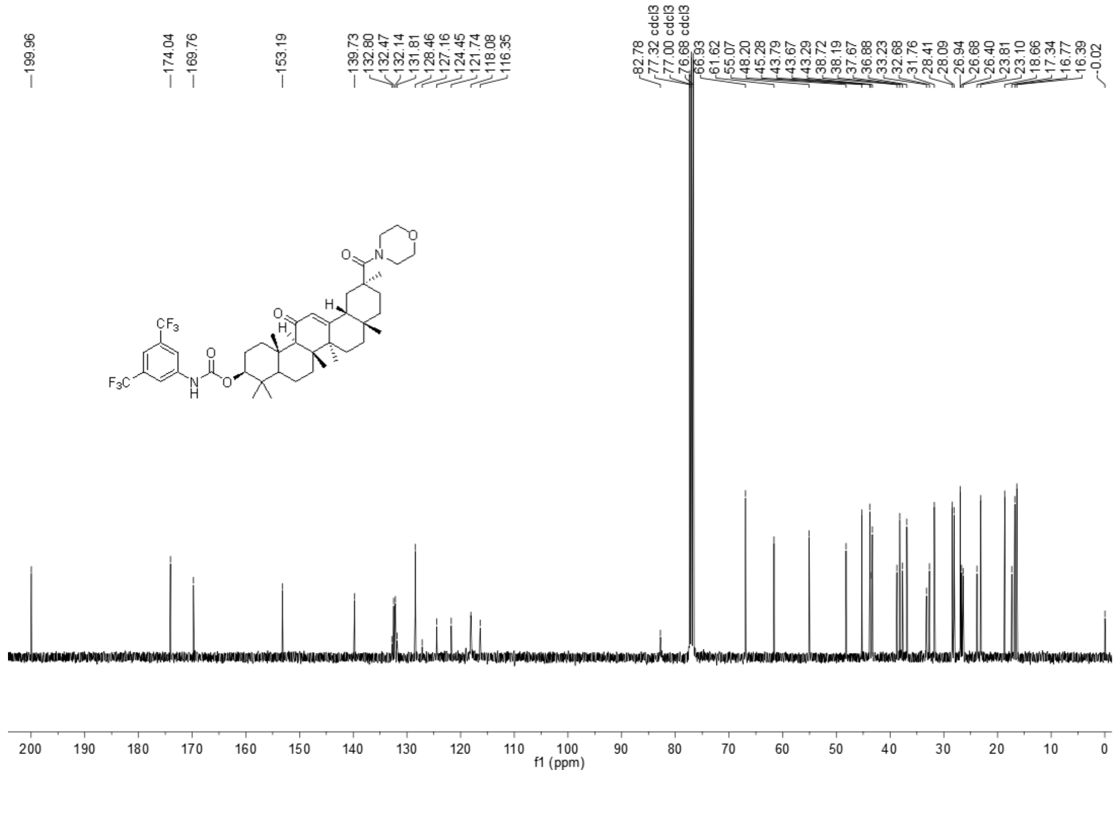

3*β*-(((3-methoxyphenyl)carbamoyl)oxy)-30-morpholino-olean-12-ene-11,30-dione **4l**

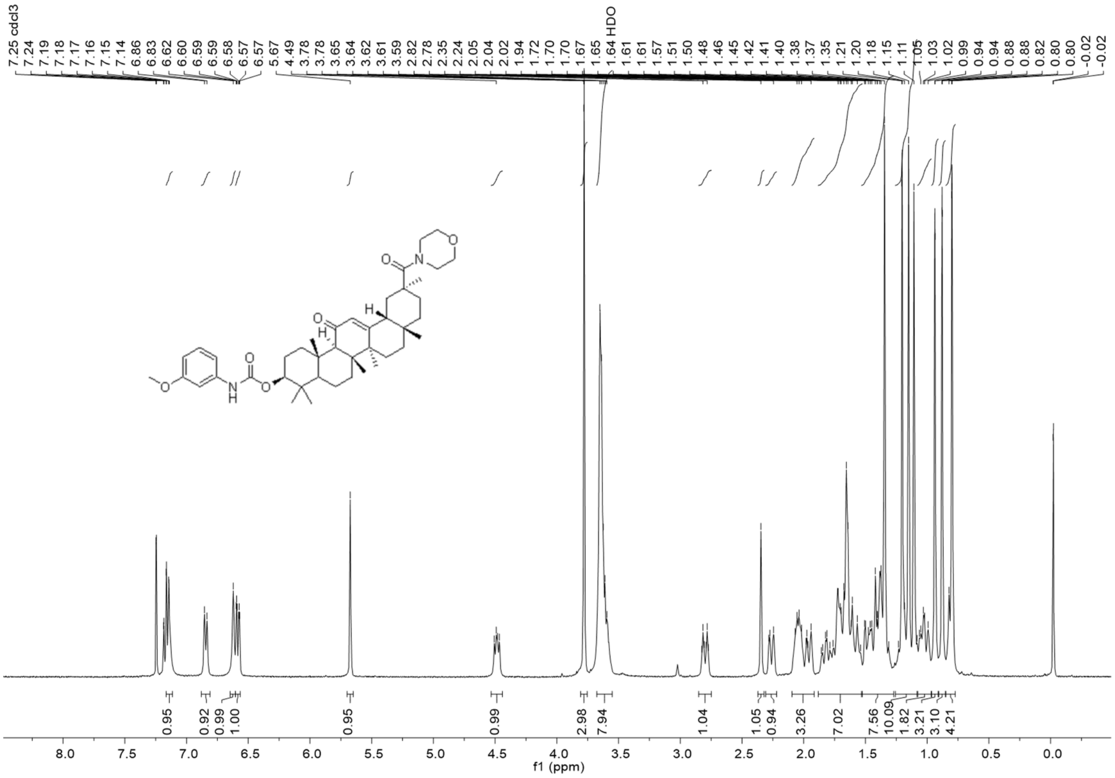

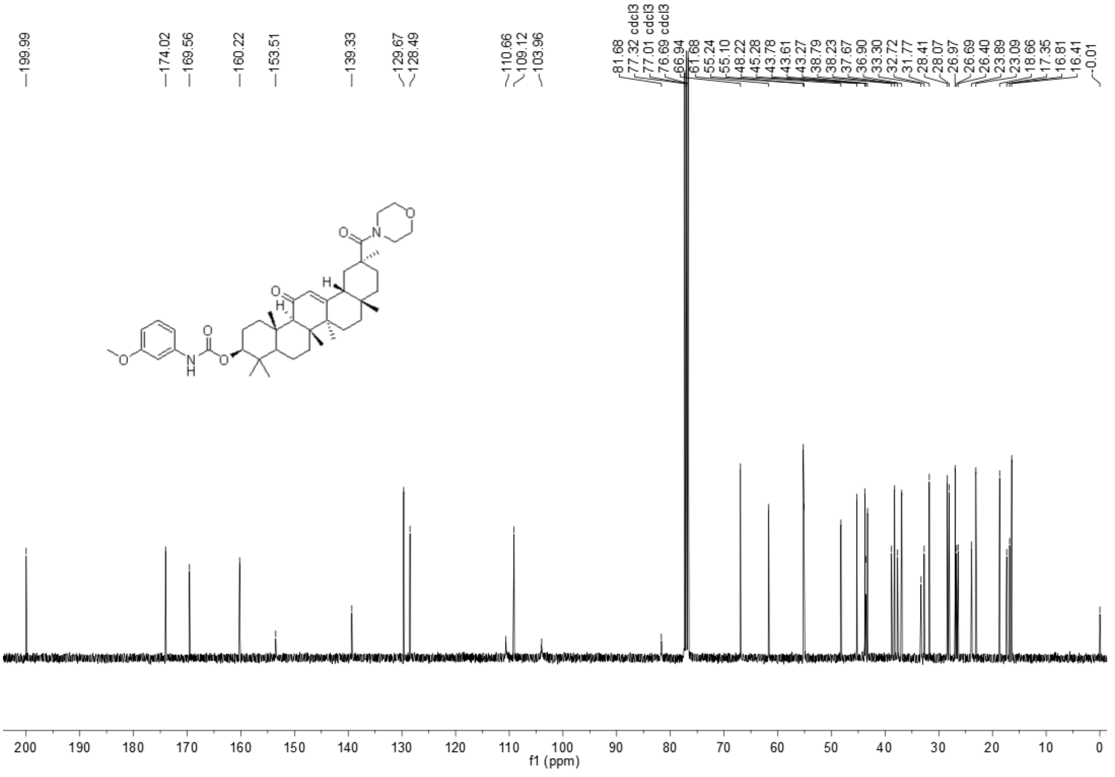

3*β*-(((4-methoxyphenyl)carbamoyl)oxy)-11-oxo-olean-12-en-30-oyl morpholine **4m**

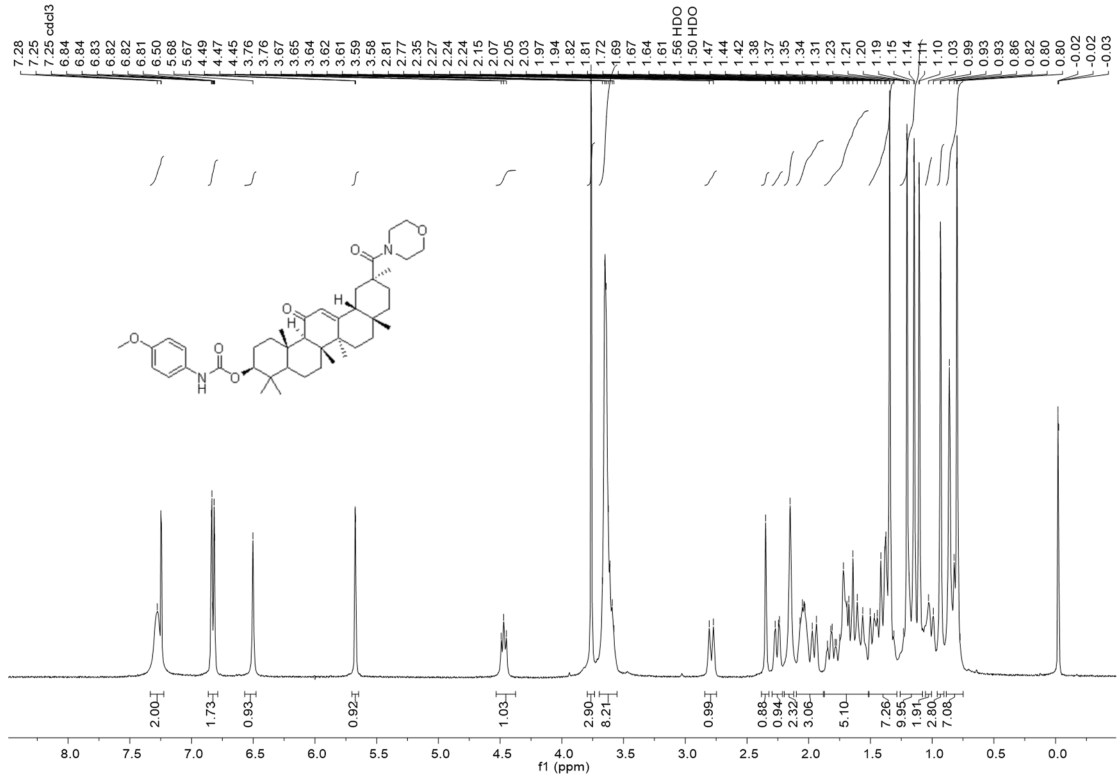

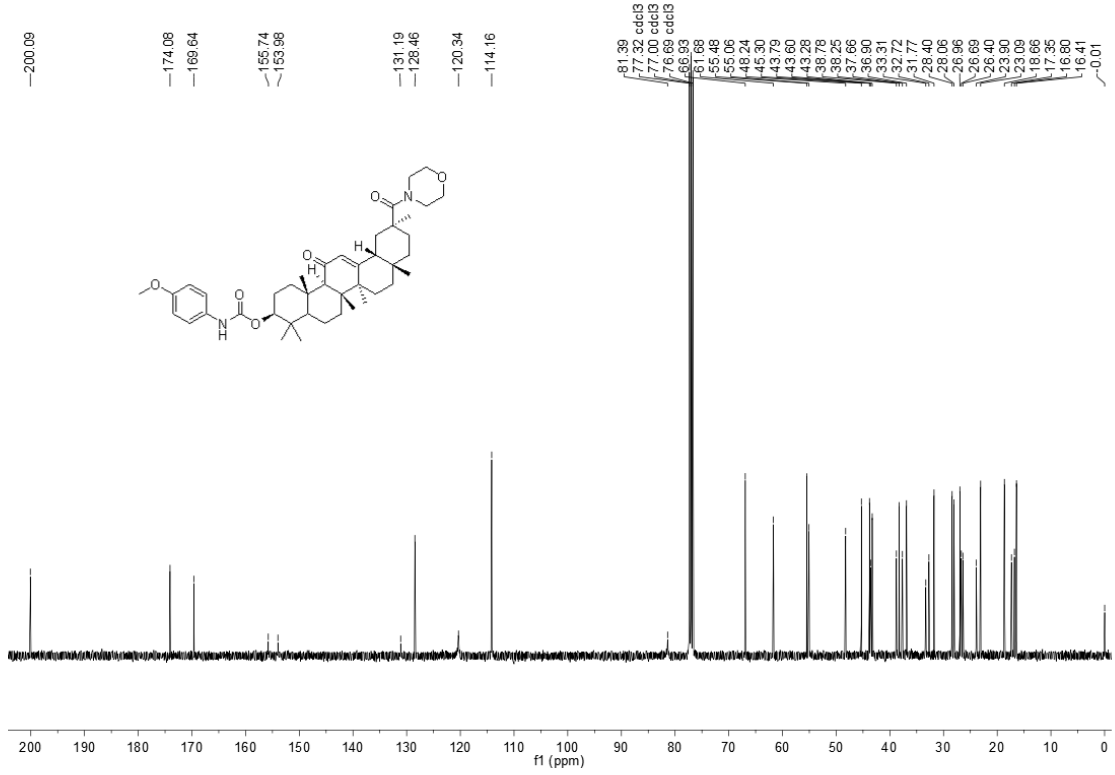

3*β*-(((4-(trifluoromethoxy)phenyl)carbamoyl)oxy)-30-morpholino-olean-12-ene-11,30-dione

4n

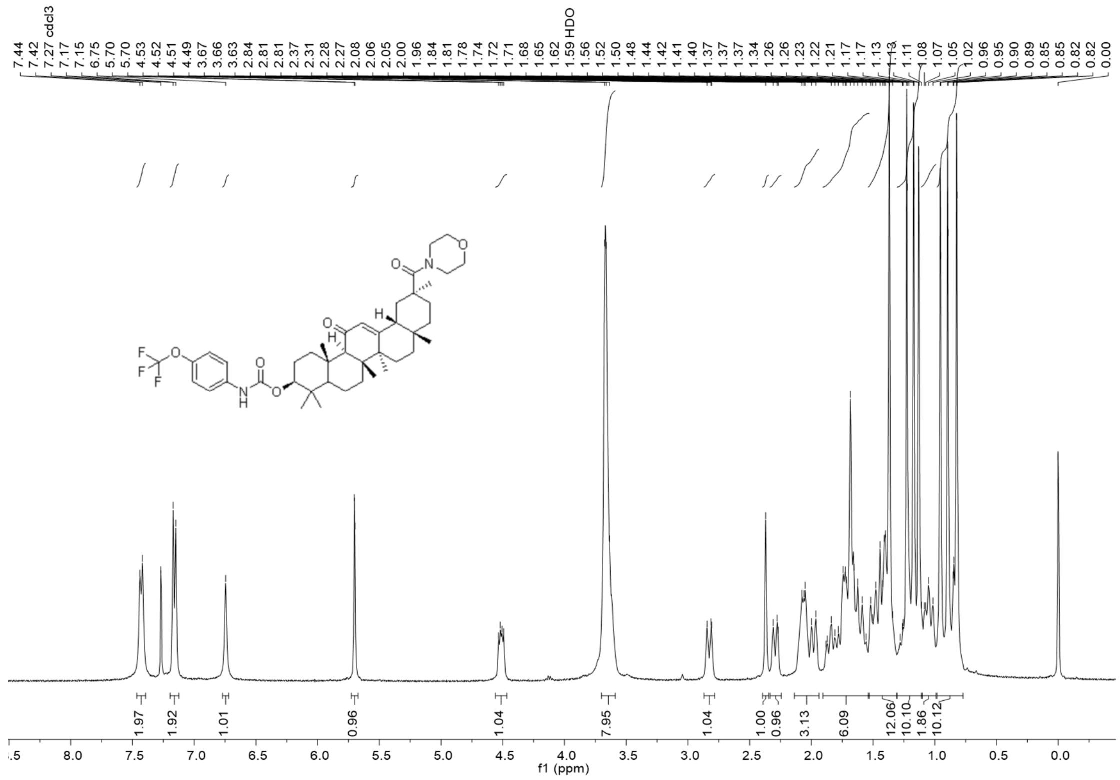

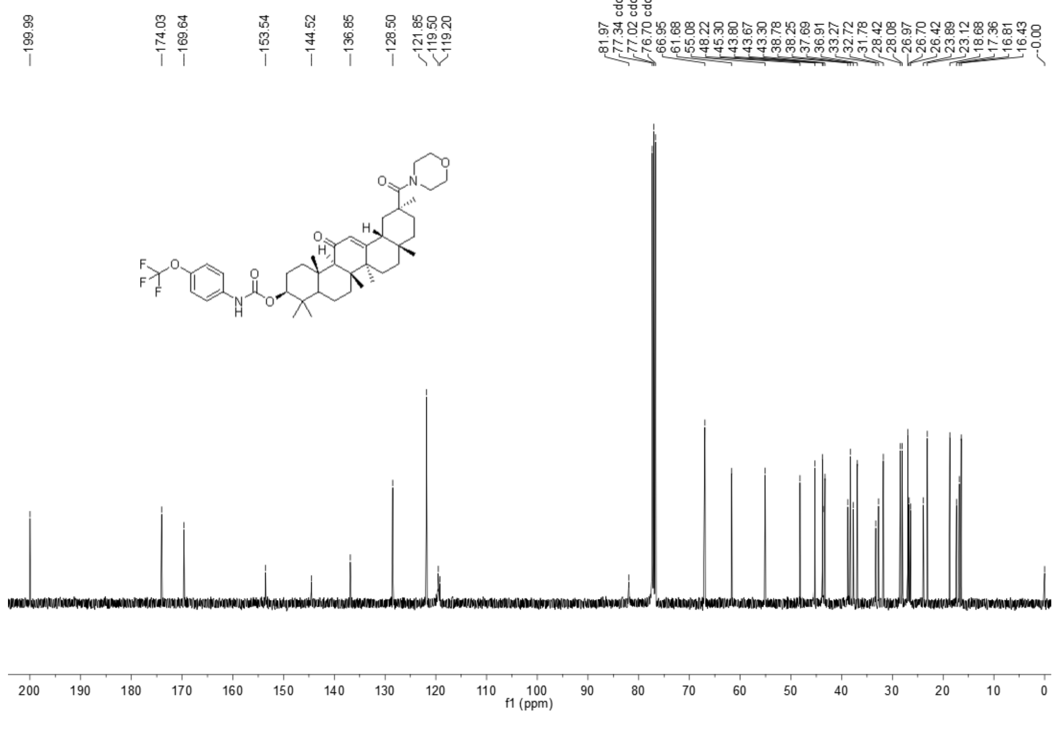

3*β*-(2-chloroacetyloxy)-11-oxo-olean-12-en-30-oic acid **5**

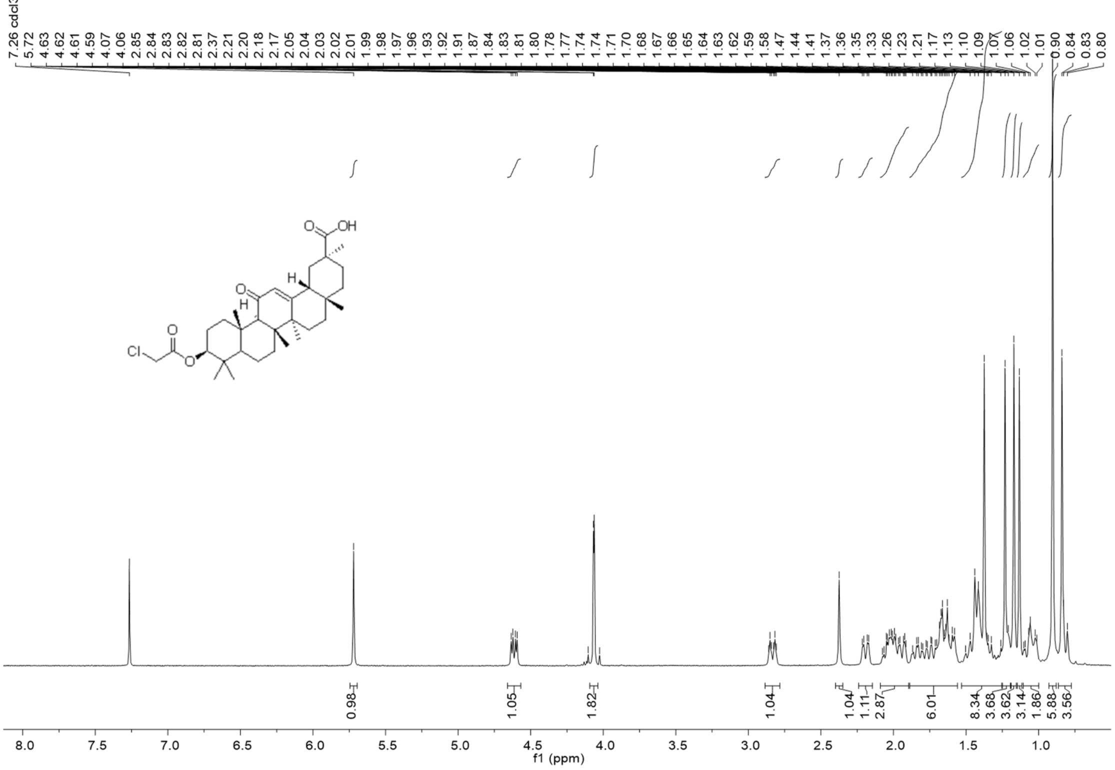

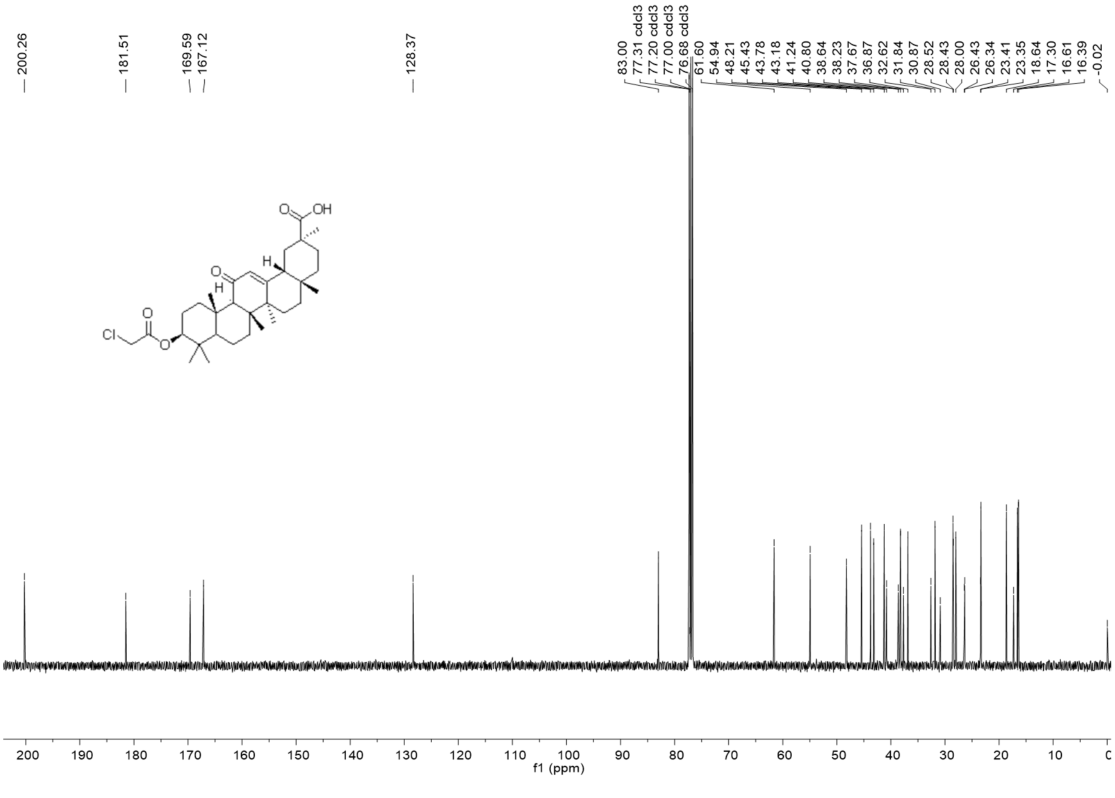

3*β*-(2-morpholinoacetoxy)-11-oxo-olean-12-en-30-oic acid **6a**

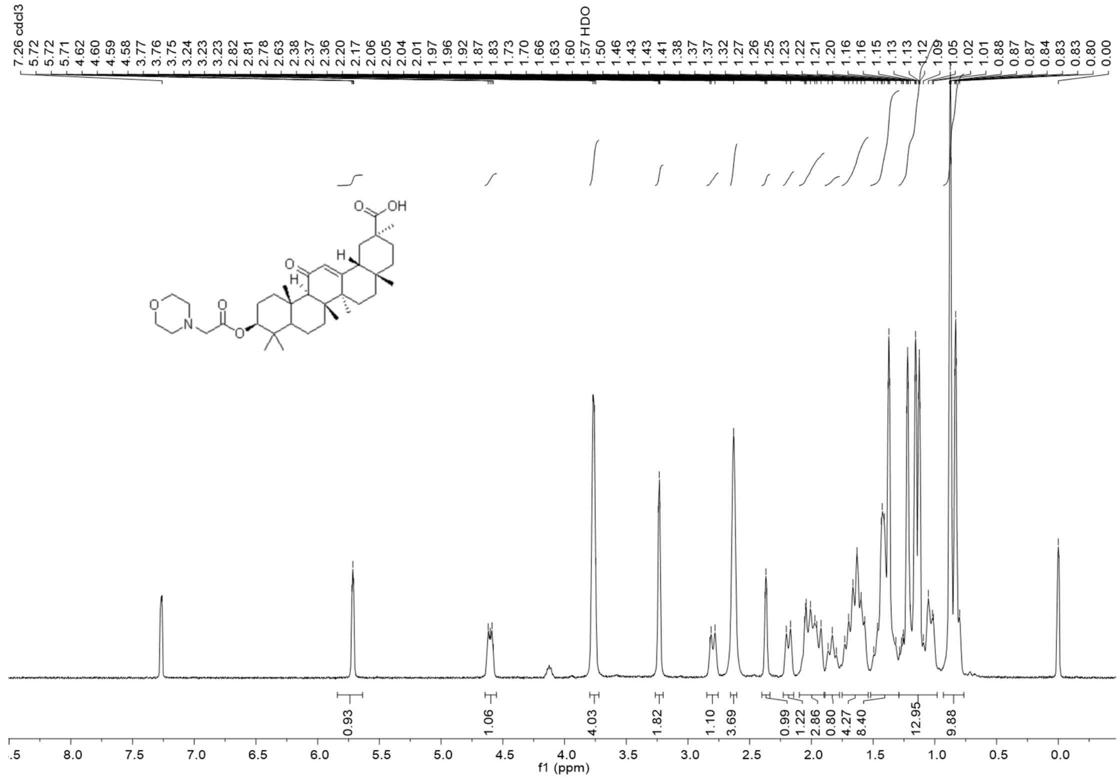

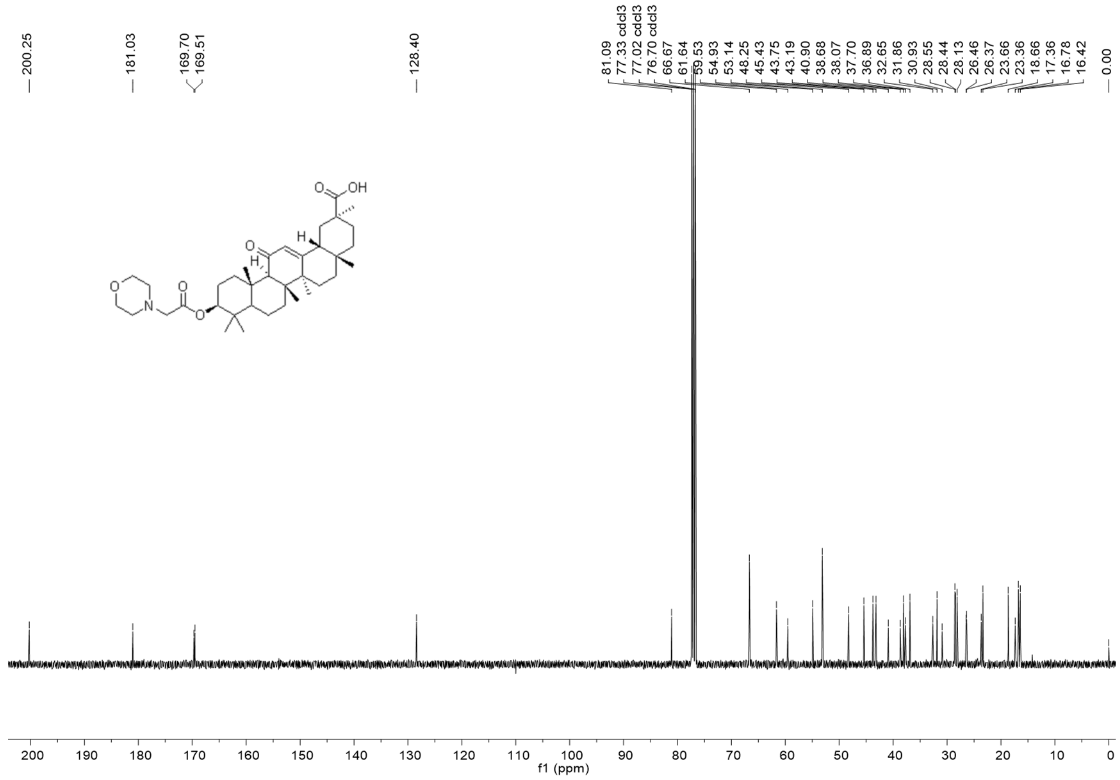

3*β*-(2-(4-carbamoylpiperidin-1-yl)acetoxy)-11-oxo-olean-12-en-30-oic acid **6b**

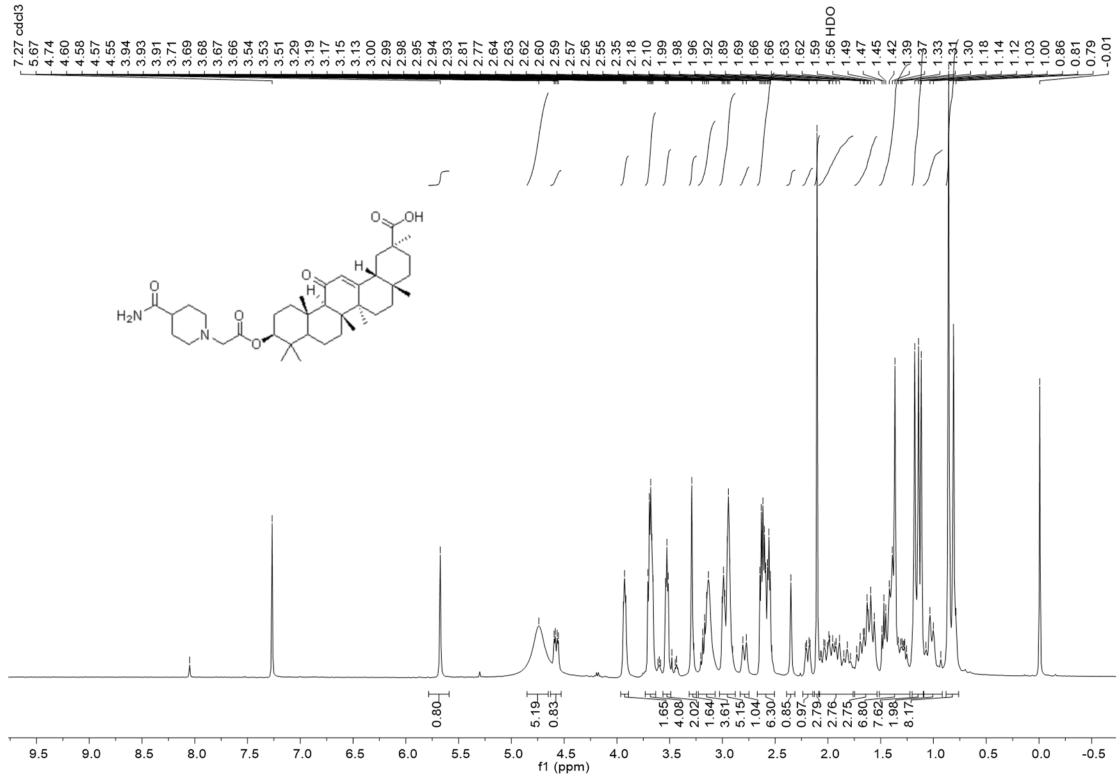

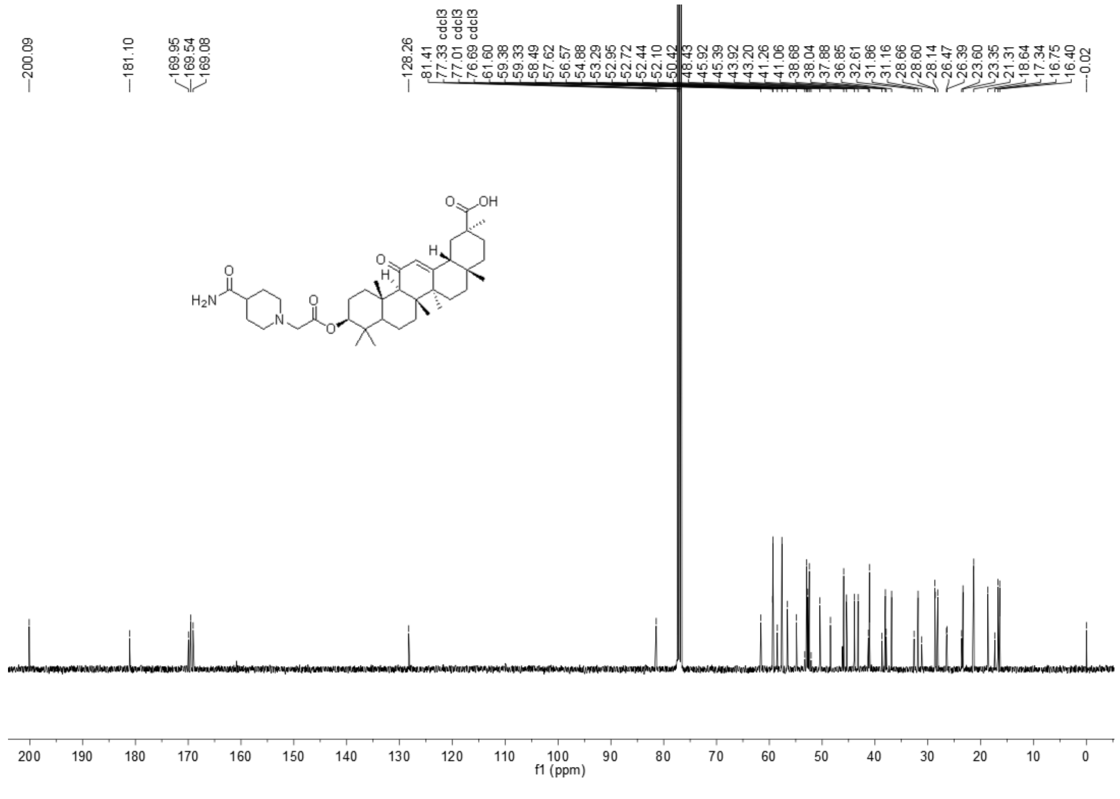

3*β*-(2-(4-methylpiperazin-1-yl)acetoxy)-11-oxo-olean-12-en-30-oic acid **6c**

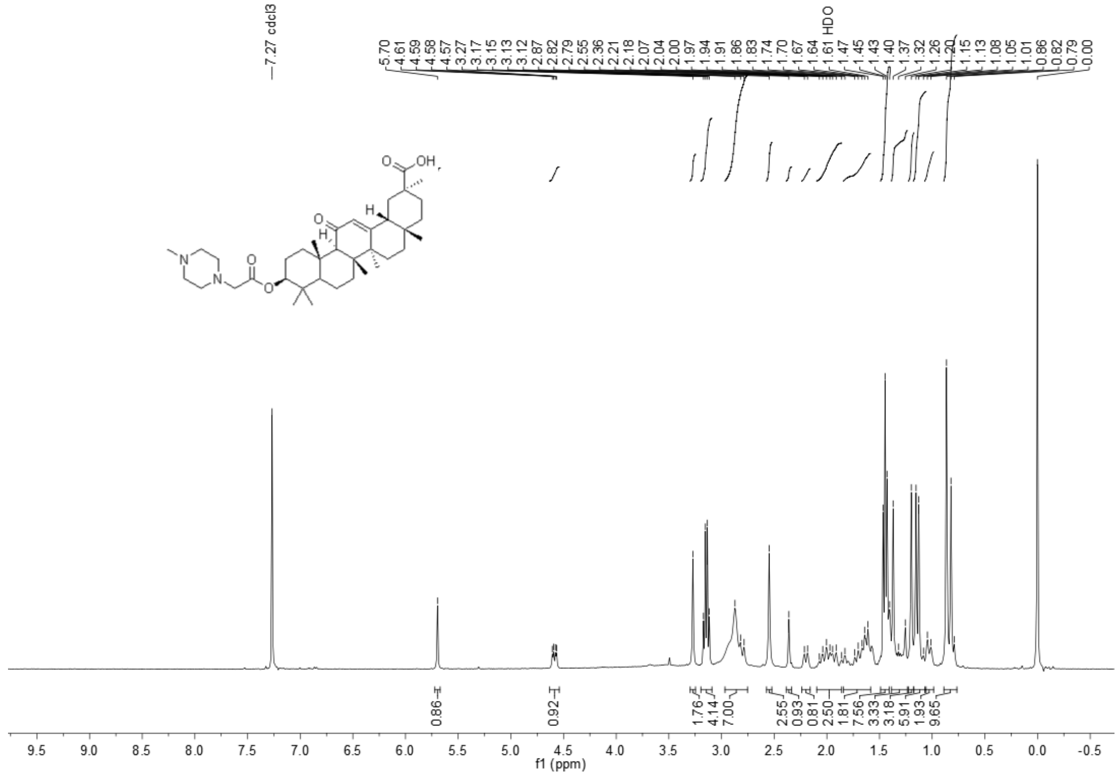

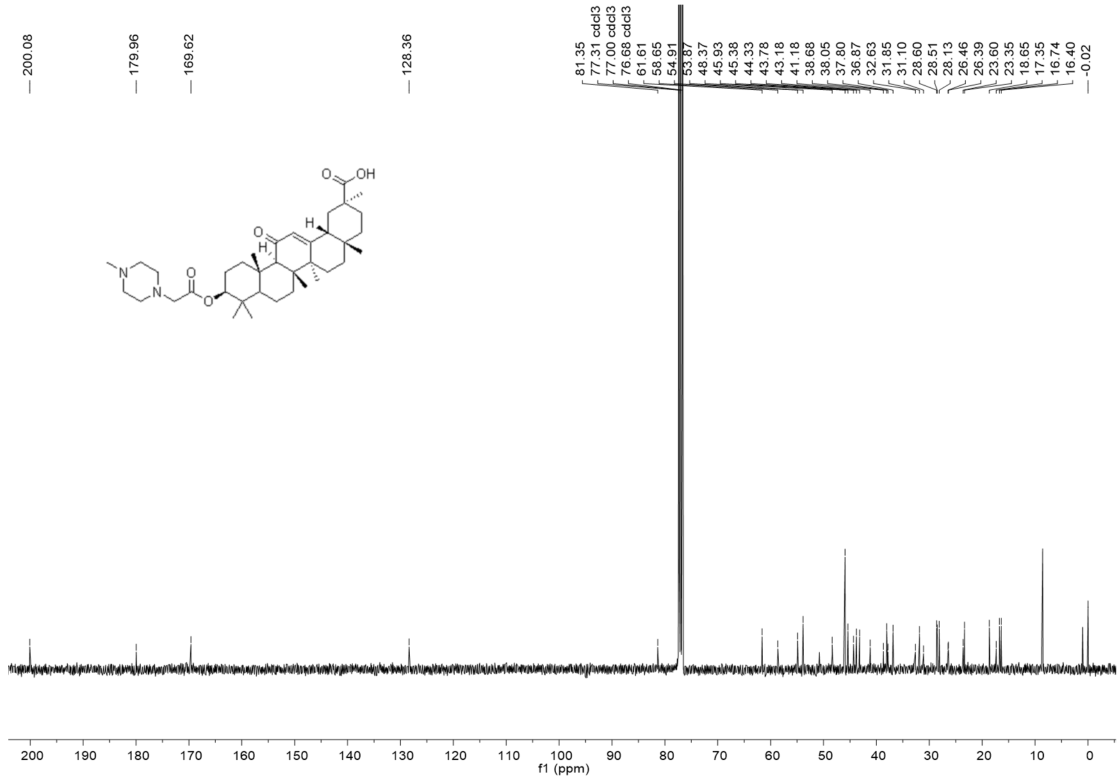

3*β*-(2-(4-(pyridin-2-yl)piperazin-1-yl)acetoxy)-11-oxo-olean-12-en-30-oic acid **6d**

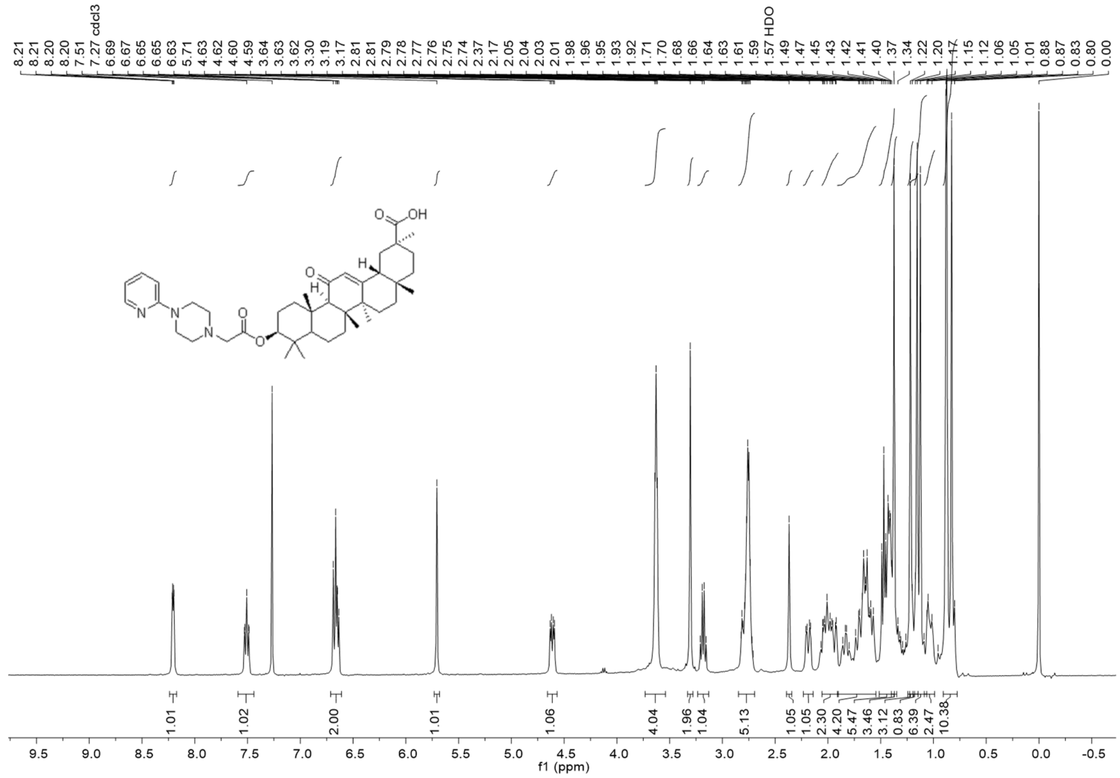

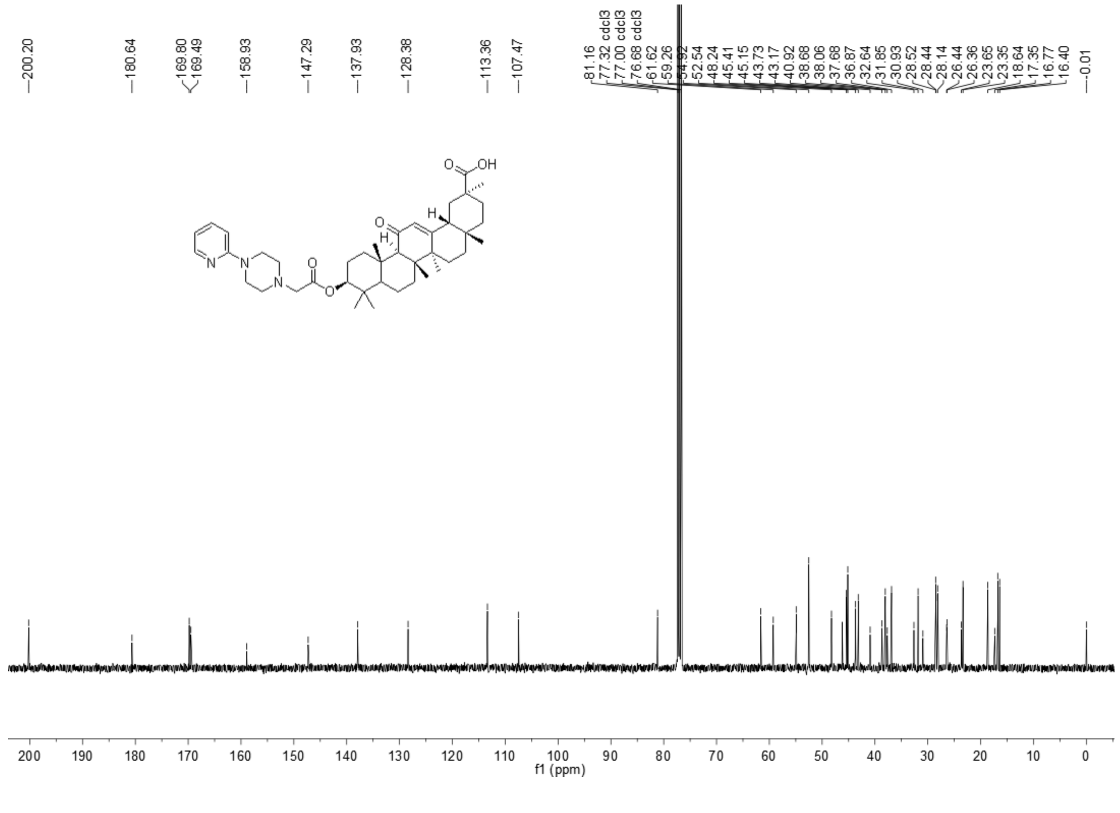

3*β*-(2-(4-chlorophenyl)acetoxy)-11-oxo-olean-12-en-30-oic acid **7a**

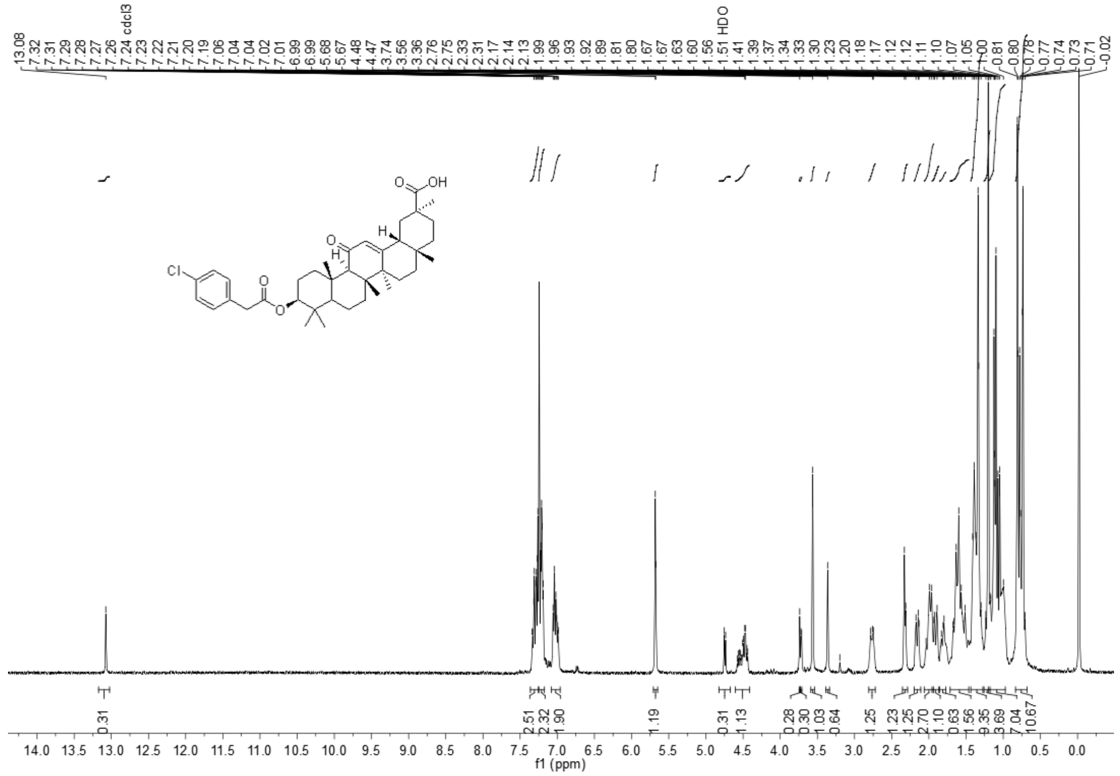

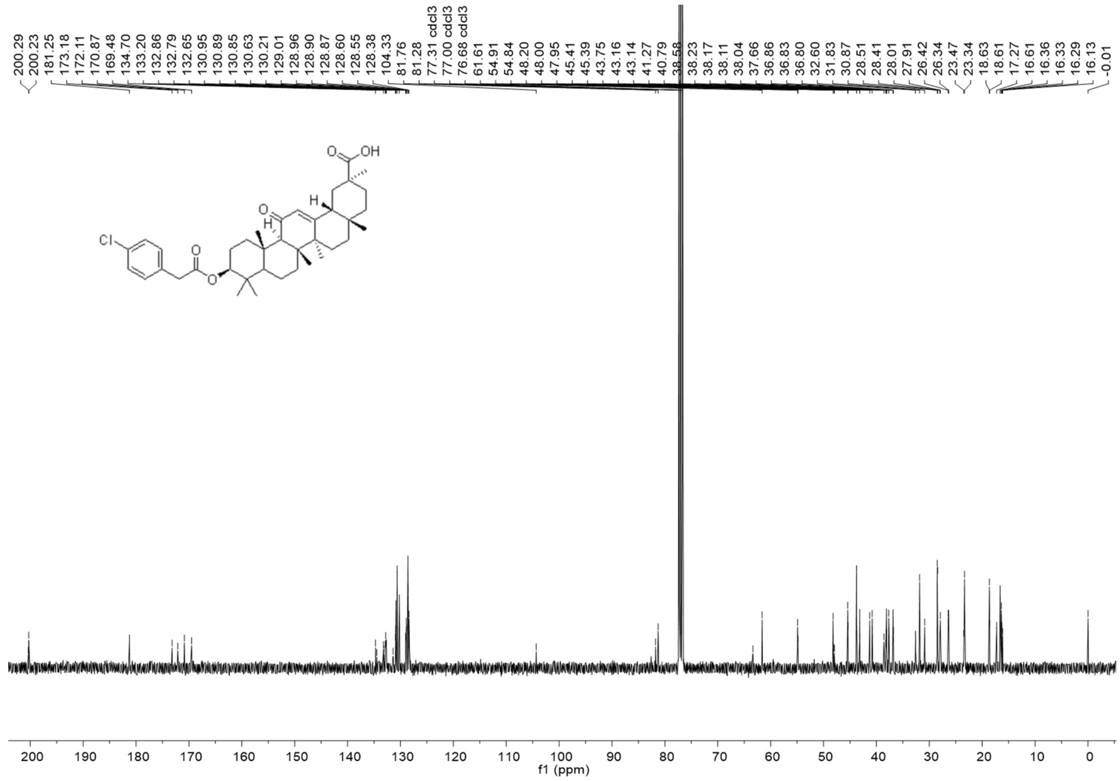

3*β*-(2-(4-fluorophenyl)acetoxy)-11-oxo-olean-12-en-30-oic acid **7b**

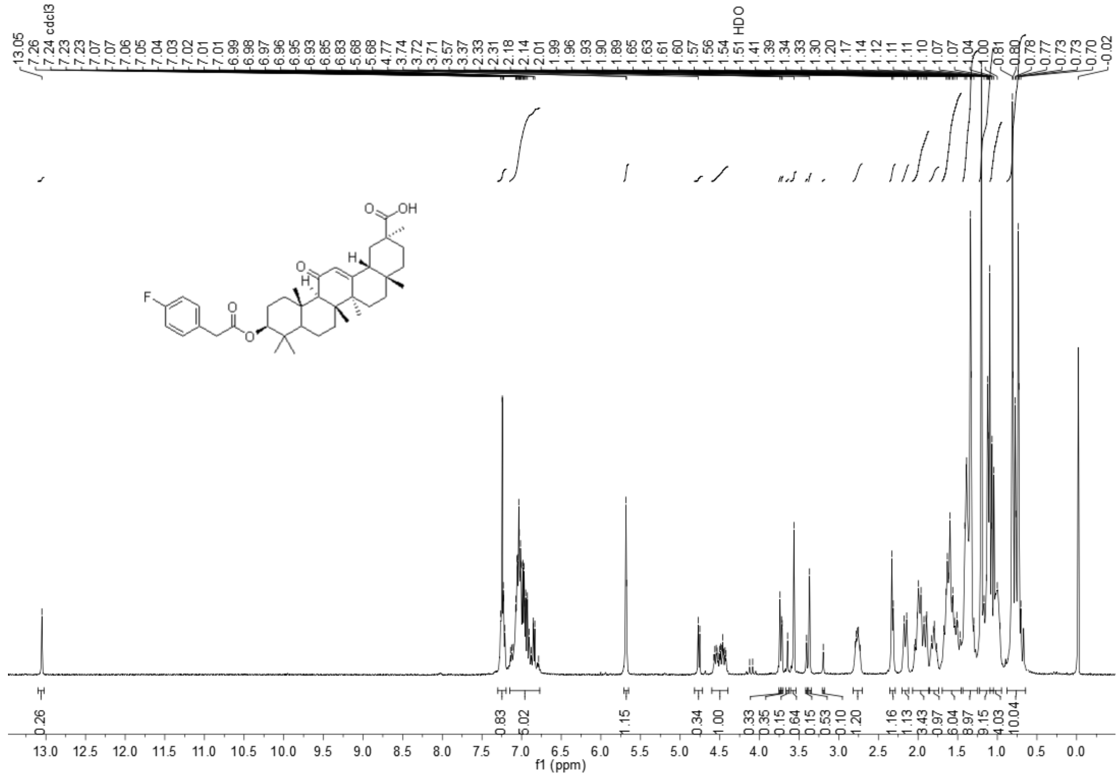

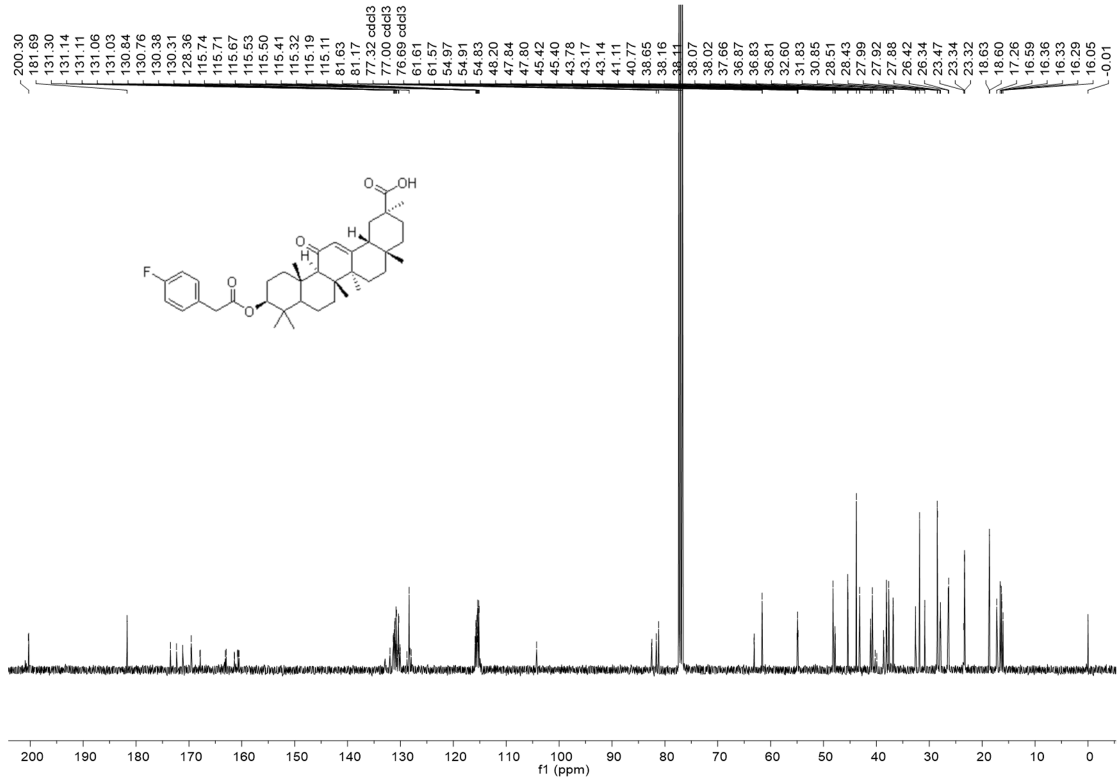

Supplement: Supplementary file 1 [file molecules-24-03631-s001.pdf]
